# Supplementary material for: Felis Catus Optimization (FCO): A novel nature‑inspired metaheuristic algorithm
Source: PLoS One. 2026 Apr 15;21(4):e0341325. doi: 10.1371/journal.pone.0341325 (PMC13082733; doi:10.1371/journal.pone.0341325)
Supplement: S1 Appendix — (DOCX) [file pone.0341325.s001.docx]

**Appendix S1 - Algorithm Parameter Settings and Experimental Budget**

This appendix summarizes the parameter configurations and experimental budgets adopted for a fair comparison between FCO and all competing metaheuristic algorithms.

All algorithms were executed under identical conditions (30 independent runs, same population size = 80, and termination criterion = 1000 iterations) on the benchmark sets (CEC 2005/2017) and engineering problems.

Default parameter values were taken from their original references, summarized in Table 23.

**Table 23.** Default parameter values for all algorithms.

| No | Algorithm | Key Parameters |
| --- | --- | --- |
| 1 | Cheetah Optimizer (ChOA) | C_Max=2, C_min=0.2 |
| 2 | Covariance Matrix Adaptation Evolution Strategy (CMA‑ES) | λ = 80 (offspring size); σ₀ = 0.3 (initial step size); damps = 1; c₁ = 2 (code‑specific scaling) |
| 3 | Exploration–Exploitation Balanced Correlation Matrix Adaptive Ranking (EBO-CMAR) | α = 0.9; γ = 0.8 |
| 4 | Coyote Optimization Algorithm (COA) | N_packs = 5; N_c = 16 (coyotes per pack) |
| 5 | Differential Evolution (DE) | F = 0.5 (scaling factor); C_R = 0.9 (crossover rate); strategy = DE/rand/1/bin |
| 6 | Linear Population Size Reduction SHADE (L-SHADE) | Nmin = 4; H = 10; MF = 0.5; MCR = 0.5  Note: Ninit omitted (population). H is memory size; MF/MCR are initial means for F and CR adaptation |
| 7 | Grey Wolf Optimizer (GWO) | a = 2; A1 = 2; C1 = 2; A2 = 2; C2 = 2; A3 = 2; C3 = 2  Note: a (and A,C triplets) start at 2 and decrease/update per standard GWO dynamics |
| 8 | Particle Swarm Optimization (PSO) | c1 = 2; c2 = 2; w_max = 0.9; w_min = 0.4; Vmax = 0.2 |
| 9 | Manta Ray Foraging Optimization (MRFO) | beta = 2; c1 = 2; c2 = 2  Note: Exploitation/exploration coefficients as implemented in code |
| 10 | Equilibrium Optimizer (EO) | a1 = 2; a2 = 1; GP = 0.5  Note: a1/a2 shape the generation rate; GP is the generation probability |
| 11 | Reptile Search Algorithm (RSA) | α = 3, β = 0.8, QF = exp(–(4 t/MaxIt)²) |
| 12 | Runge–Kutta Optimizer (RUN) | β = 1.5, F = 2, pᵤ = 0.3 |
| 13 | Quantum–Invasive Optimization (QIO) | beta = 0.5; alpha = 1.5; q_rate = 0.7; |
| 14 | Sand Cat Optimizer (SCO) | SRmax = 2, SRmin = 0.001, β = 2 → 0 (linear) |
| 15 | Sine Cosine Algorithm (SCA) | r₁(t) = 2 – 2 × (t/MaxIt) (linearly decreasing amplitude)  r₂,r₃,r₄∈U(0,1) (random phase/weight terms) |
| 16 | Whale Optimization Algorithm (WOA) | a = 2 – 2 × (t/MaxIt) (linear contraction)  A = 2a r₁ – a, C = 2 r₂ (encircling coefficients)  b = 1, l ∈[–1,1] for spiral bubble‑net feeding. |
| 17 | Salp Swarm Algorithm (SSA) | Population split: Leader + Follower chains  c₁ = 2 × exp(–(4 t/MaxIt)²) (decaying exploration constant)  Random vectors c₂,c₃∈[0,1] for position update weights. |

All algorithms were implemented in MATLAB R2019a on a PC with Intel® Core™ i5‑3230M CPU (2.60 GHz,4 GB RAM, Windows 7 x64), ensuring full reproducibility.
