# Supplementary material for: Felis Catus Optimization (FCO): A novel nature‑inspired metaheuristic algorithm
Source: PLoS One. 2026 Apr 15;21(4):e0341325. doi: 10.1371/journal.pone.0341325 (PMC13082733; doi:10.1371/journal.pone.0341325)

**Appendix S2 – Convergence Curves (CEC 2005 Benchmark Suite)**

The following figures illustrate the mean ± standard deviation of convergence trajectories for all 17 CEC 2005 benchmark functions. Each curve represents the average objective value over ependent runs per algorithm, plotted on a logarithmic scale (log error vs. iterations). All algorithms were tested under identical settings (population = 80, max iterations = 1000, D = 30).


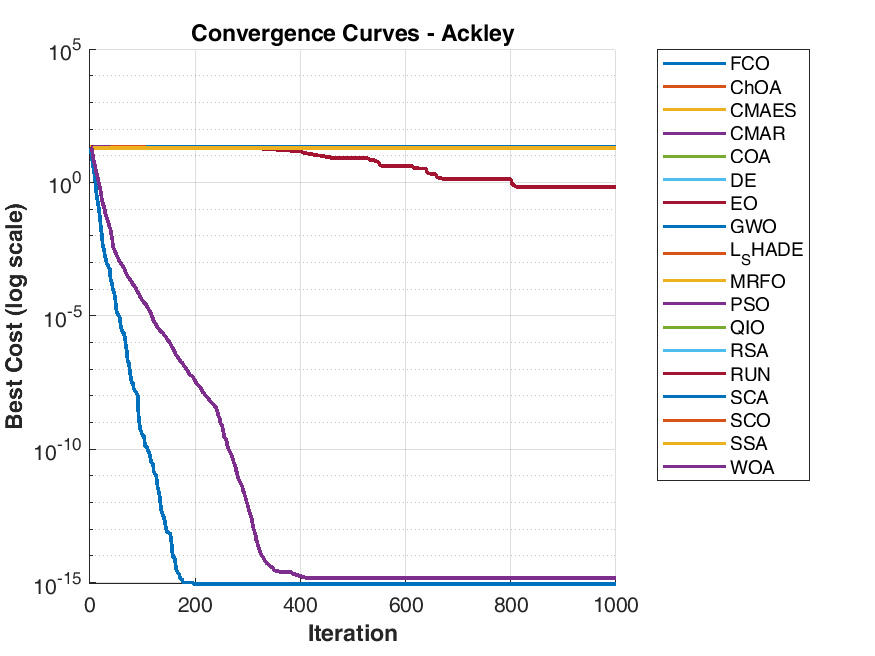

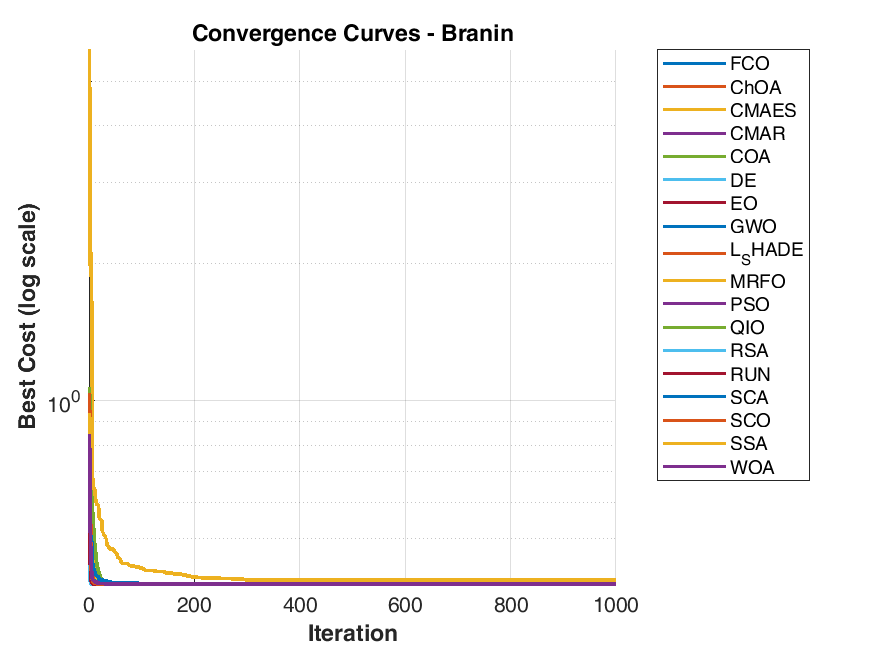

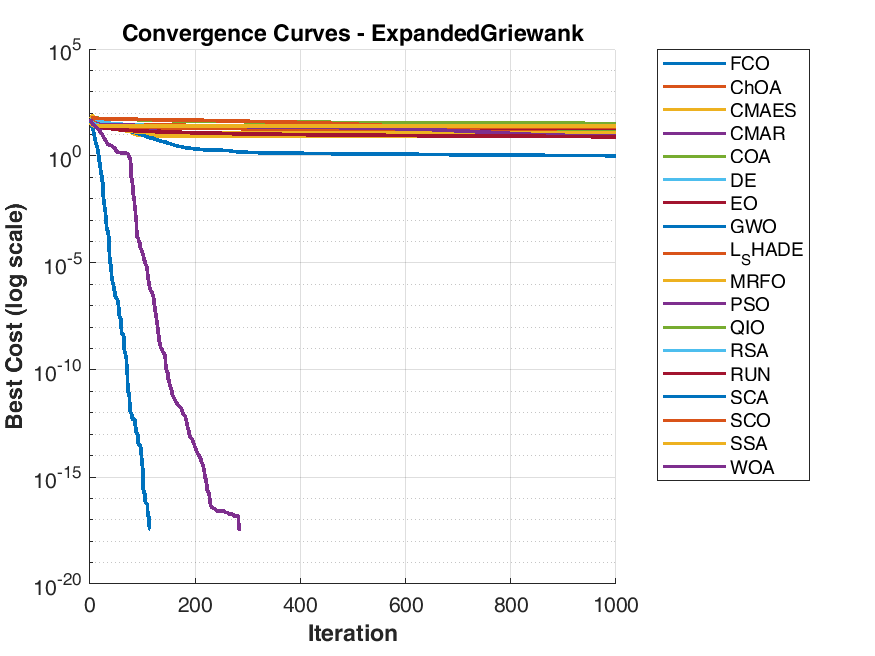

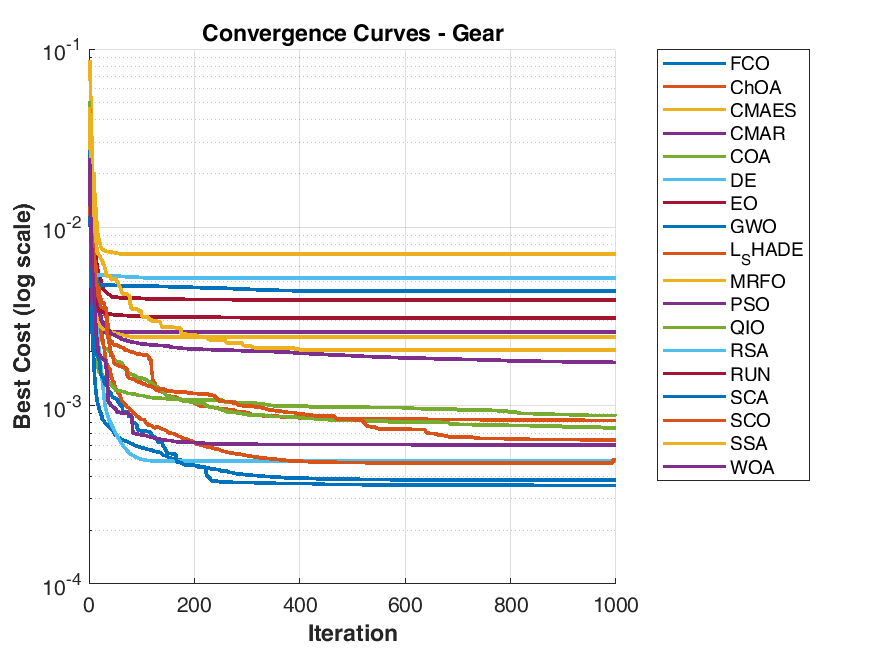

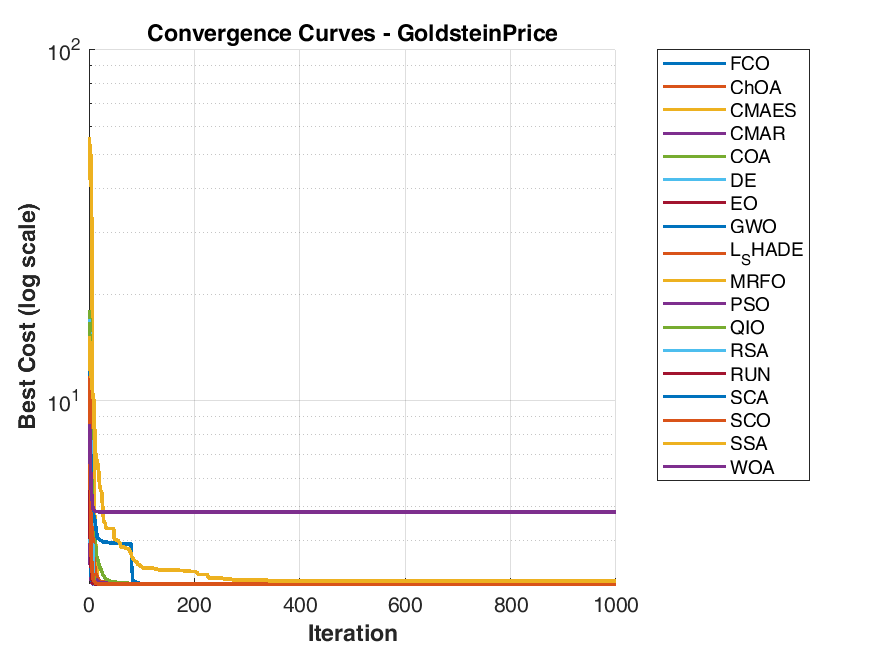

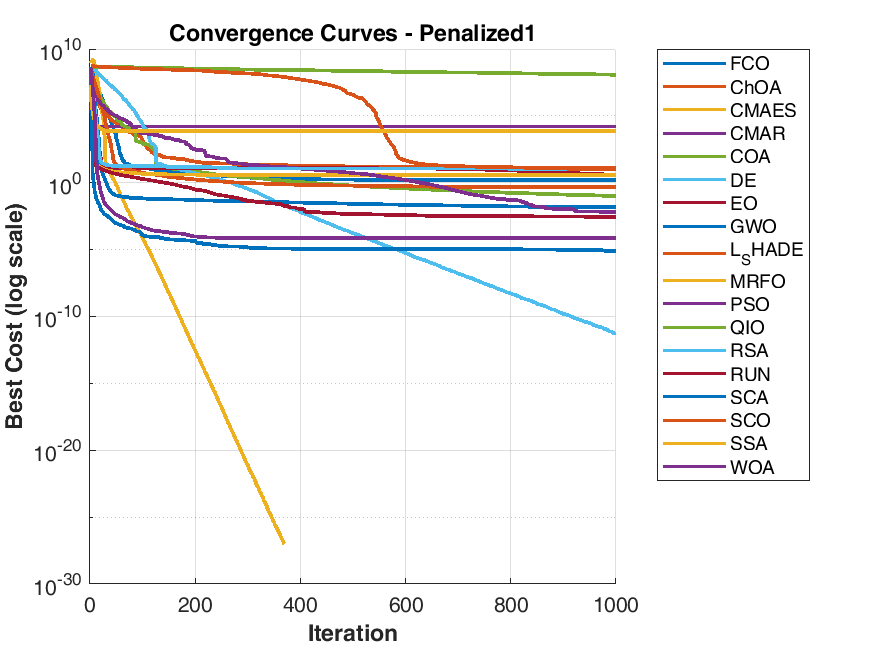

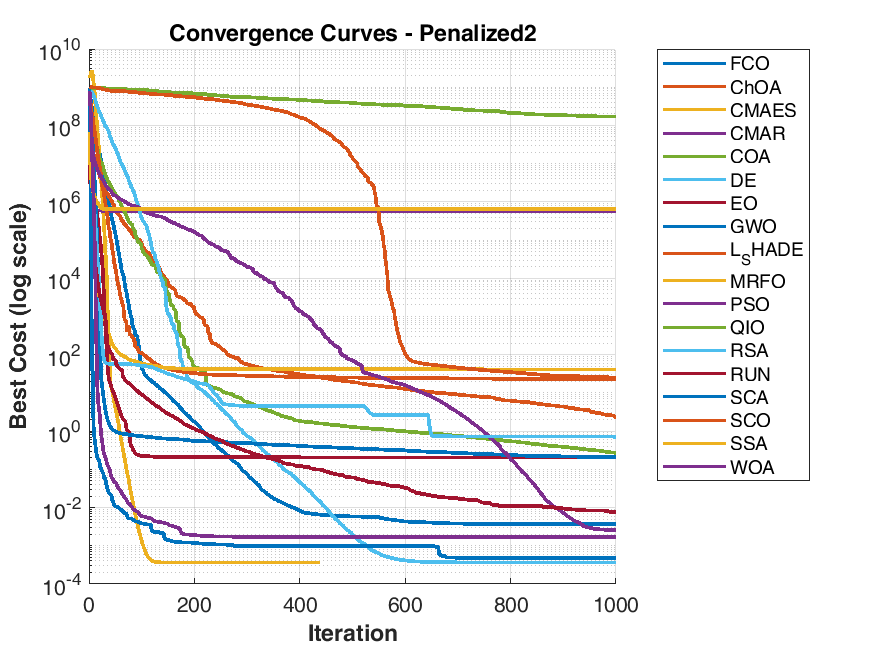

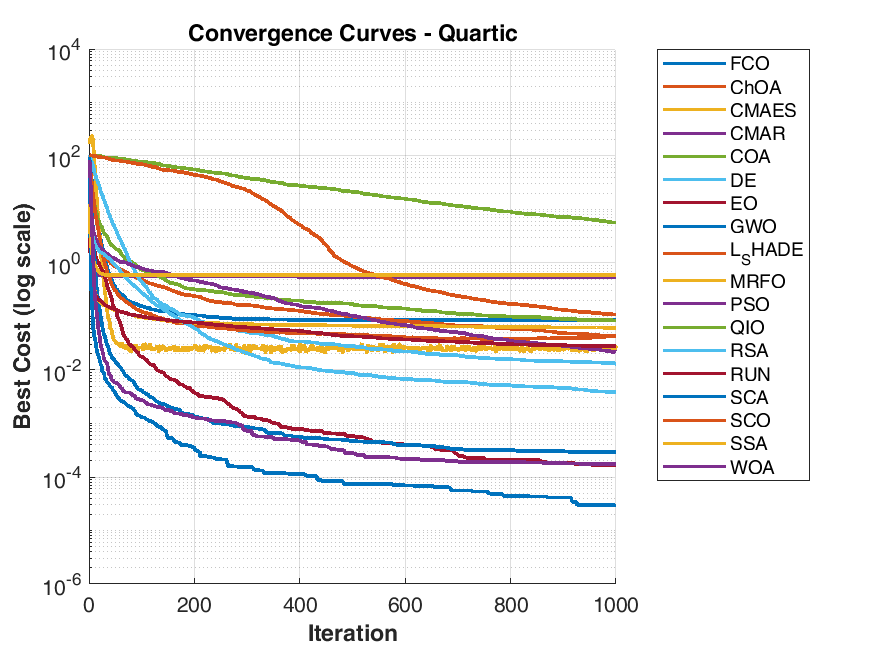

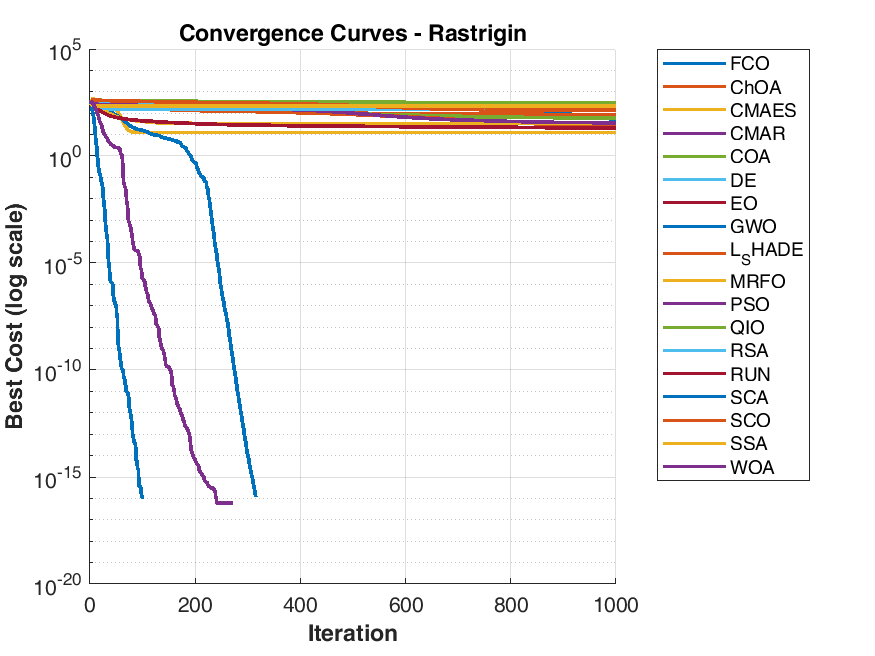

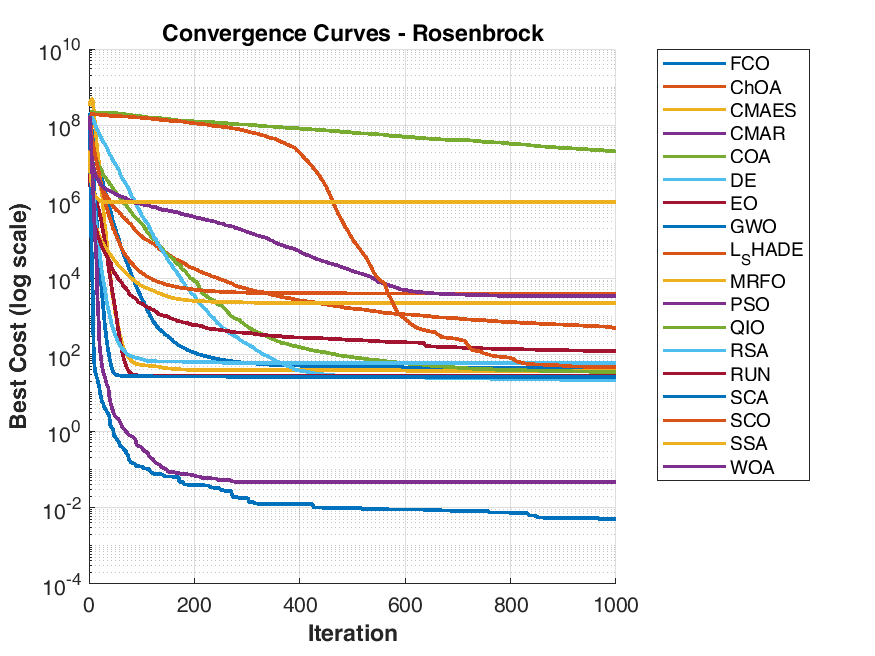

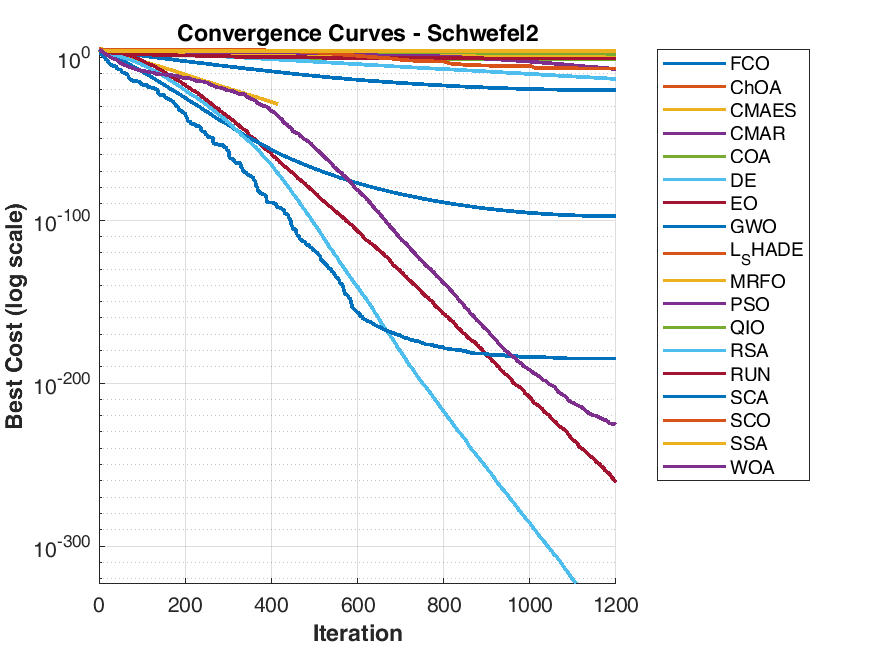

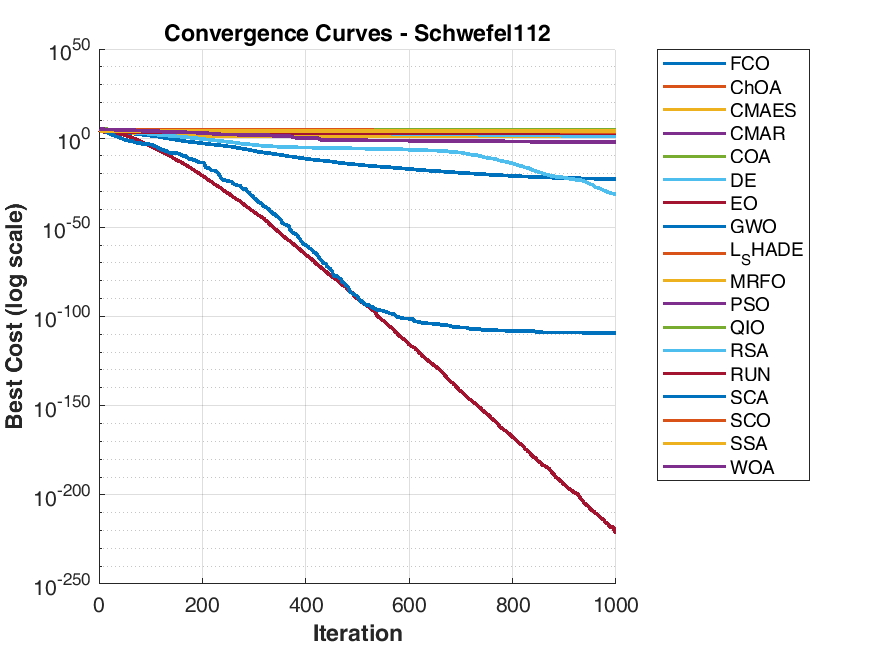

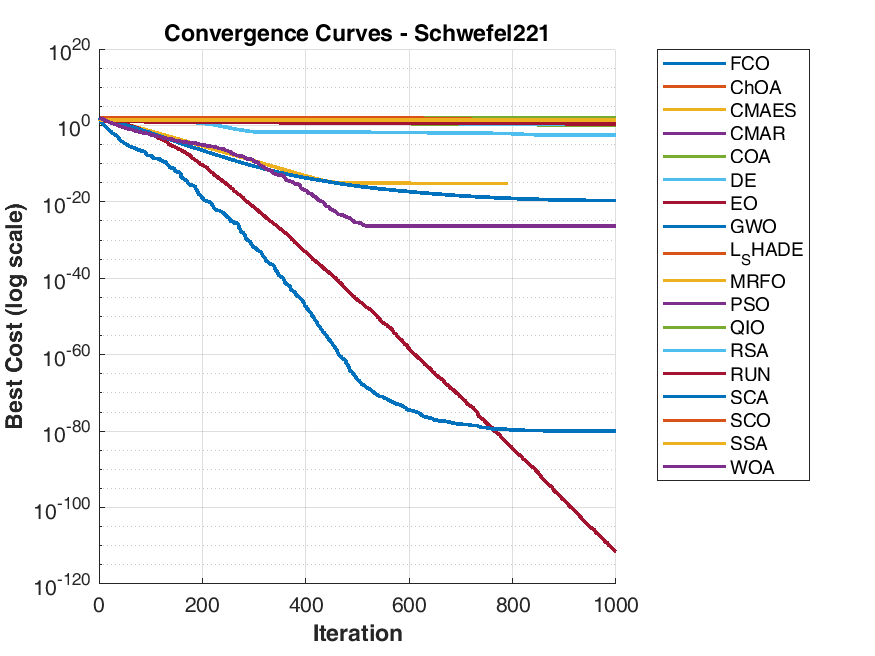

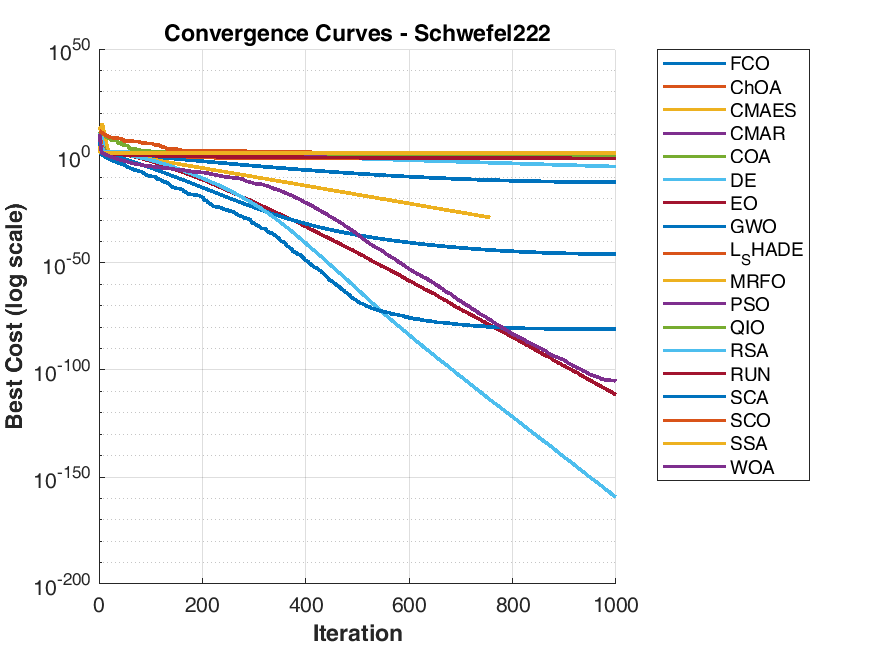

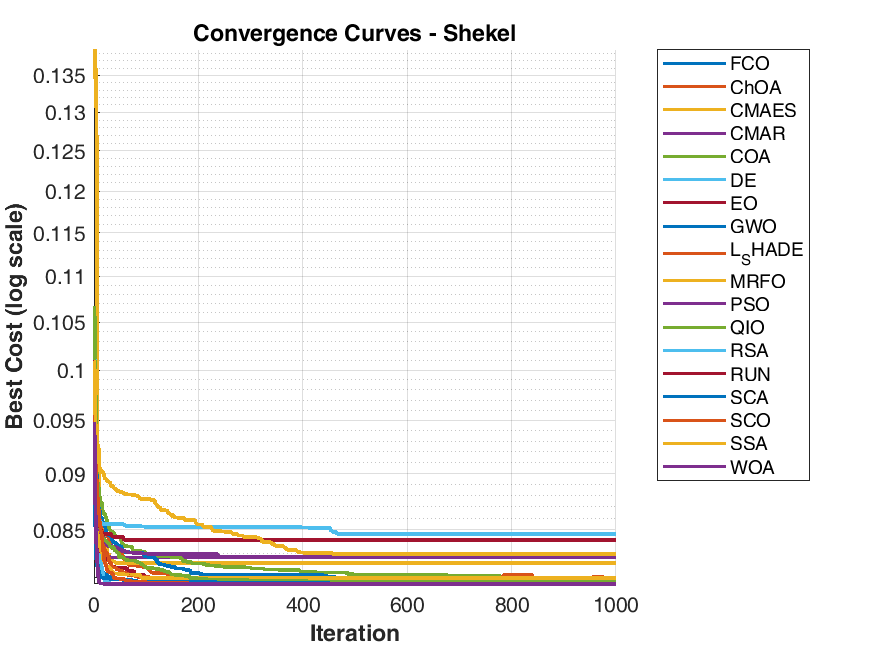

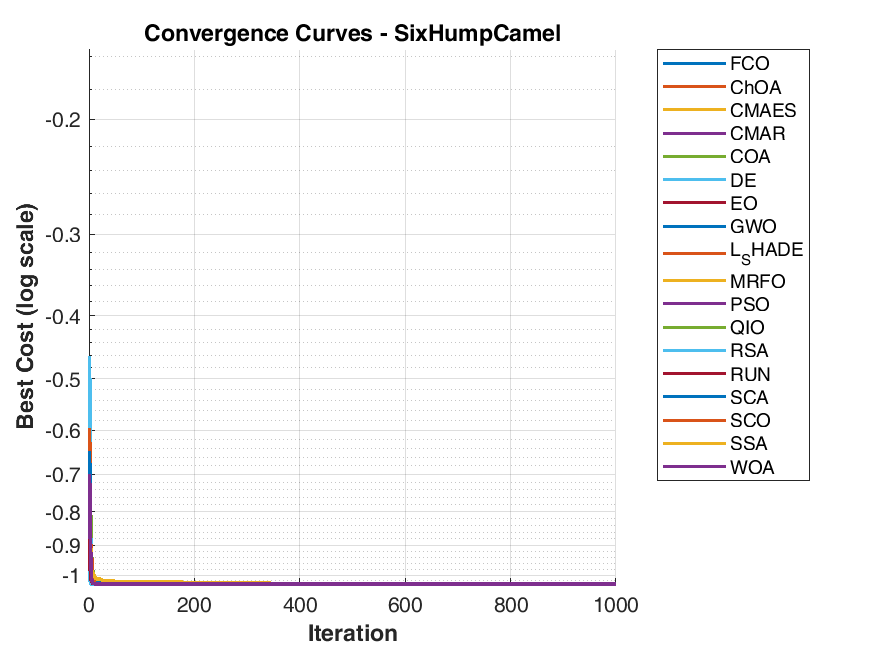

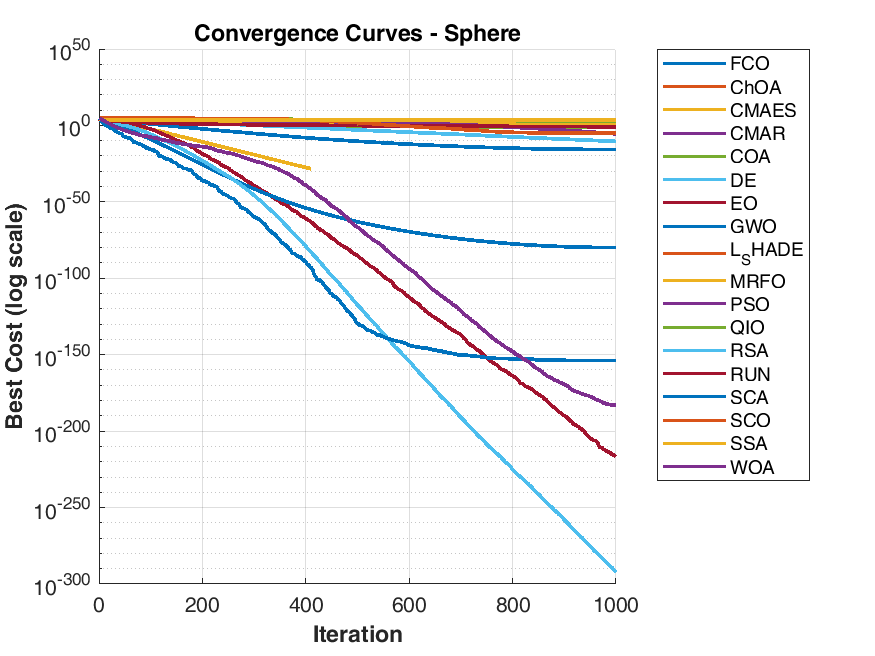

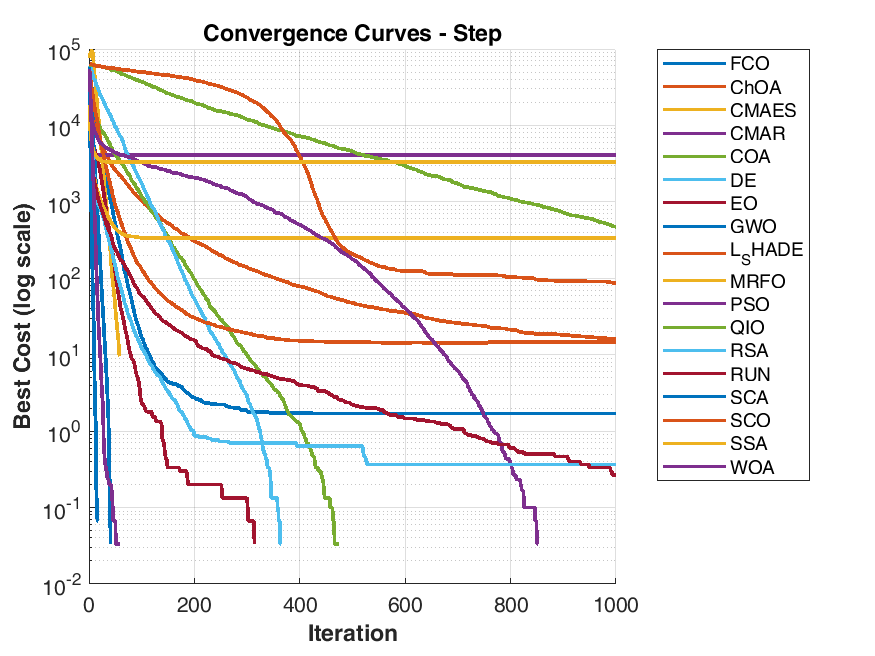

Supplement: S2 Appendix — (DOCX) [file pone.0341325.s002.docx]
