# Supplementary material for: Felis Catus Optimization (FCO): A novel nature‑inspired metaheuristic algorithm
Source: PLoS One. 2026 Apr 15;21(4):e0341325. doi: 10.1371/journal.pone.0341325 (PMC13082733; doi:10.1371/journal.pone.0341325)

**Appendix S3 – Convergence Curves (CEC 2017 Benchmark Suite)**

This appendix presents convergence trajectories for 30 benchmark functions of CEC 2017. Each subplot shows the averaged performance of FCO and 17 comparative algorithms across 30 independent runs using standard CEC 2017 parameter settings. The y‑axis denotes the log‑scaled error value, and the x‑axis indicates iteration count (from 1 to 1000).


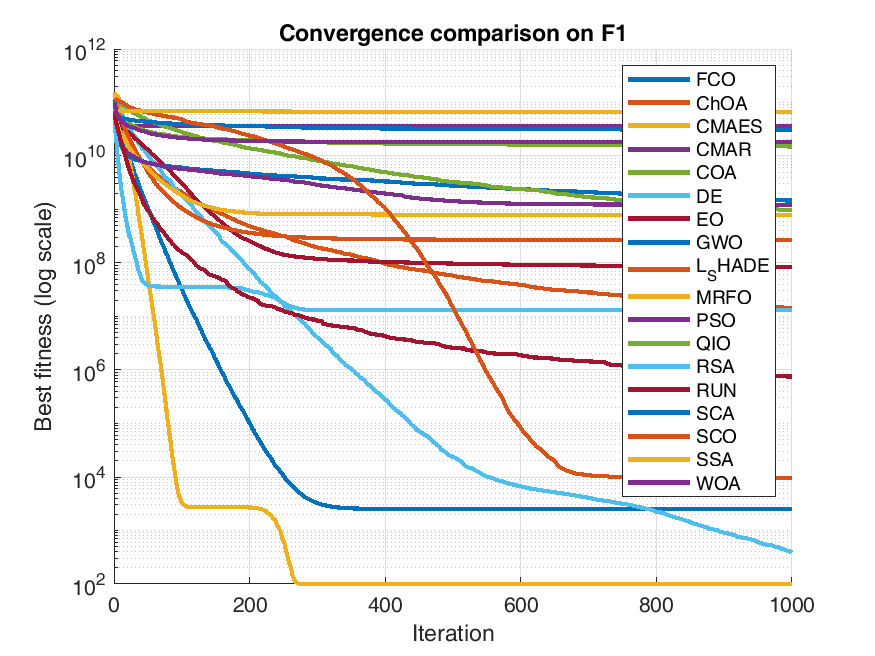

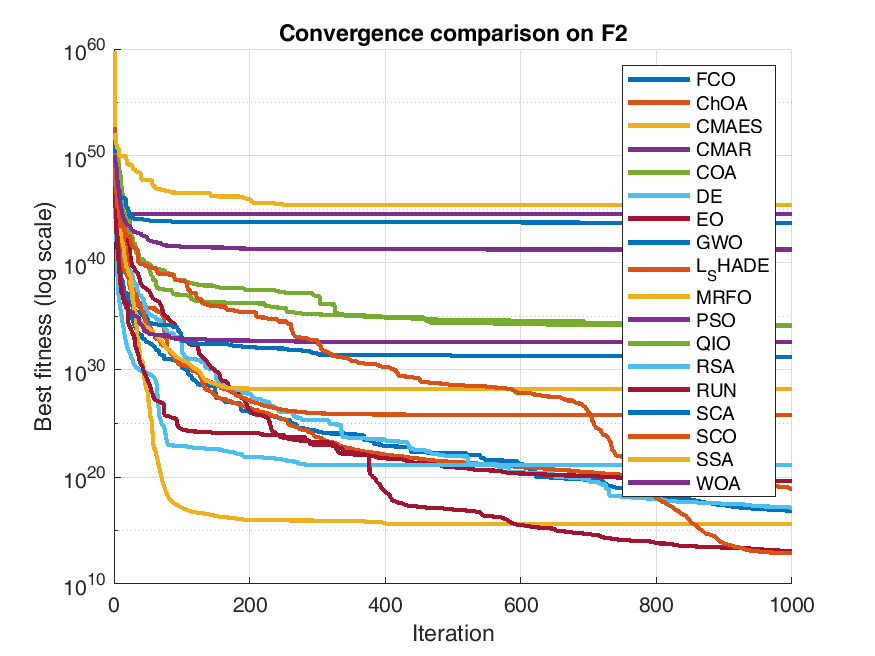

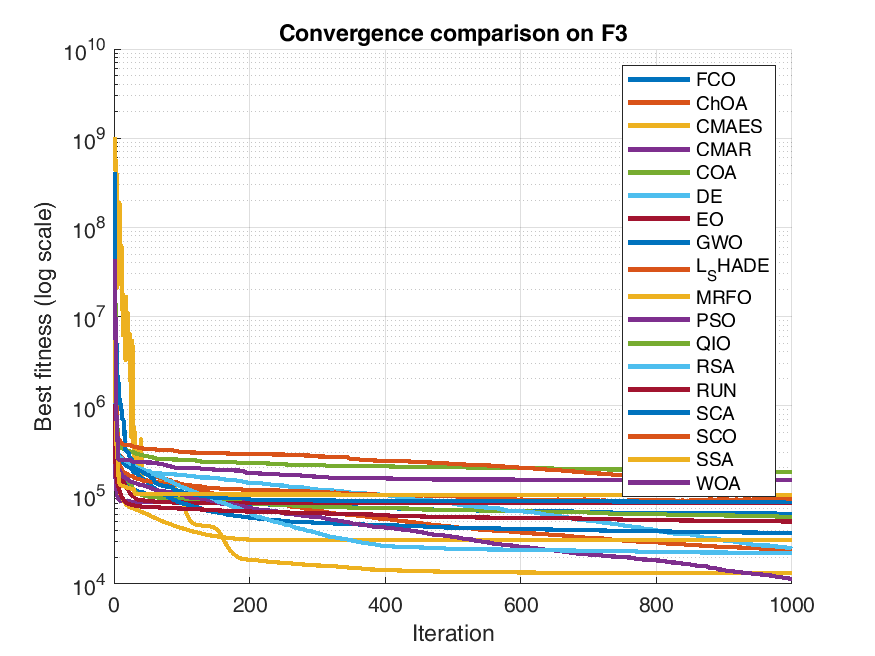

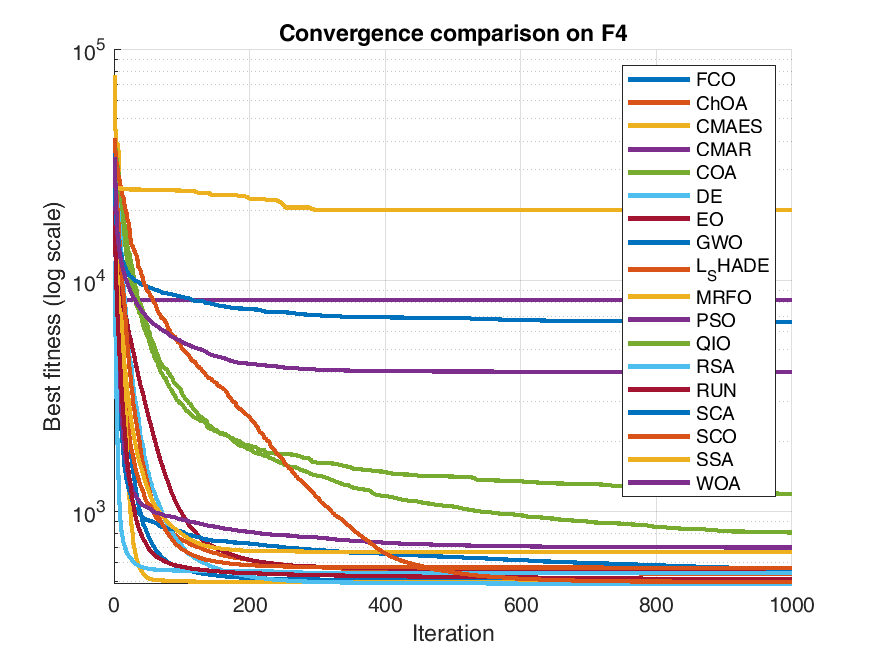

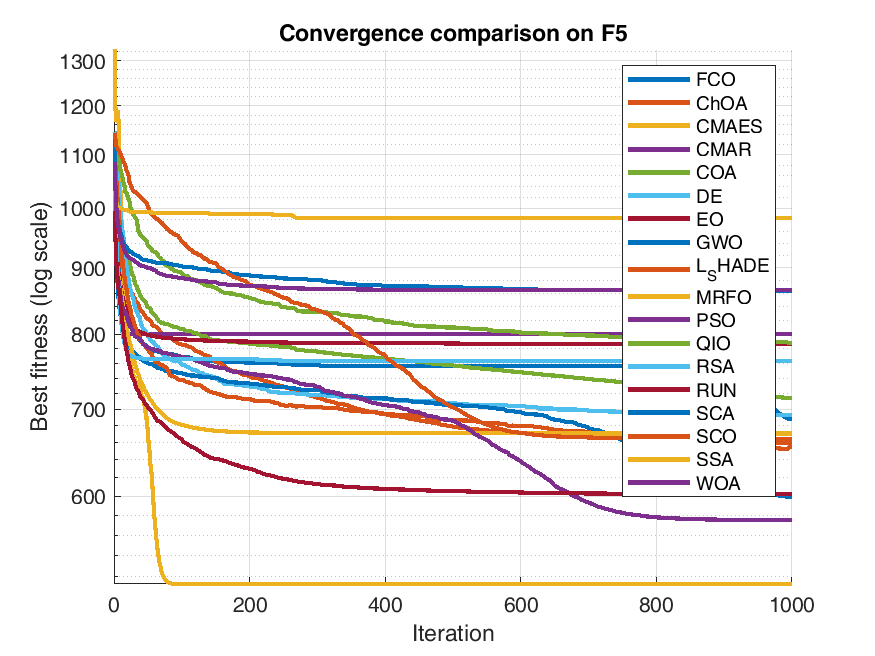

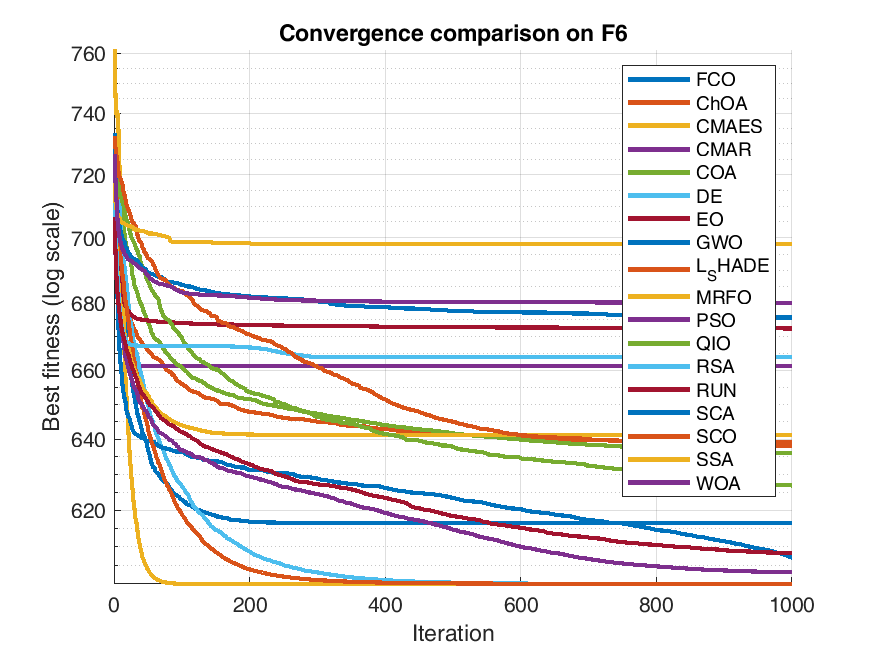

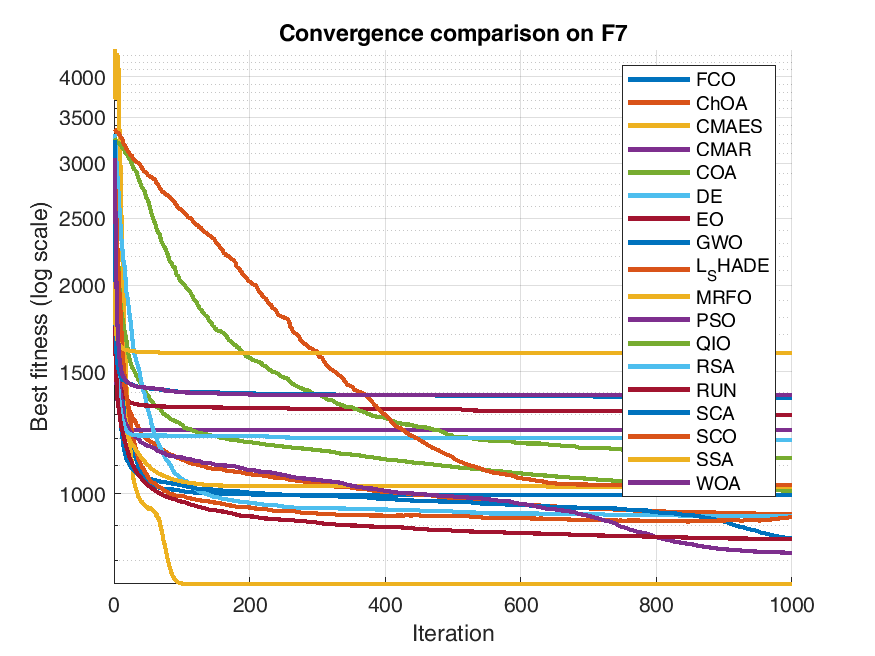

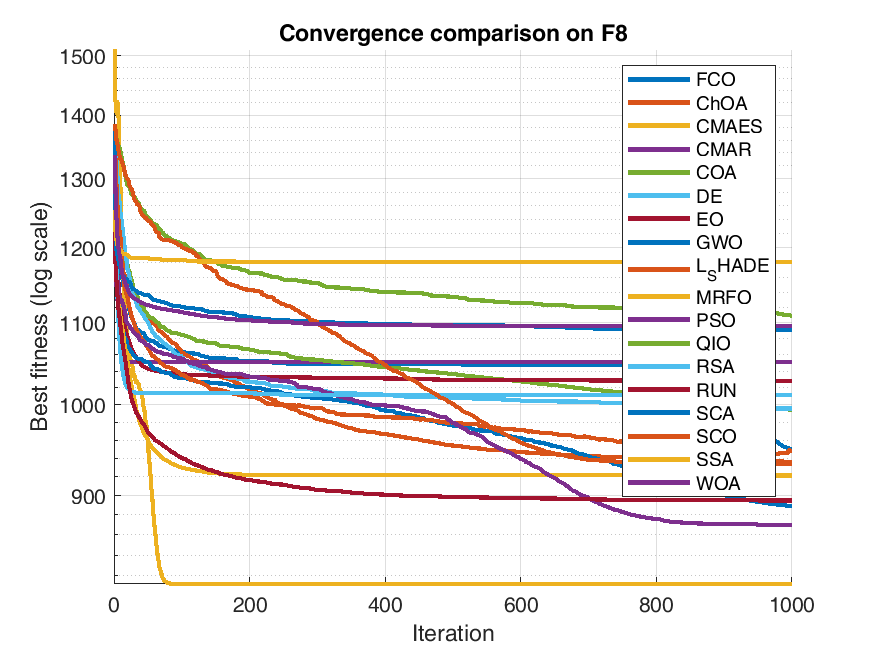

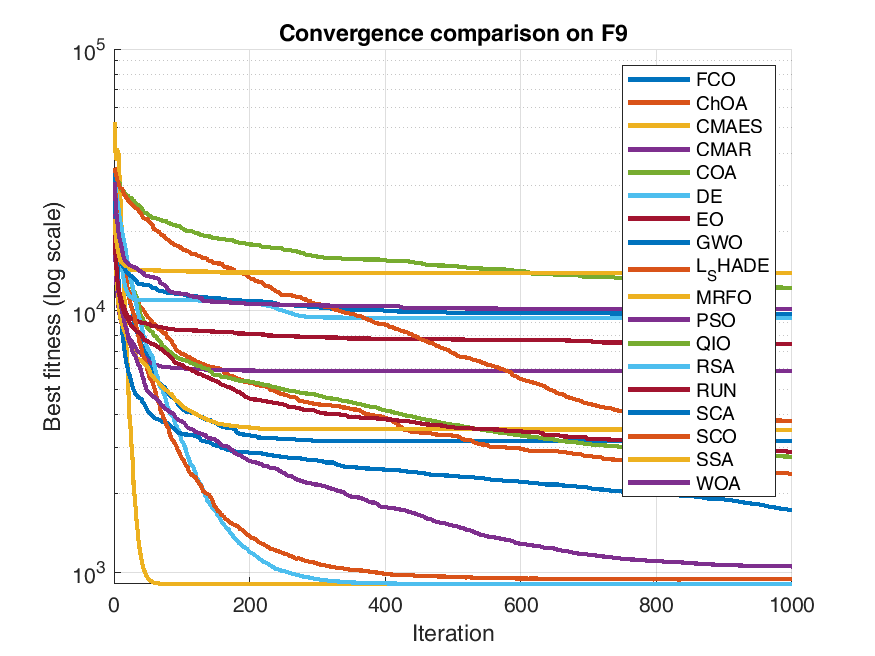

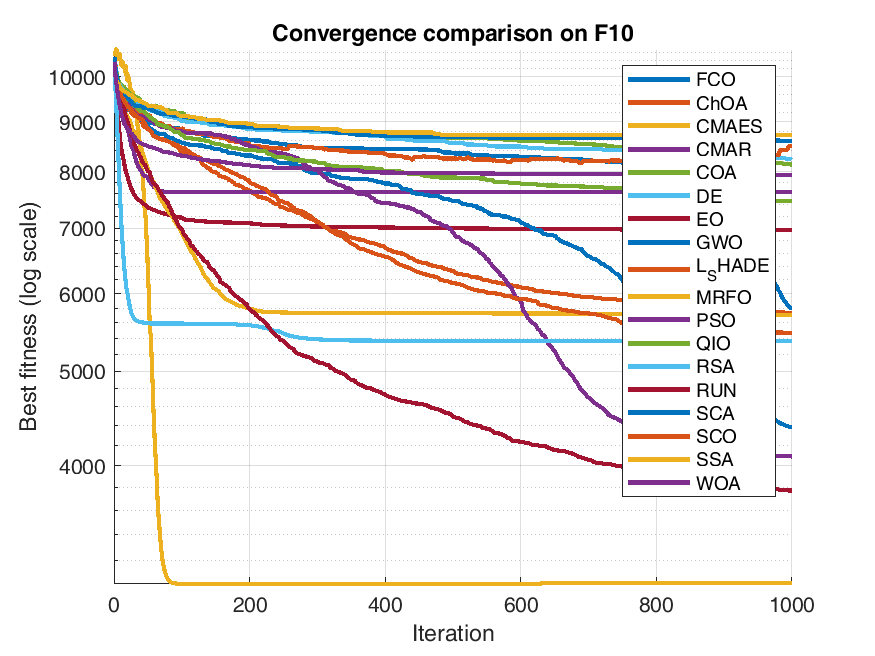

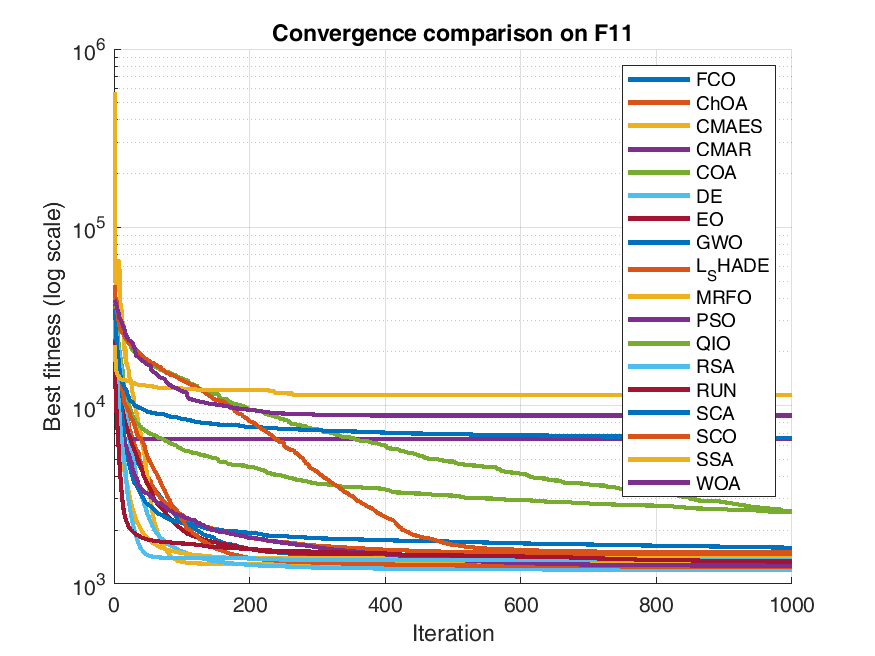

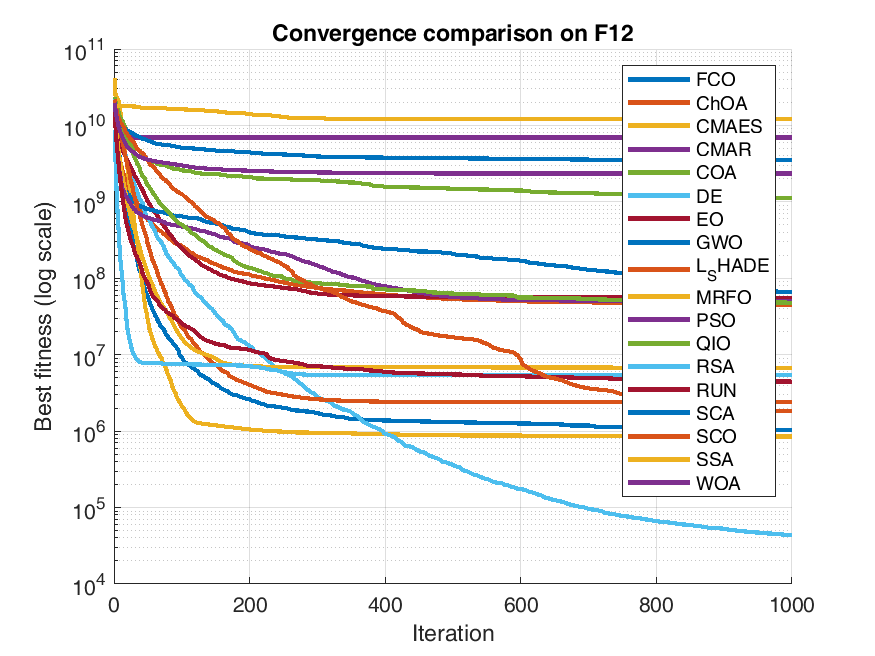

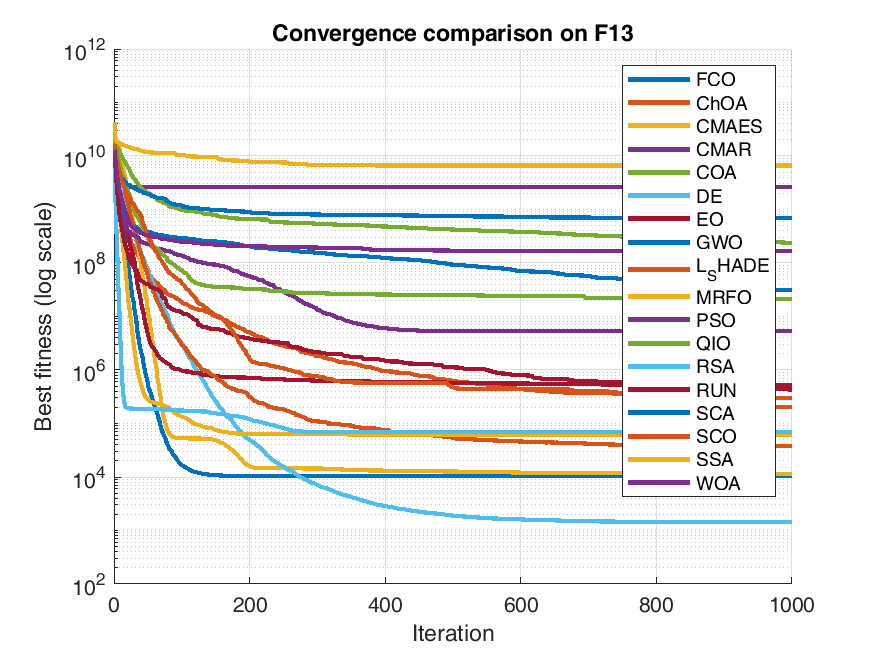

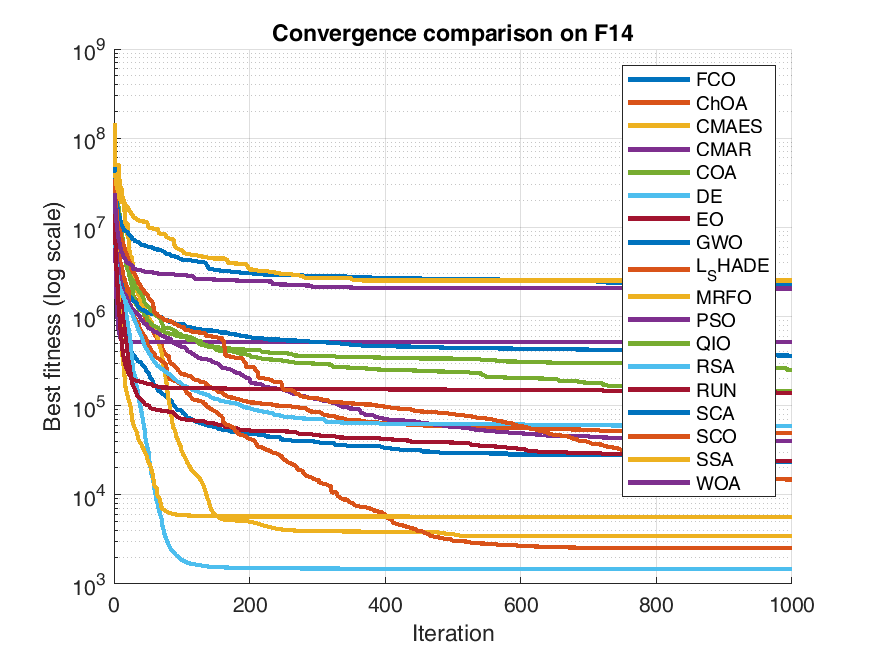

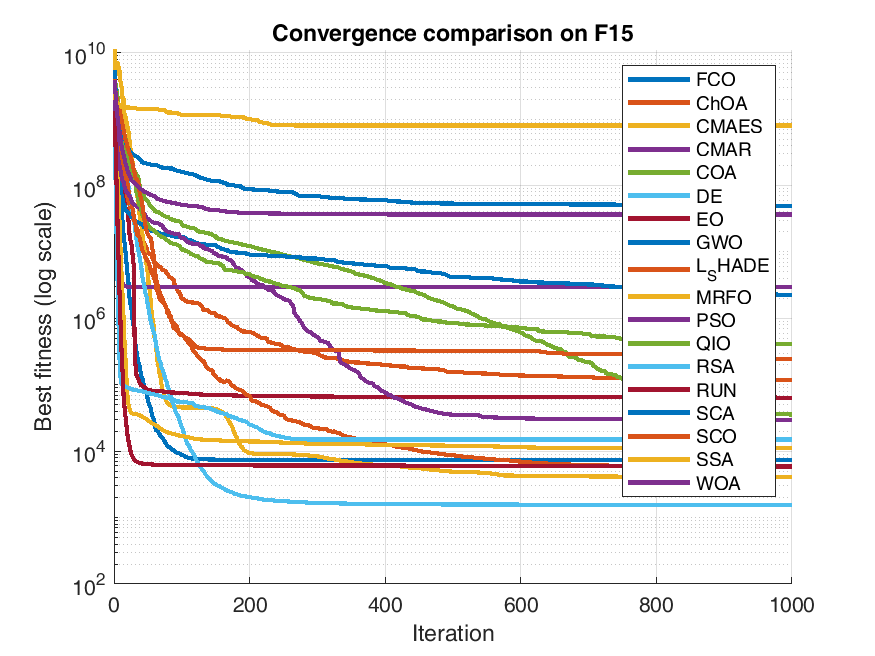

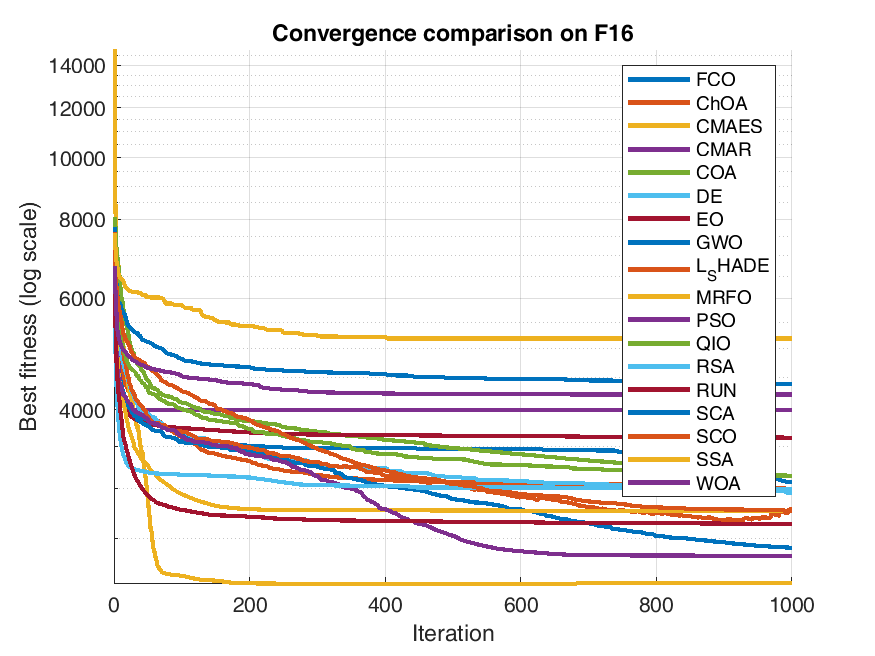

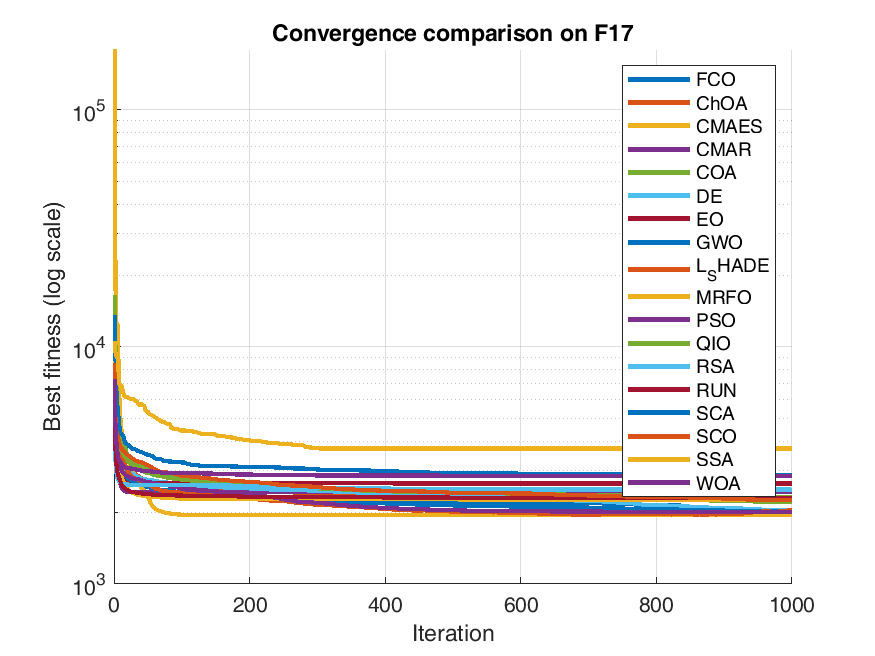

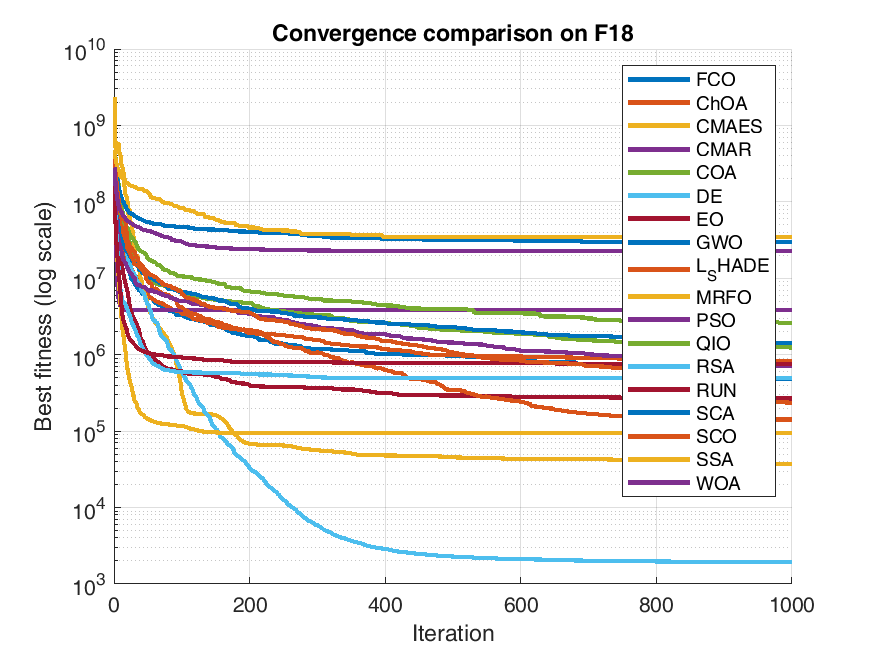

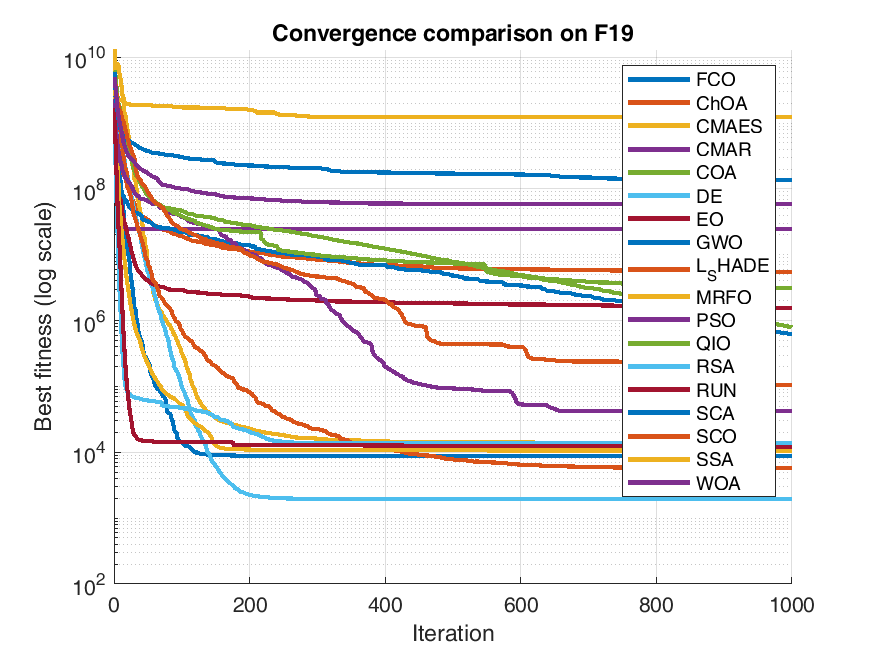

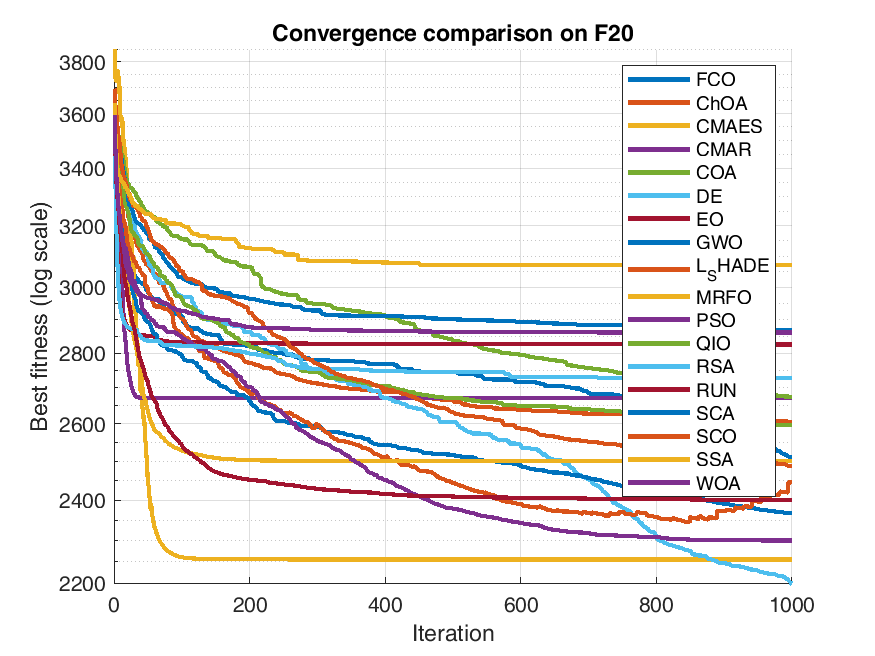

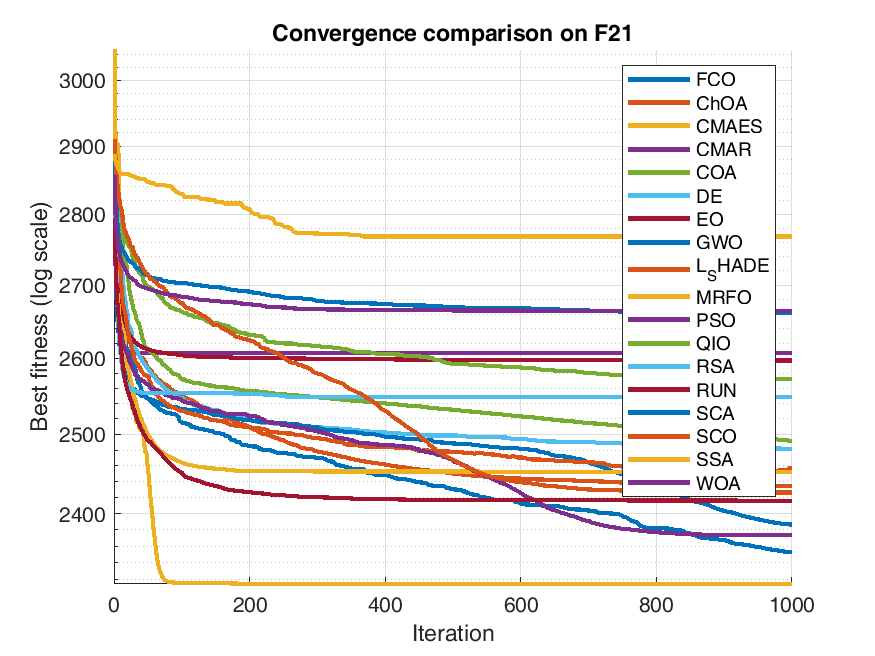

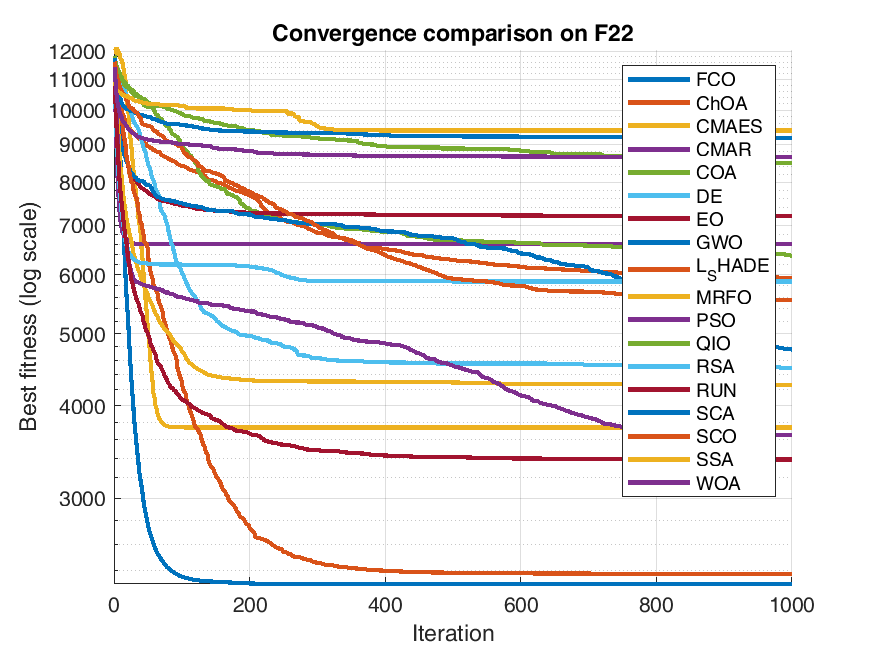

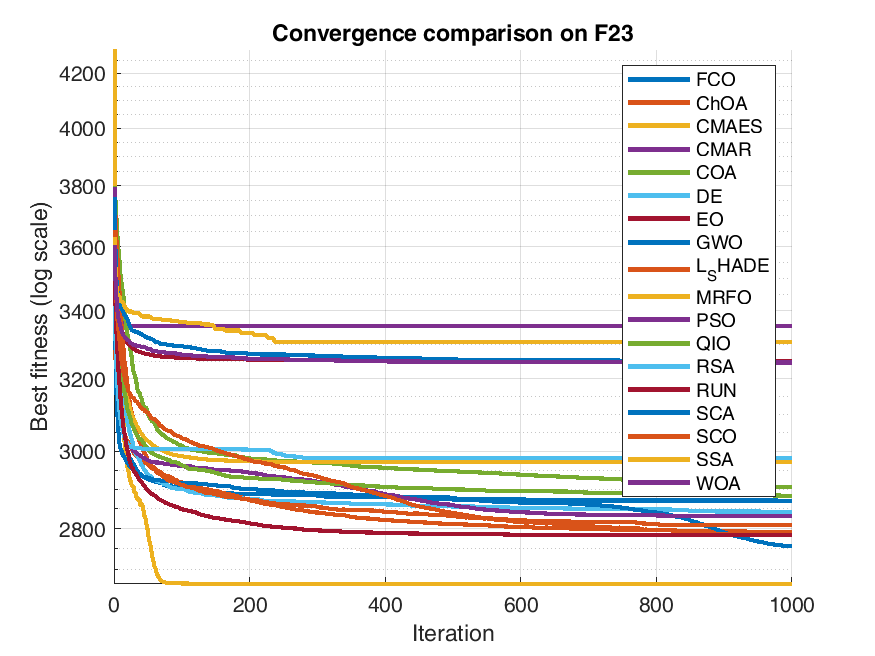

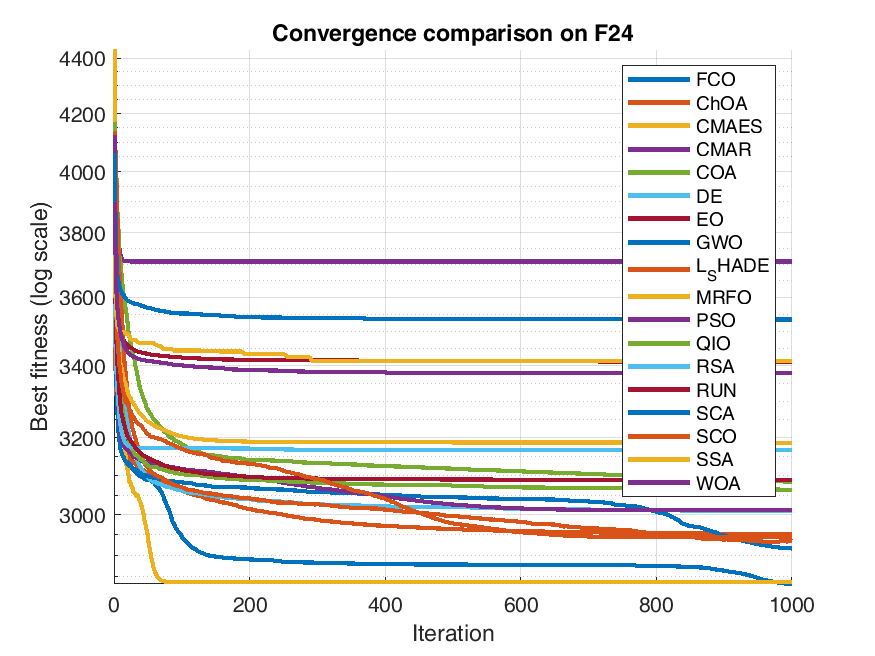

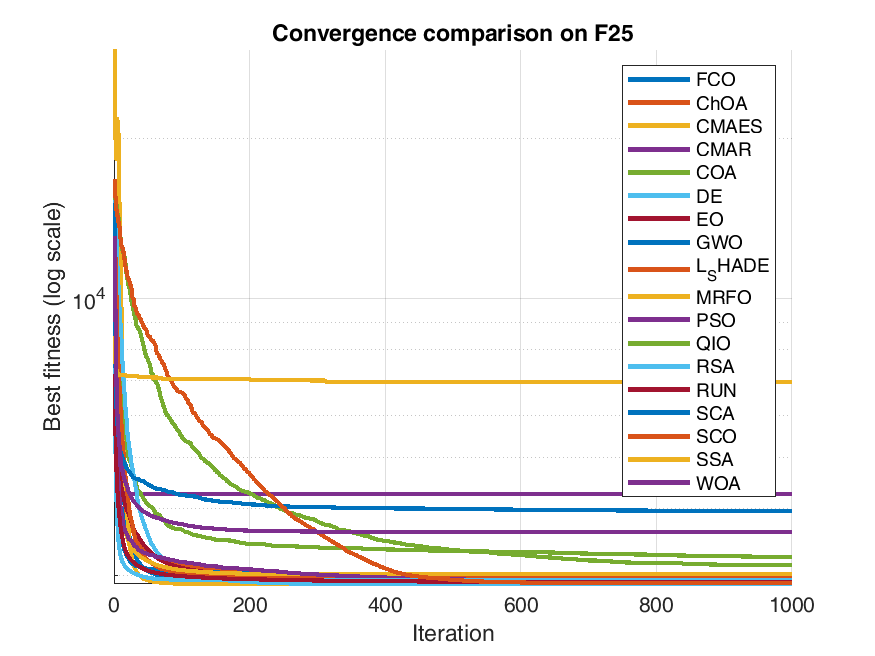

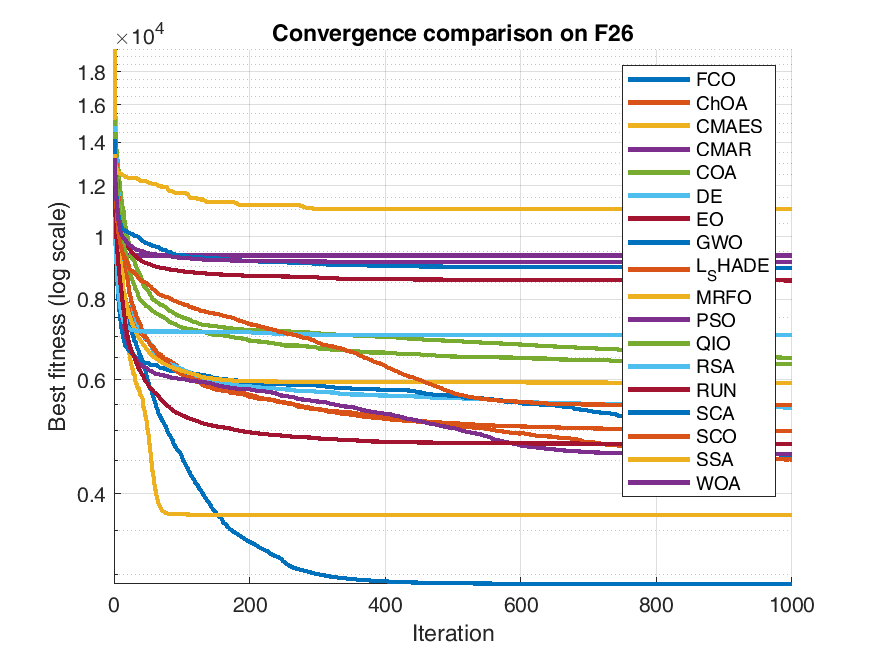

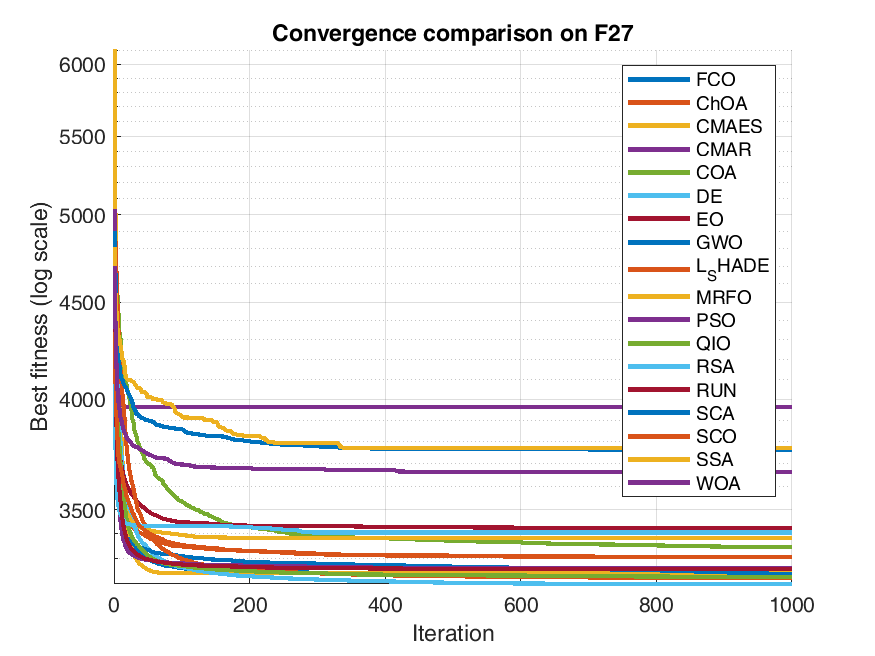

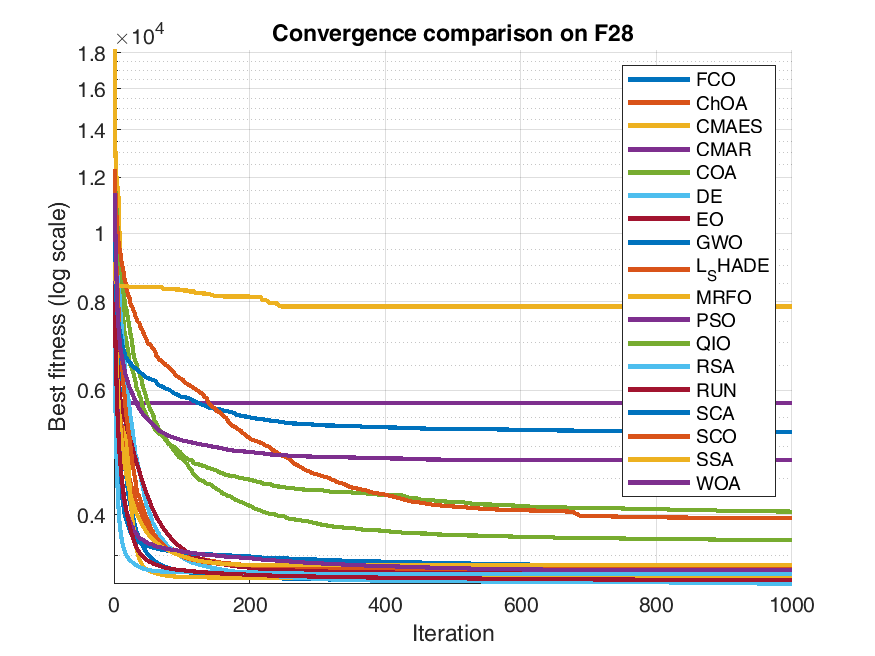

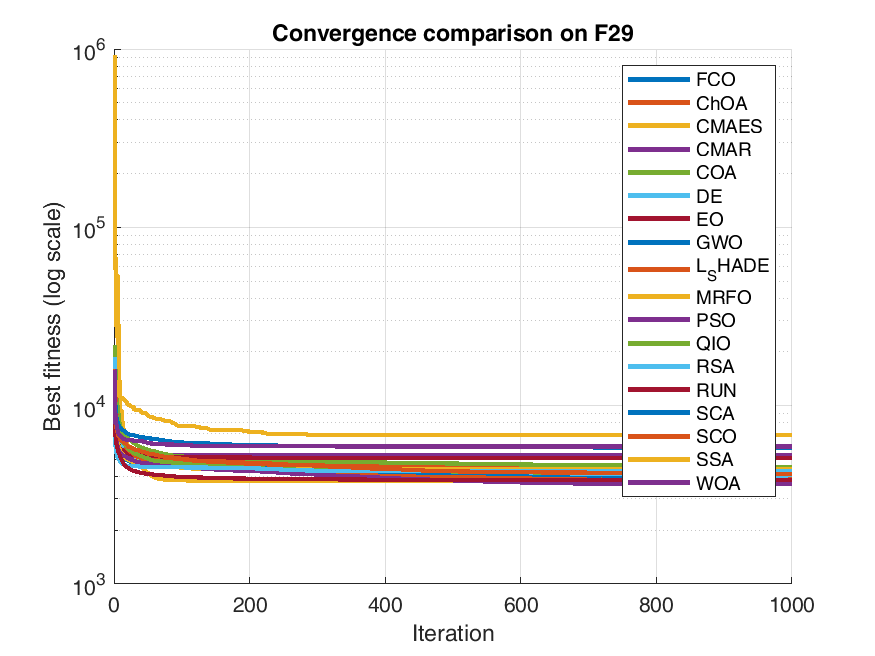

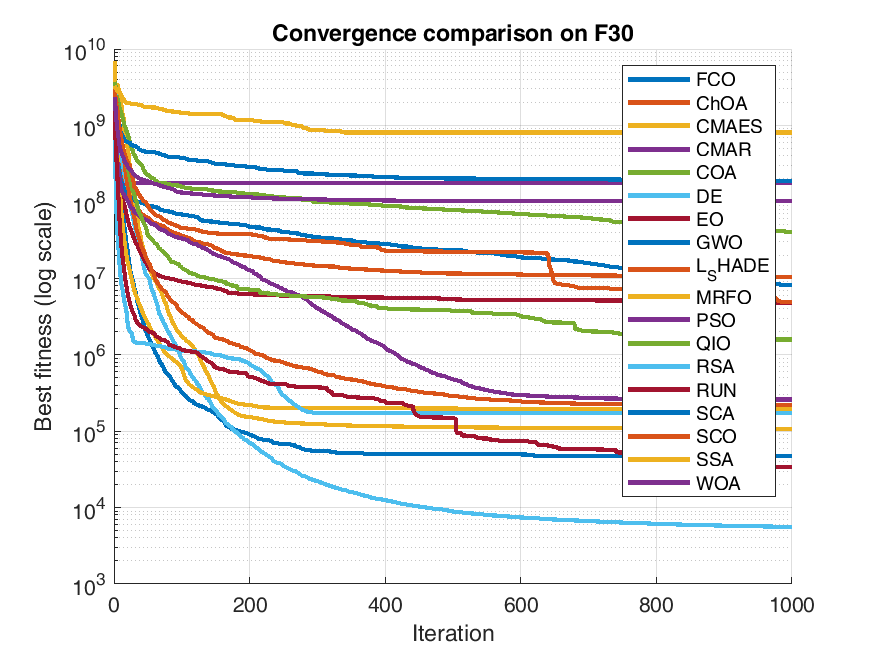

Supplement: S3 Appendix — (DOCX) [file pone.0341325.s003.docx]
