# Supplementary figures and images for: Felis Catus Optimization (FCO): A novel nature‑inspired metaheuristic algorithm
Source: PLoS One. 2026 Apr 15;21(4):e0341325. doi: 10.1371/journal.pone.0341325 (PMC13082733; doi:10.1371/journal.pone.0341325)

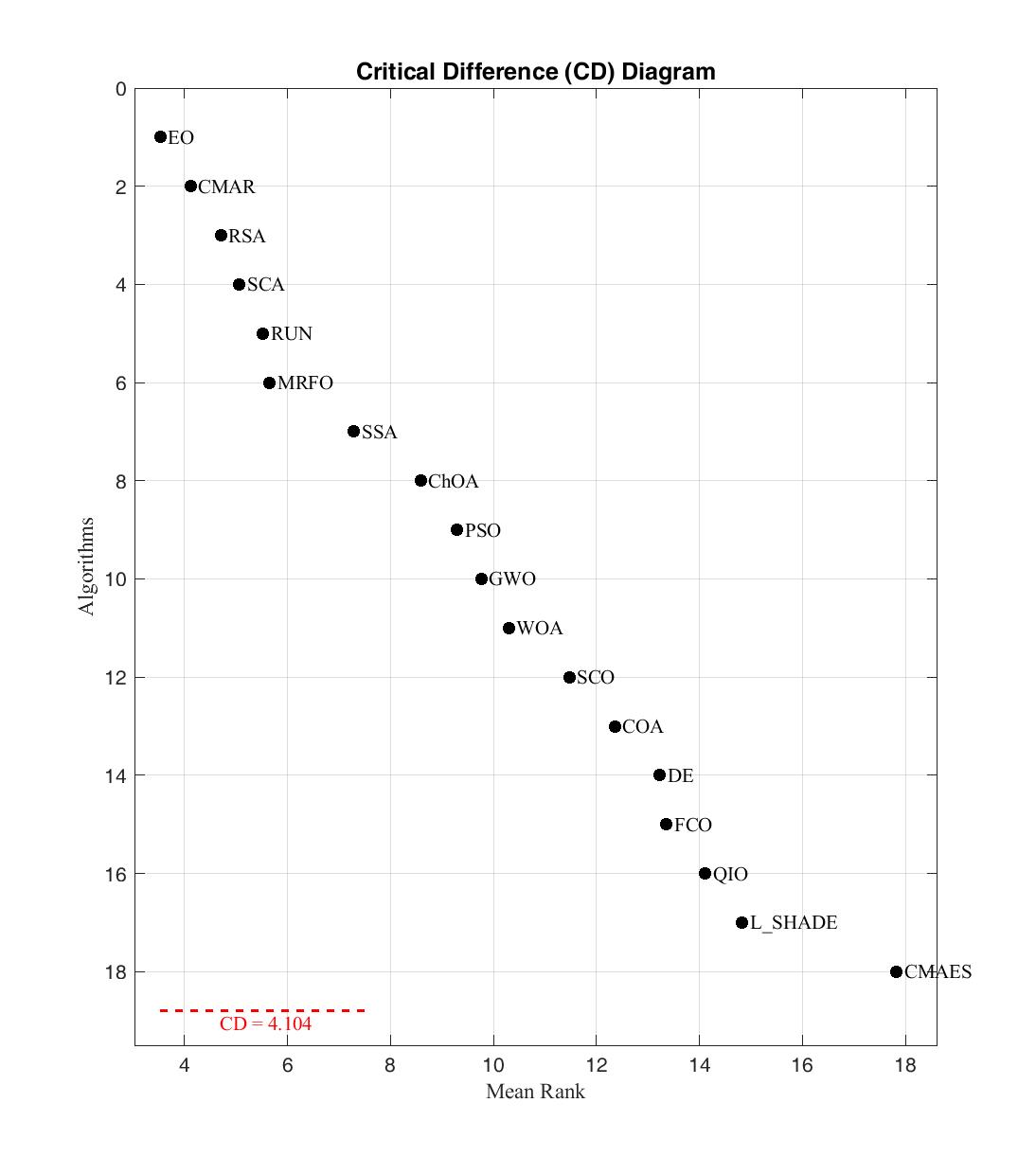

Supplement: S1 Data — (ZIP) [file pone.0341325.s004.zip › FCO Codes/CEC2005/Results/CDPlots/CD_Diagram_CEC2005_alpha0.05.jpg]

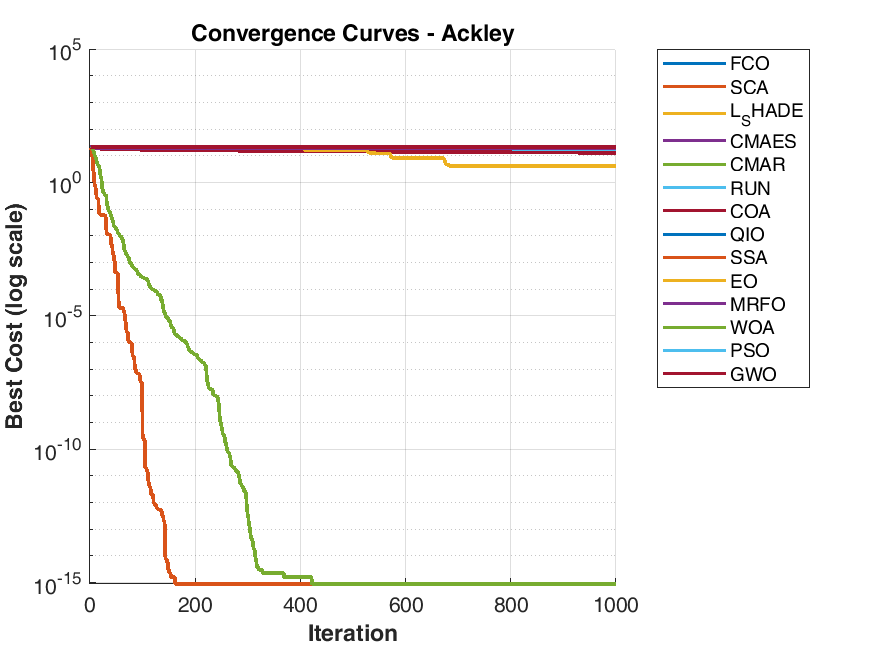

Supplement: S1 Data — (ZIP) [file pone.0341325.s004.zip › FCO Codes/CEC2005/Results/ConvergencePlots 1/Ackley_all_algorithms.png]

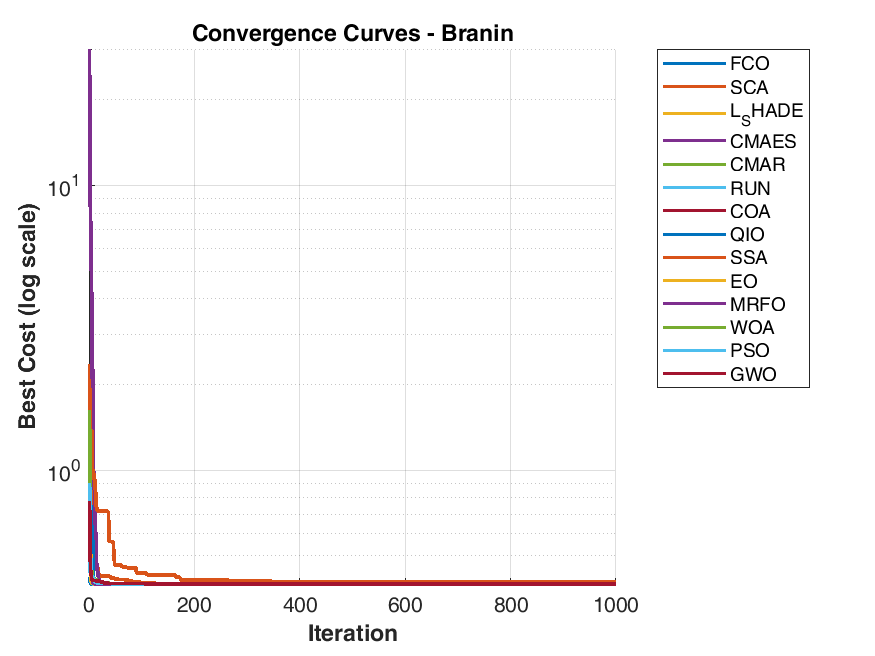

Supplement: S1 Data — (ZIP) [file pone.0341325.s004.zip › FCO Codes/CEC2005/Results/ConvergencePlots 1/Branin_all_algorithms.png]

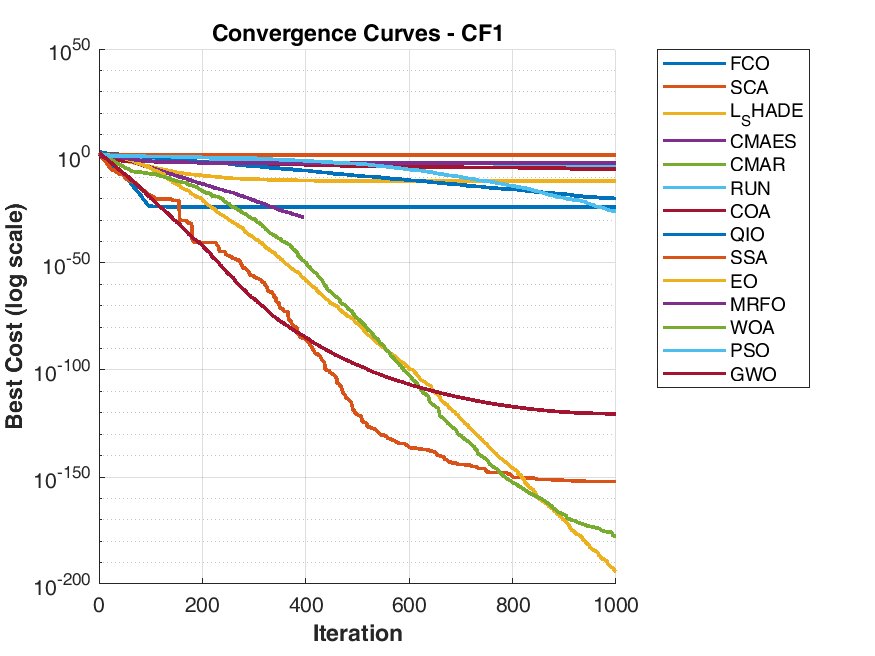

Supplement: S1 Data — (ZIP) [file pone.0341325.s004.zip › FCO Codes/CEC2005/Results/ConvergencePlots 1/CF1_all_algorithms.png]

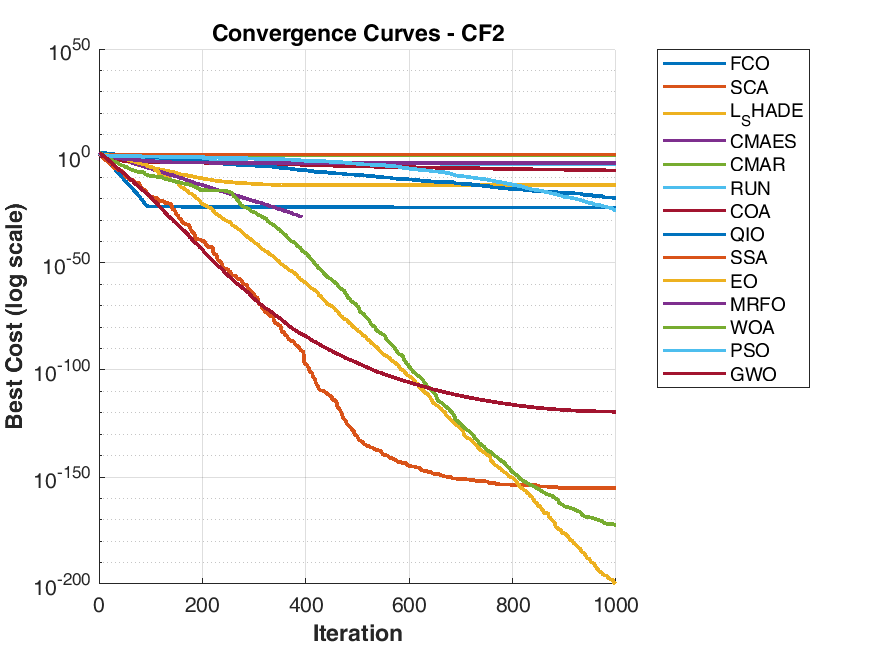

Supplement: S1 Data — (ZIP) [file pone.0341325.s004.zip › FCO Codes/CEC2005/Results/ConvergencePlots 1/CF2_all_algorithms.png]

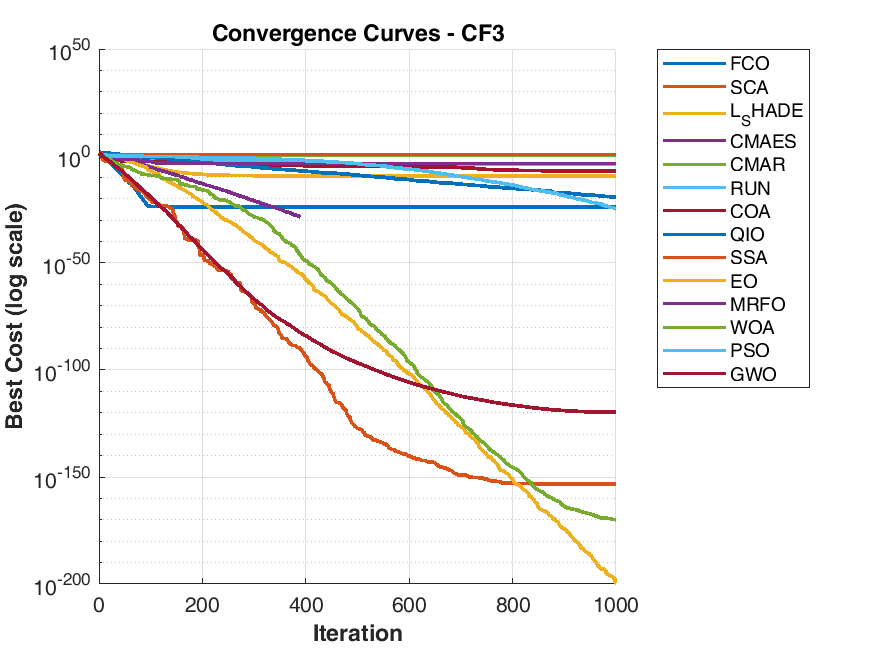

Supplement: S1 Data — (ZIP) [file pone.0341325.s004.zip › FCO Codes/CEC2005/Results/ConvergencePlots 1/CF3_all_algorithms.png]

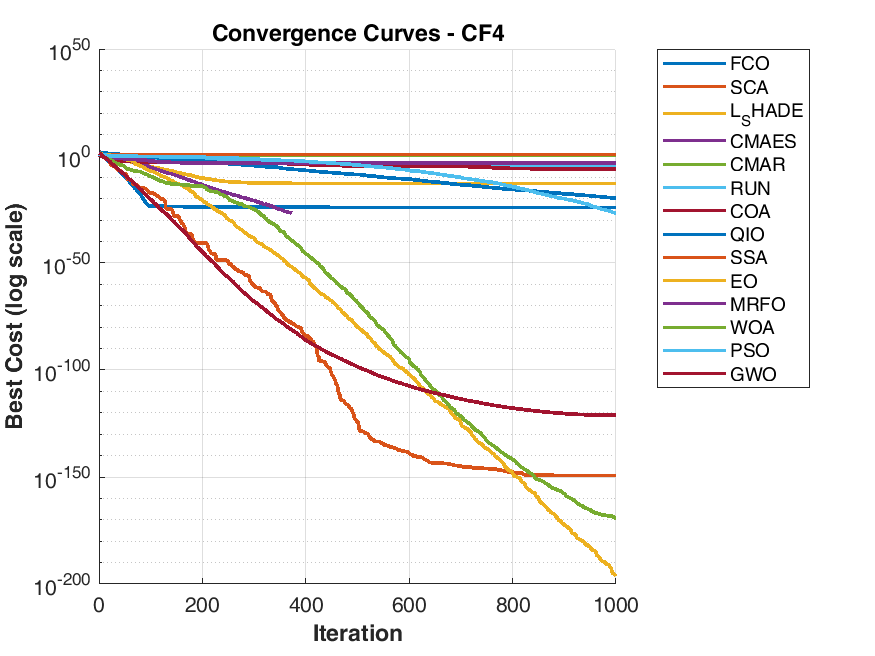

Supplement: S1 Data — (ZIP) [file pone.0341325.s004.zip › FCO Codes/CEC2005/Results/ConvergencePlots 1/CF4_all_algorithms.png]

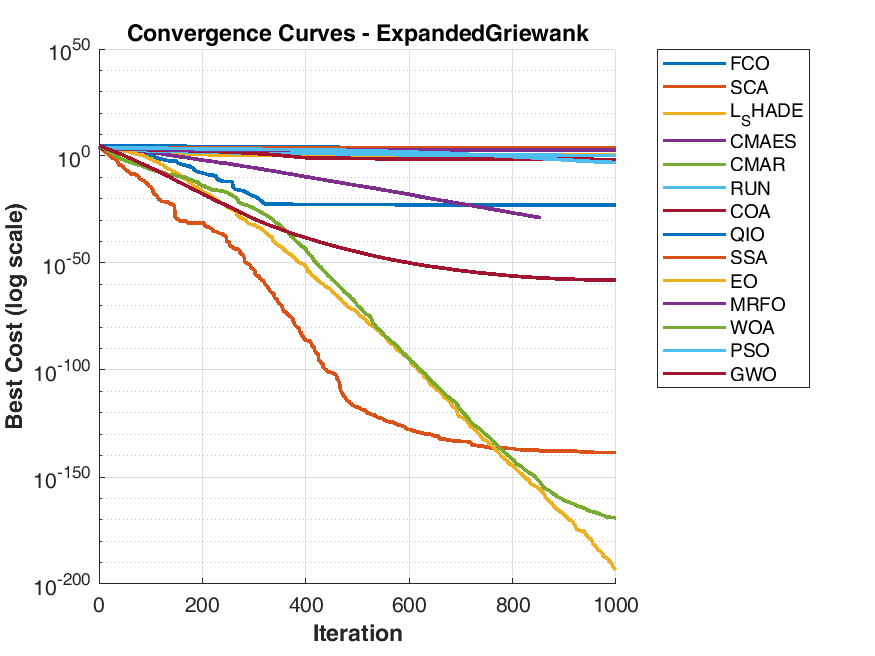

Supplement: S1 Data — (ZIP) [file pone.0341325.s004.zip › FCO Codes/CEC2005/Results/ConvergencePlots 1/ExpandedGriewank_all_algorithms.png]

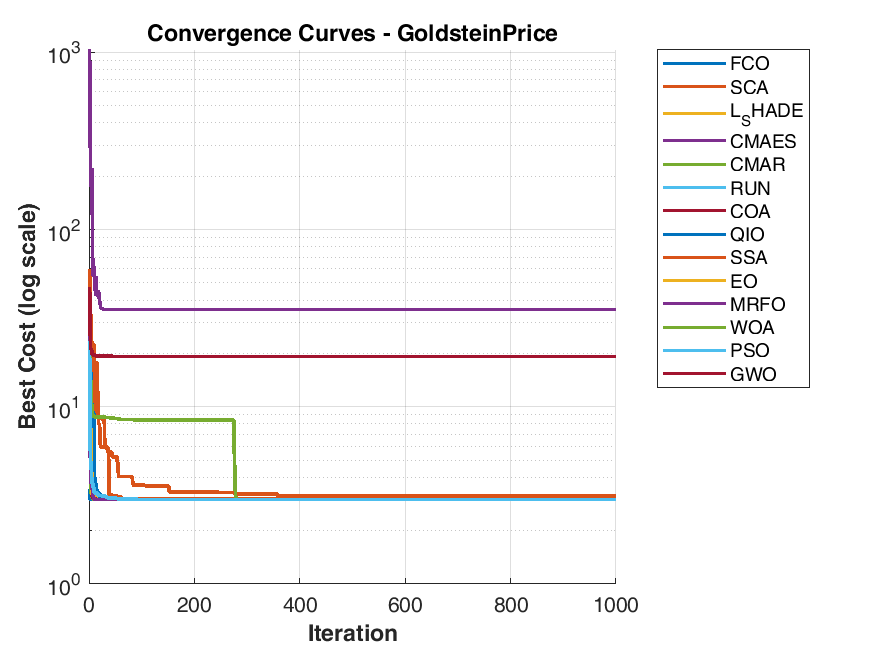

Supplement: S1 Data — (ZIP) [file pone.0341325.s004.zip › FCO Codes/CEC2005/Results/ConvergencePlots 1/GoldsteinPrice_all_algorithms.png]

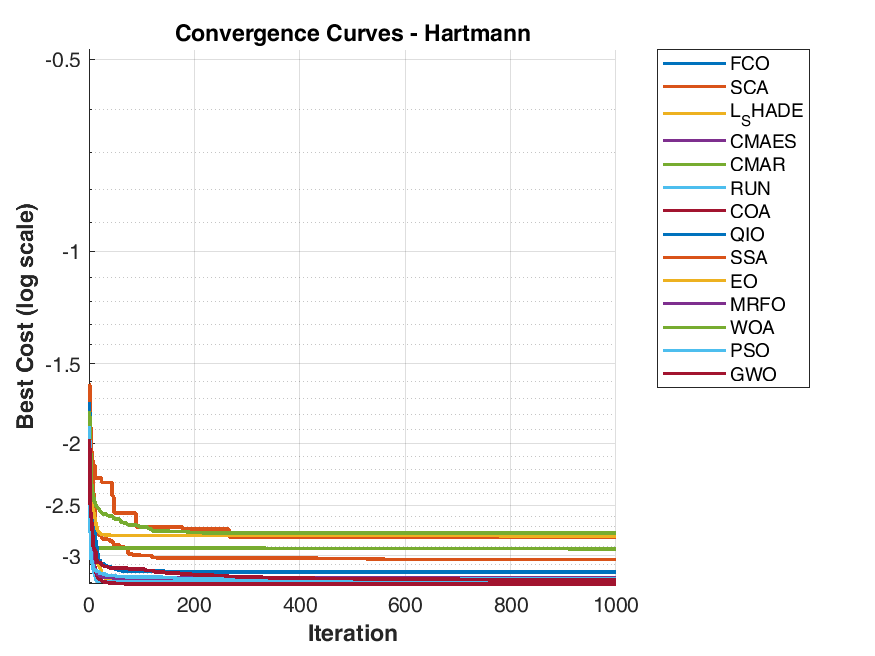

Supplement: S1 Data — (ZIP) [file pone.0341325.s004.zip › FCO Codes/CEC2005/Results/ConvergencePlots 1/Hartmann_all_algorithms.png]

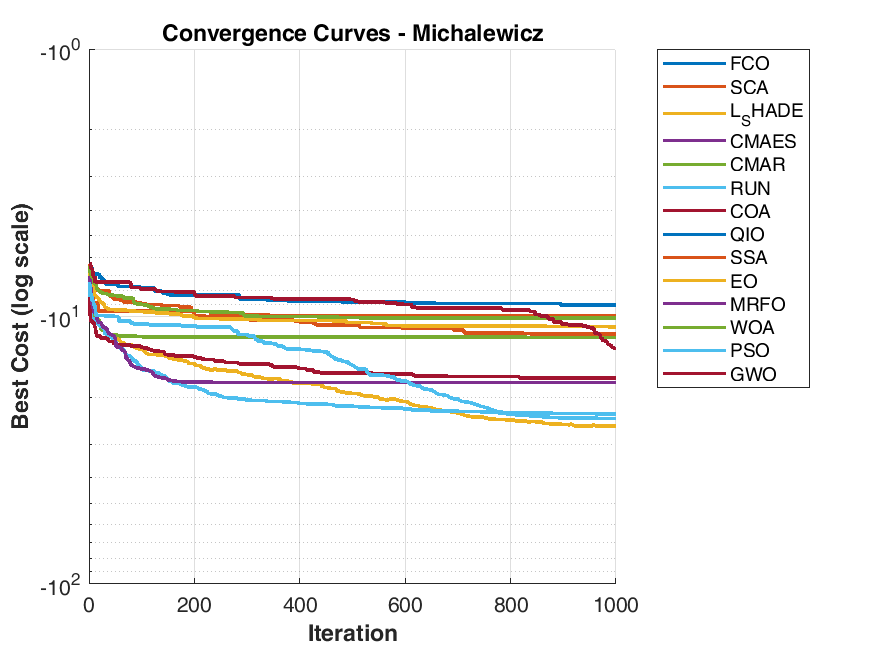

Supplement: S1 Data — (ZIP) [file pone.0341325.s004.zip › FCO Codes/CEC2005/Results/ConvergencePlots 1/Michalewicz_all_algorithms.png]

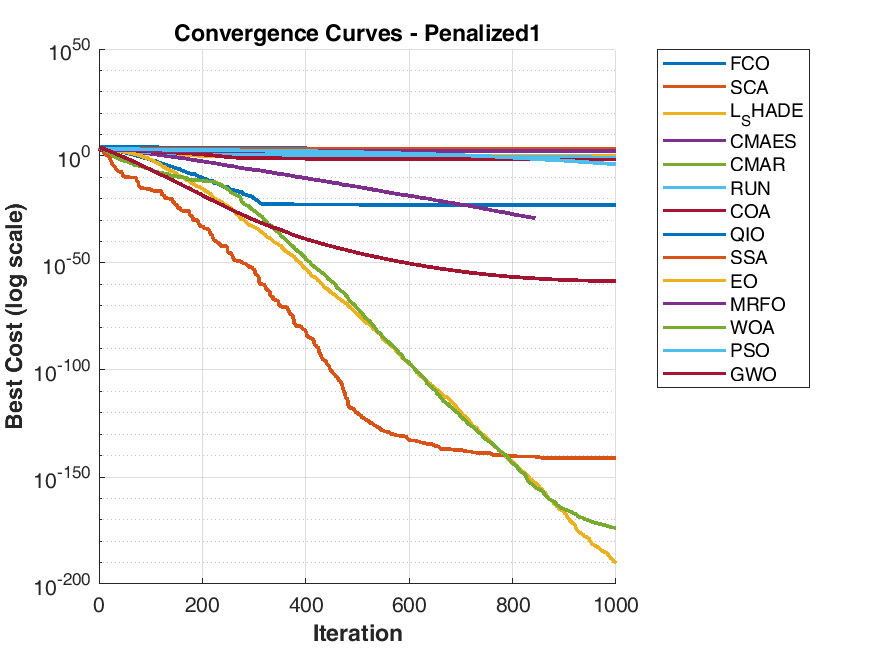

Supplement: S1 Data — (ZIP) [file pone.0341325.s004.zip › FCO Codes/CEC2005/Results/ConvergencePlots 1/Penalized1_all_algorithms.png]

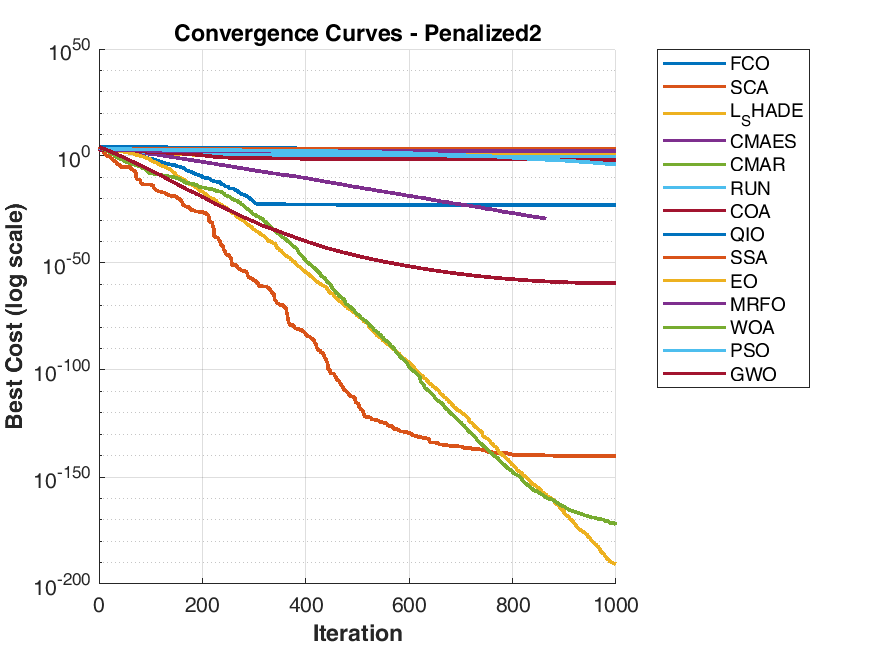

Supplement: S1 Data — (ZIP) [file pone.0341325.s004.zip › FCO Codes/CEC2005/Results/ConvergencePlots 1/Penalized2_all_algorithms.png]

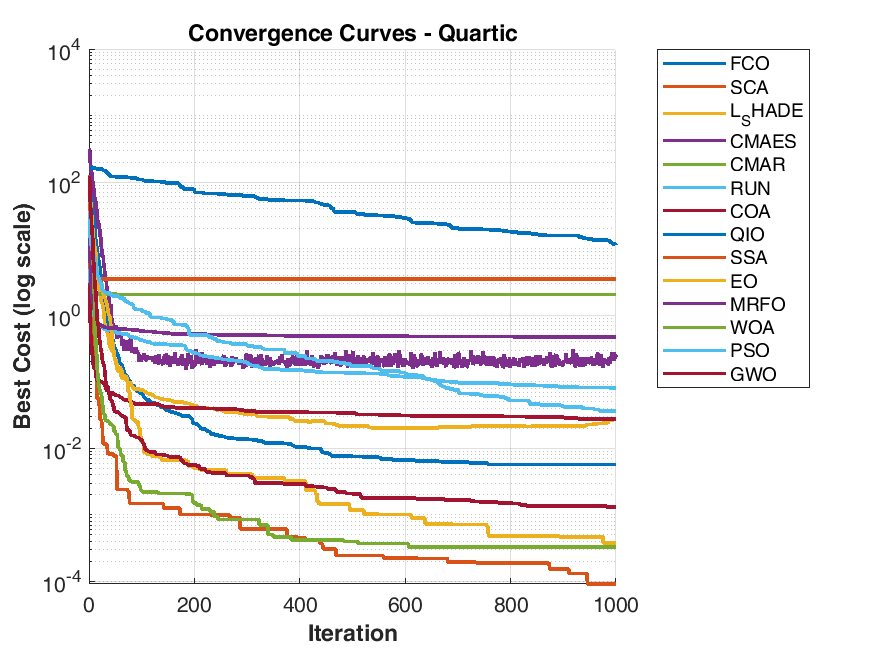

Supplement: S1 Data — (ZIP) [file pone.0341325.s004.zip › FCO Codes/CEC2005/Results/ConvergencePlots 1/Quartic_all_algorithms.png]

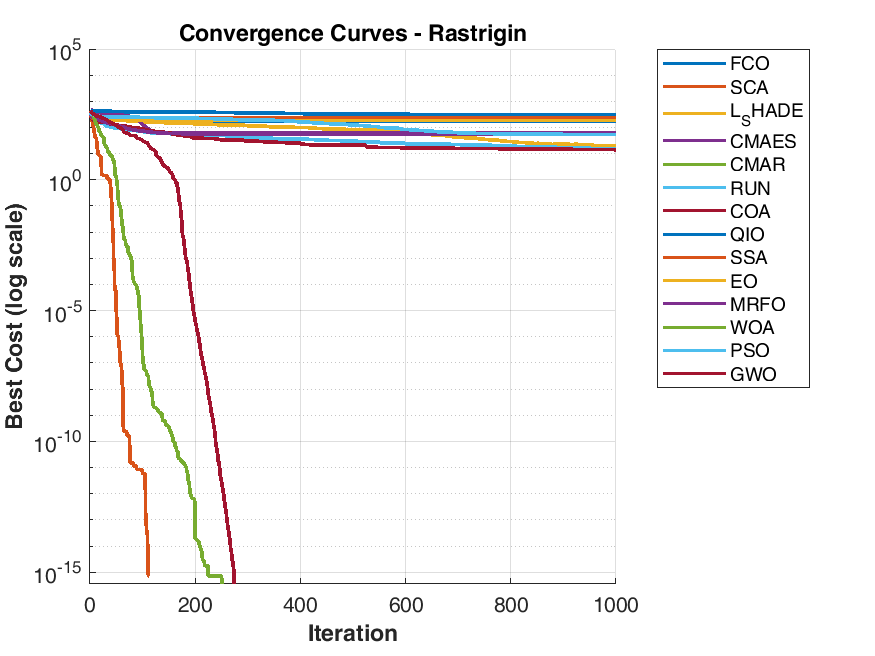

Supplement: S1 Data — (ZIP) [file pone.0341325.s004.zip › FCO Codes/CEC2005/Results/ConvergencePlots 1/Rastrigin_all_algorithms.png]

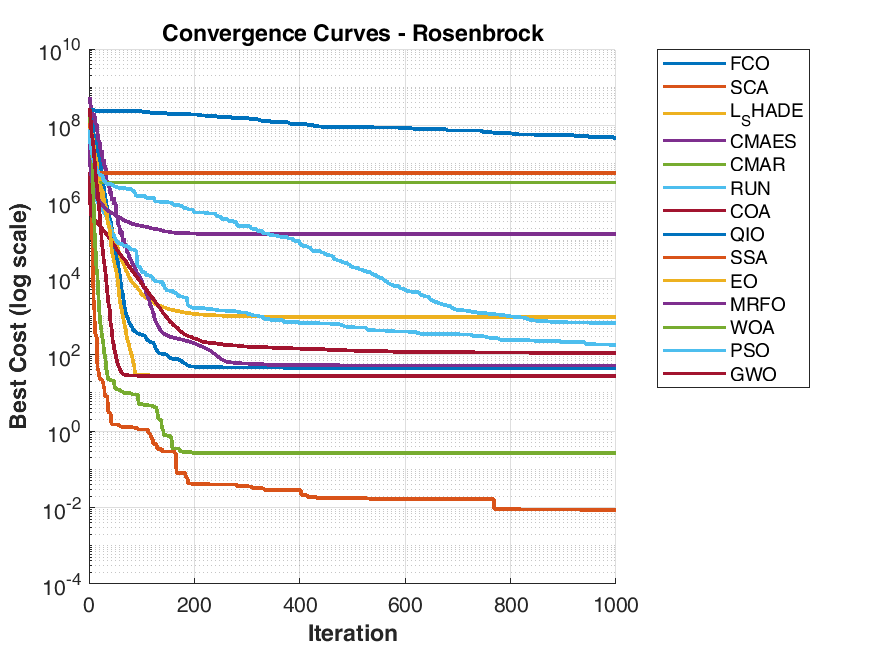

Supplement: S1 Data — (ZIP) [file pone.0341325.s004.zip › FCO Codes/CEC2005/Results/ConvergencePlots 1/Rosenbrock_all_algorithms.png]

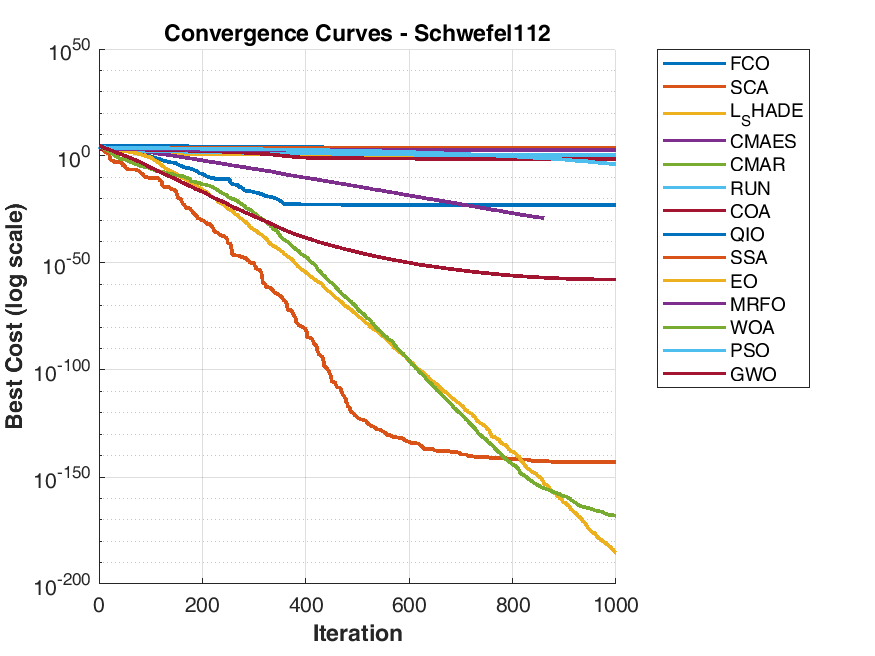

Supplement: S1 Data — (ZIP) [file pone.0341325.s004.zip › FCO Codes/CEC2005/Results/ConvergencePlots 1/Schwefel112_all_algorithms.png]

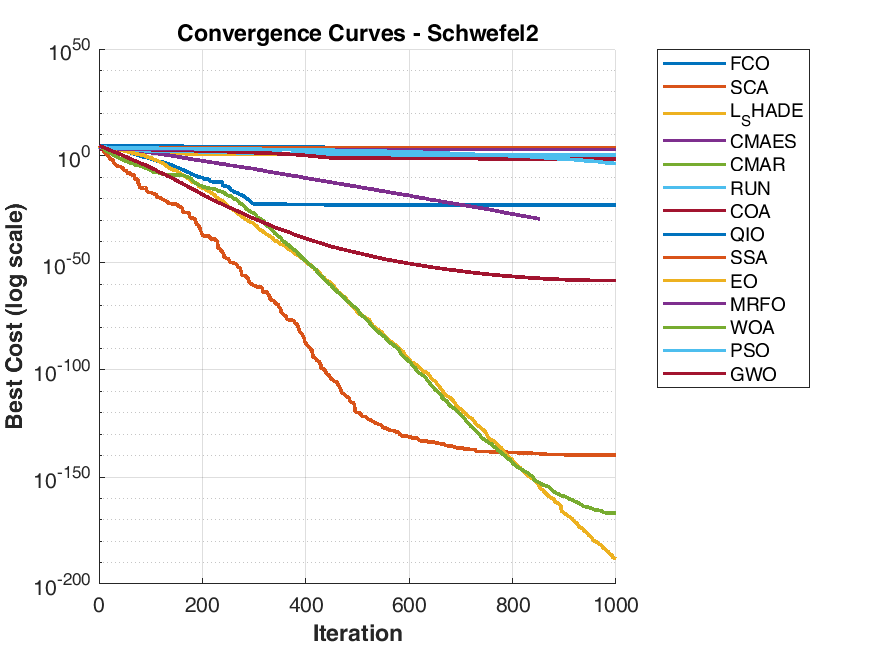

Supplement: S1 Data — (ZIP) [file pone.0341325.s004.zip › FCO Codes/CEC2005/Results/ConvergencePlots 1/Schwefel2_all_algorithms.png]

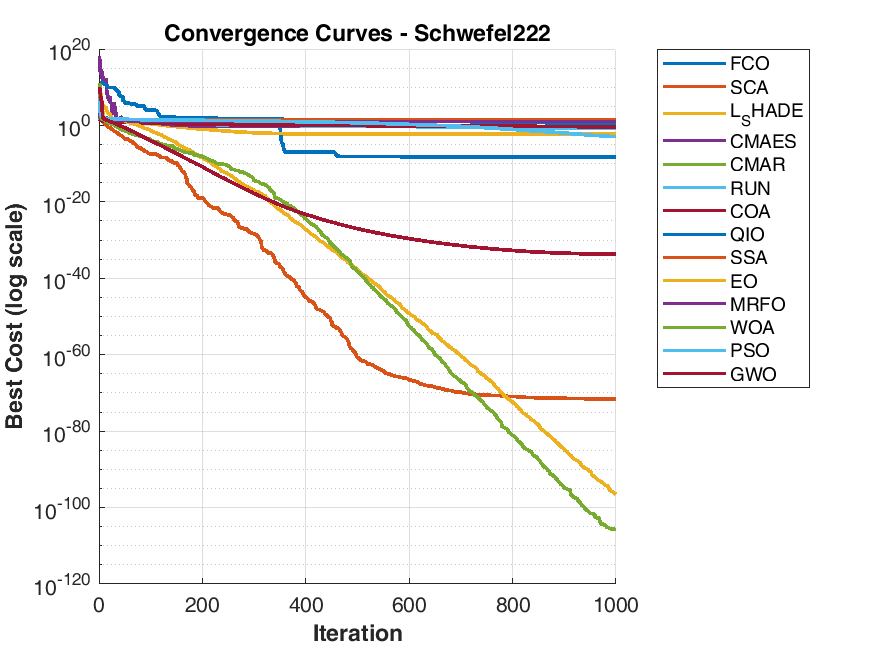

Supplement: S1 Data — (ZIP) [file pone.0341325.s004.zip › FCO Codes/CEC2005/Results/ConvergencePlots 1/Schwefel222_all_algorithms.png]

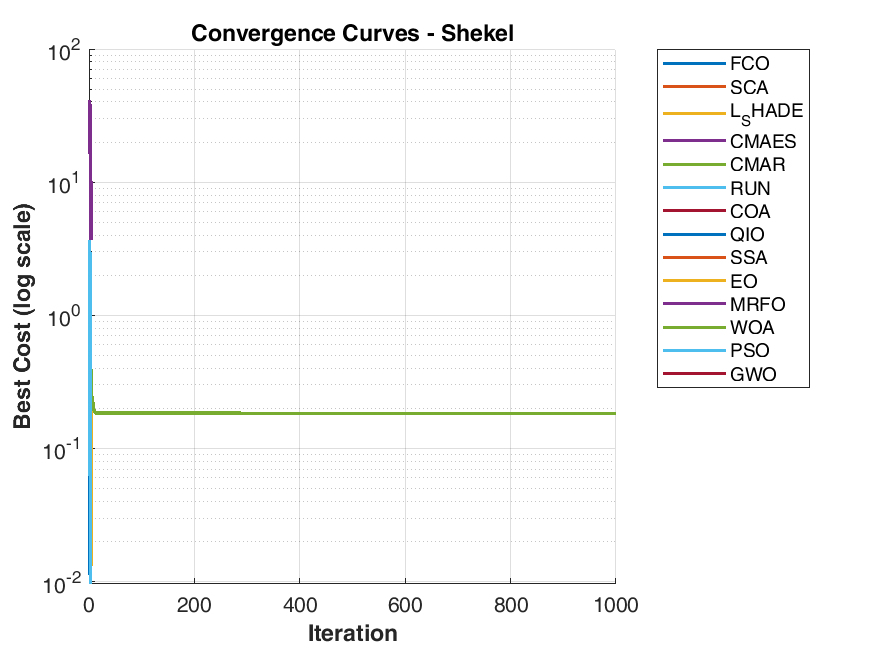

Supplement: S1 Data — (ZIP) [file pone.0341325.s004.zip › FCO Codes/CEC2005/Results/ConvergencePlots 1/Shekel_all_algorithms.png]

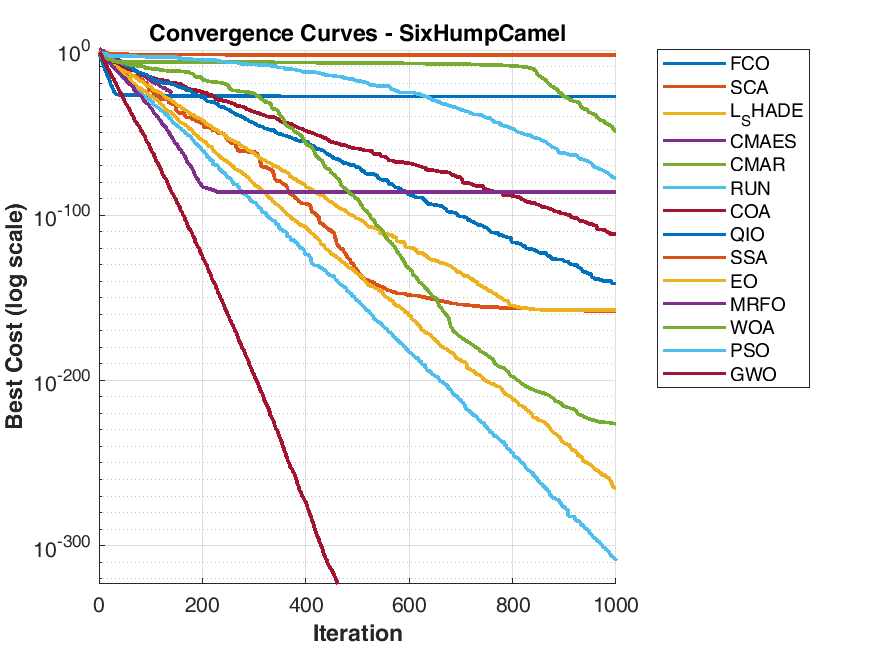

Supplement: S1 Data — (ZIP) [file pone.0341325.s004.zip › FCO Codes/CEC2005/Results/ConvergencePlots 1/SixHumpCamel_all_algorithms.png]

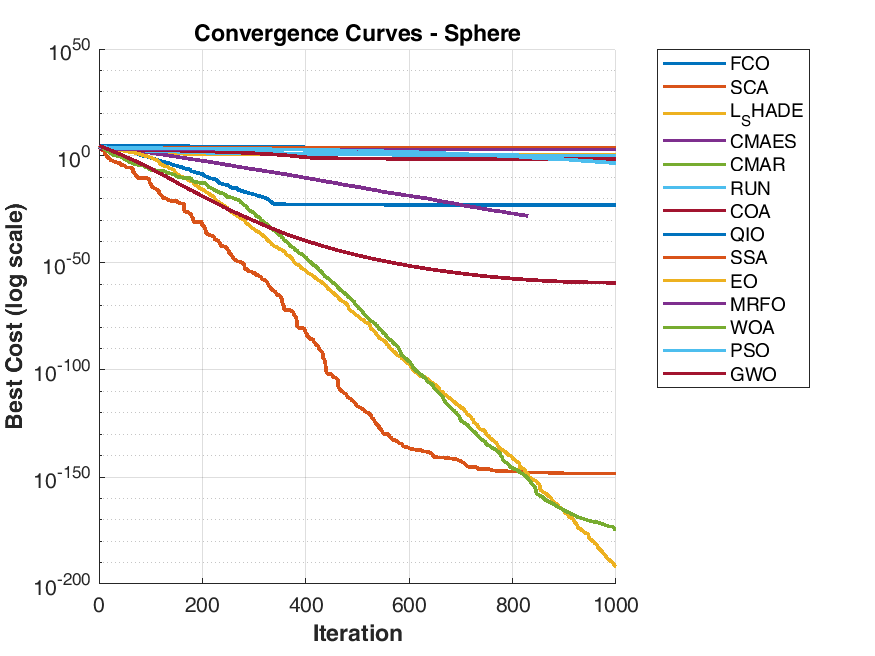

Supplement: S1 Data — (ZIP) [file pone.0341325.s004.zip › FCO Codes/CEC2005/Results/ConvergencePlots 1/Sphere_all_algorithms.png]

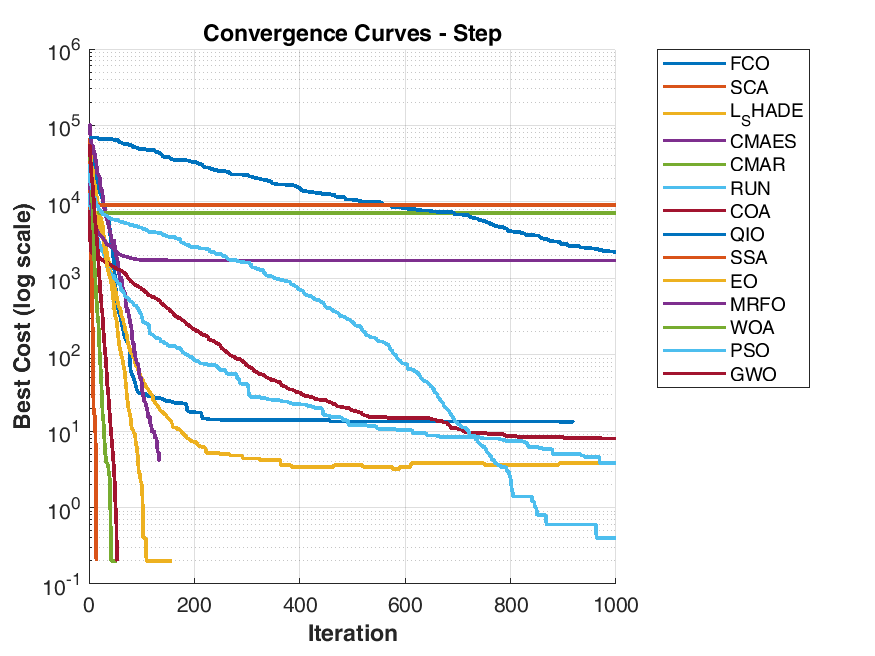

Supplement: S1 Data — (ZIP) [file pone.0341325.s004.zip › FCO Codes/CEC2005/Results/ConvergencePlots 1/Step_all_algorithms.png]

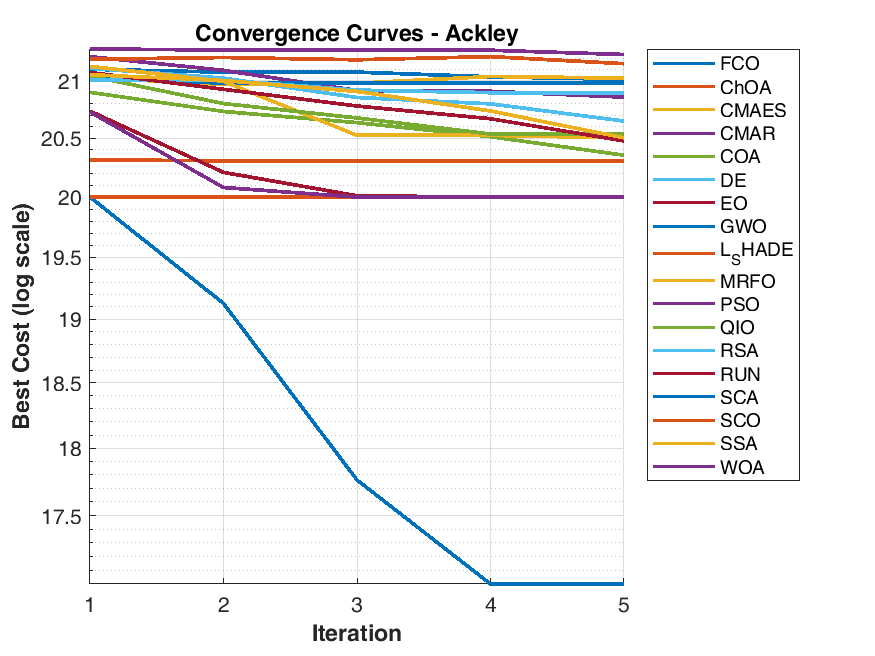

Supplement: S1 Data — (ZIP) [file pone.0341325.s004.zip › FCO Codes/CEC2005/Results/ConvergencePlots/Ackley_all_algorithms.png]

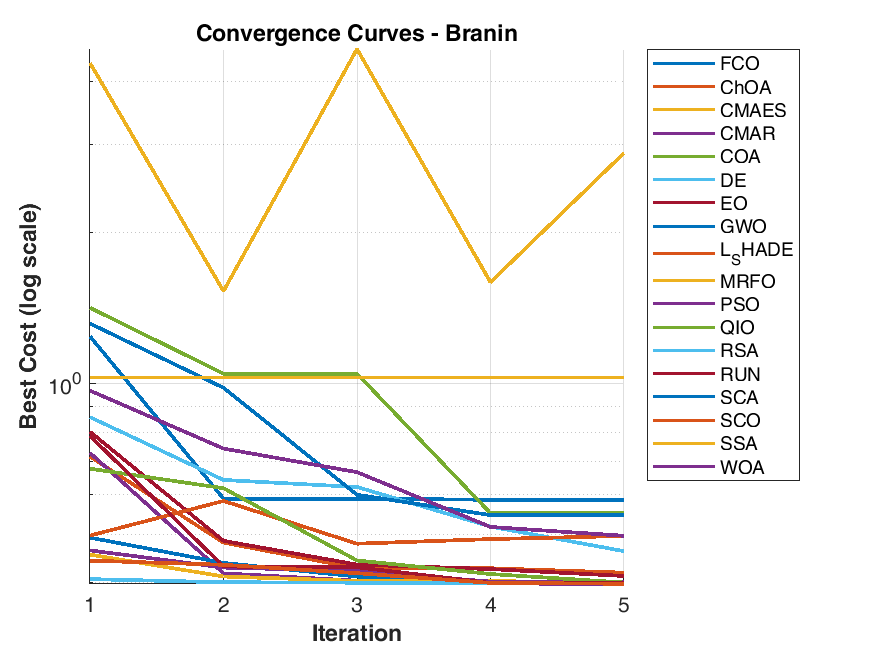

Supplement: S1 Data — (ZIP) [file pone.0341325.s004.zip › FCO Codes/CEC2005/Results/ConvergencePlots/Branin_all_algorithms.png]

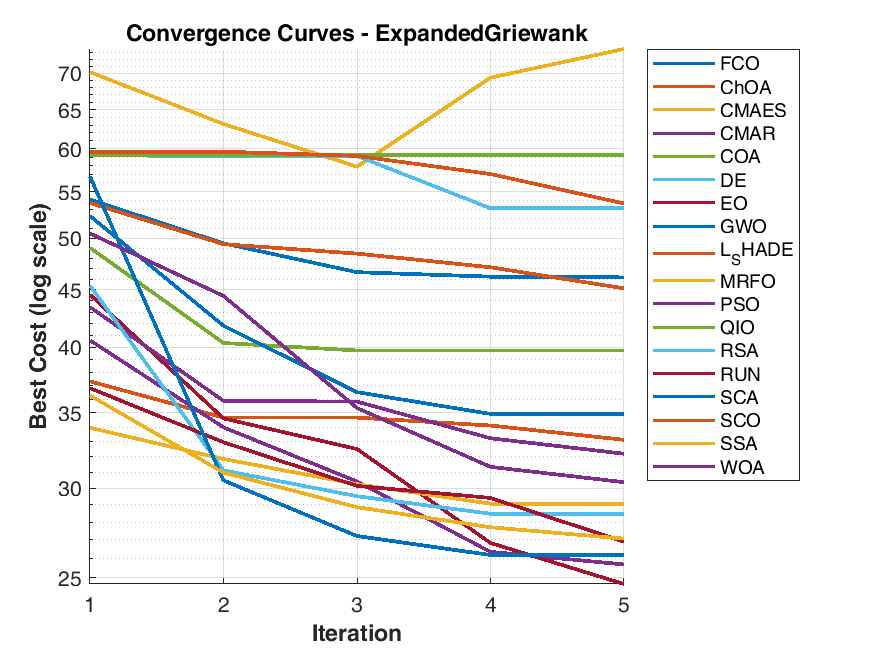

Supplement: S1 Data — (ZIP) [file pone.0341325.s004.zip › FCO Codes/CEC2005/Results/ConvergencePlots/ExpandedGriewank_all_algorithms.png]

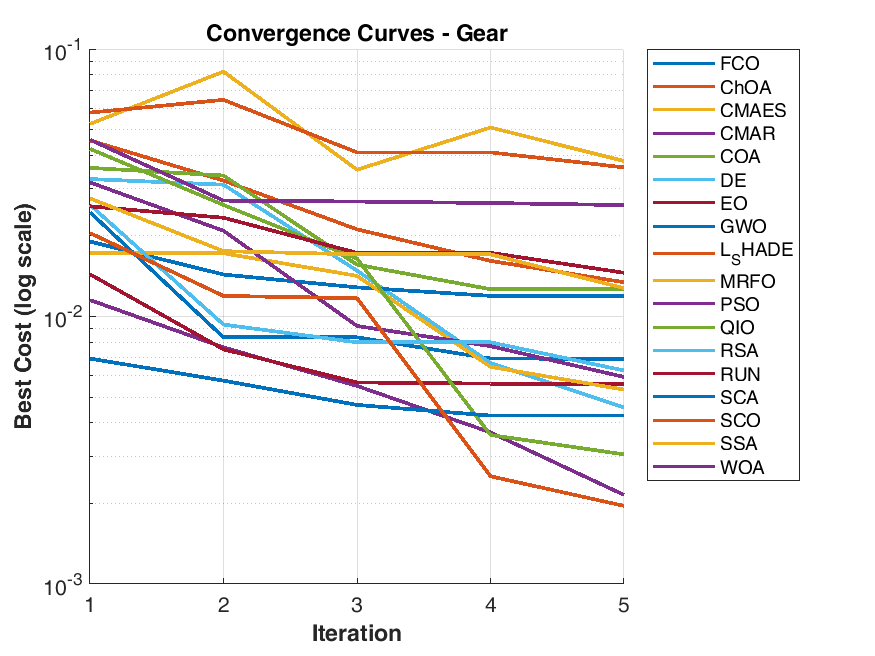

Supplement: S1 Data — (ZIP) [file pone.0341325.s004.zip › FCO Codes/CEC2005/Results/ConvergencePlots/Gear_all_algorithms.png]

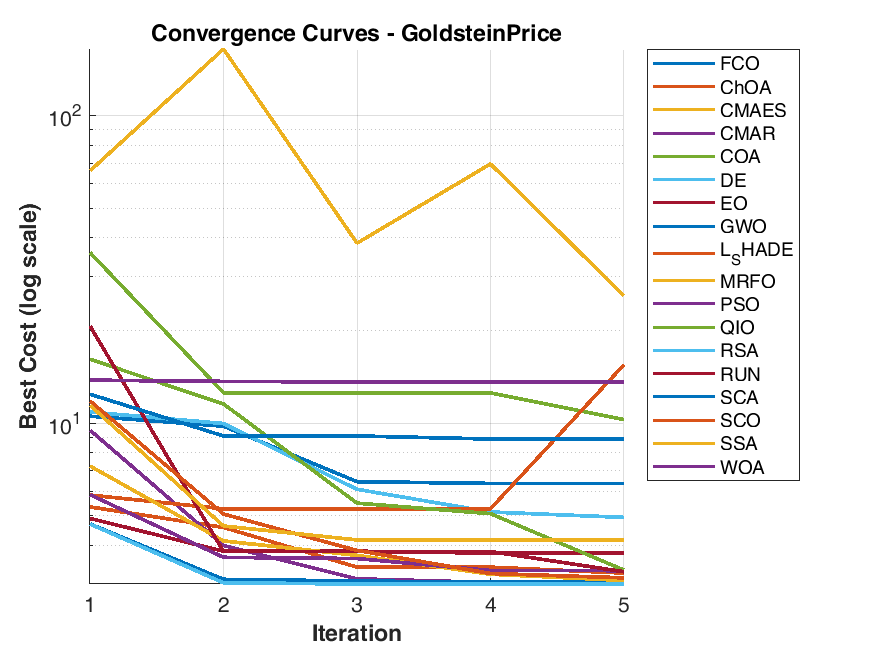

Supplement: S1 Data — (ZIP) [file pone.0341325.s004.zip › FCO Codes/CEC2005/Results/ConvergencePlots/GoldsteinPrice_all_algorithms.png]

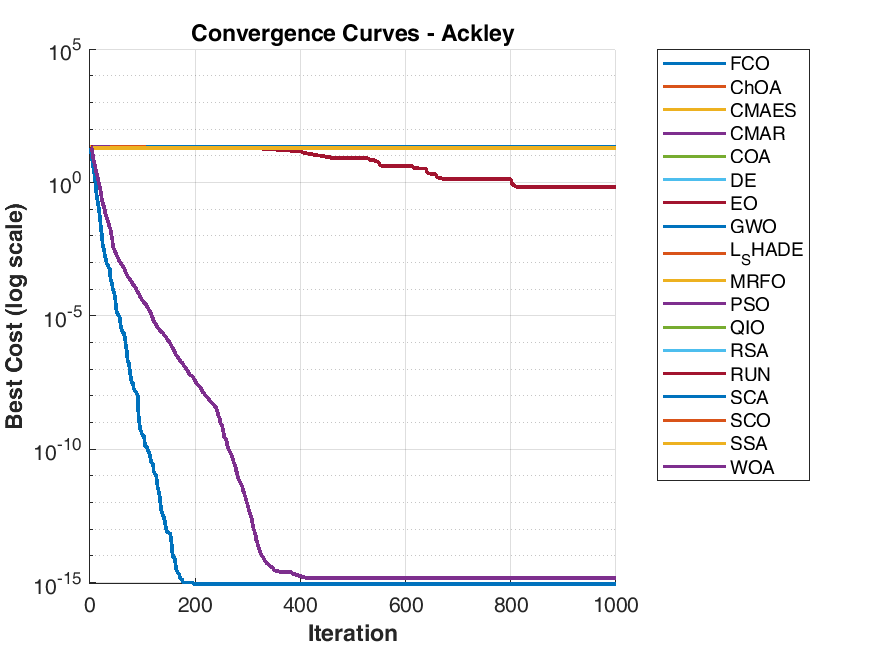

Supplement: S1 Data — (ZIP) [file pone.0341325.s004.zip › FCO Codes/CEC2005/Results/ConvergencePlots/New folder/Ackley_all_algorithms.png]

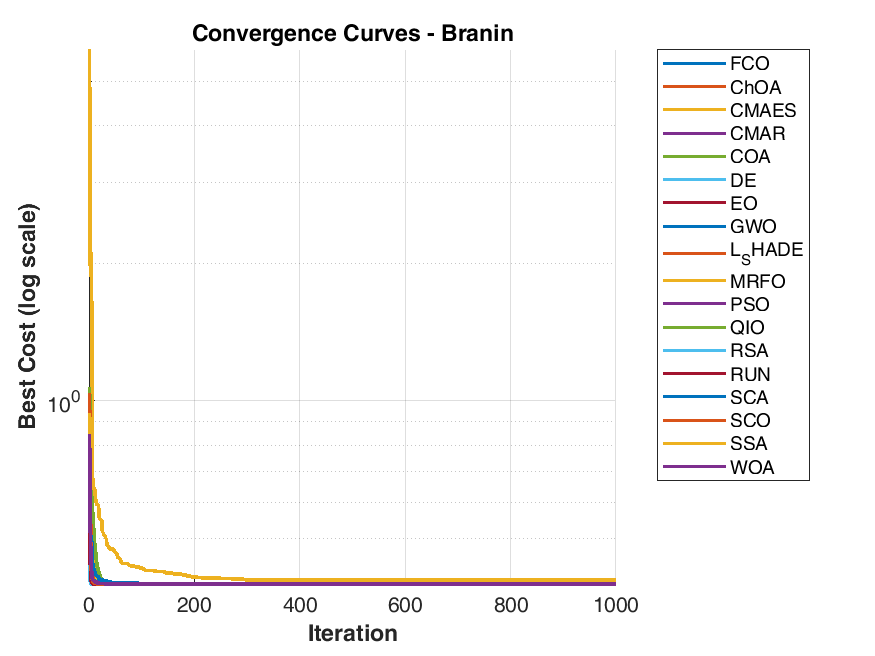

Supplement: S1 Data — (ZIP) [file pone.0341325.s004.zip › FCO Codes/CEC2005/Results/ConvergencePlots/New folder/Branin_all_algorithms.png]

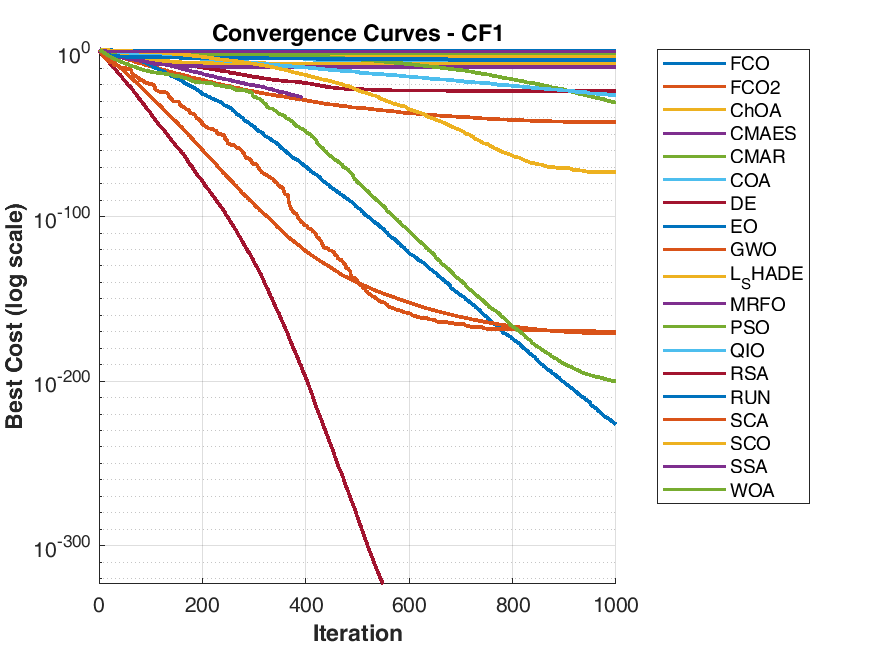

Supplement: S1 Data — (ZIP) [file pone.0341325.s004.zip › FCO Codes/CEC2005/Results/ConvergencePlots/New folder/CF1_all_algorithms.png]

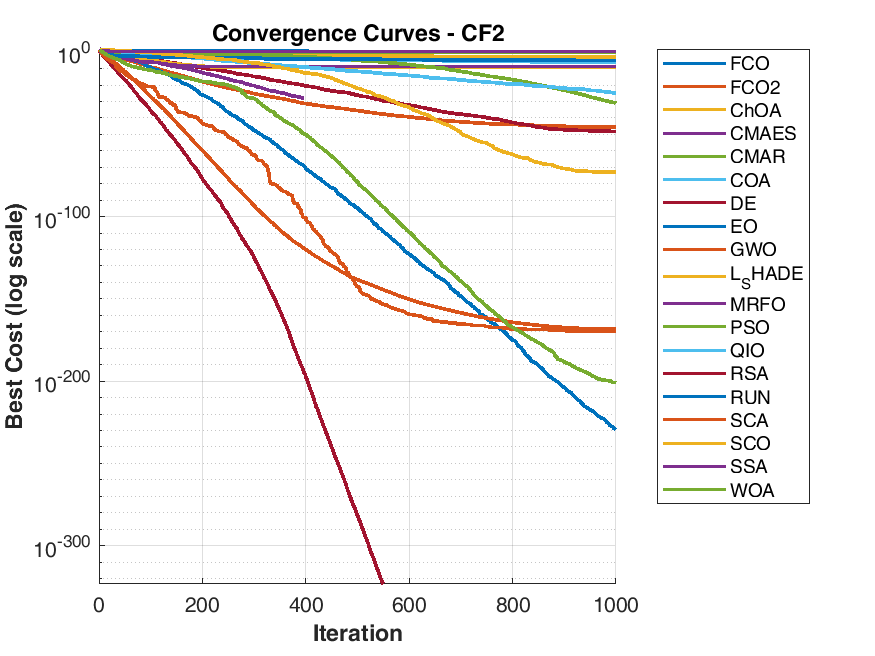

Supplement: S1 Data — (ZIP) [file pone.0341325.s004.zip › FCO Codes/CEC2005/Results/ConvergencePlots/New folder/CF2_all_algorithms.png]

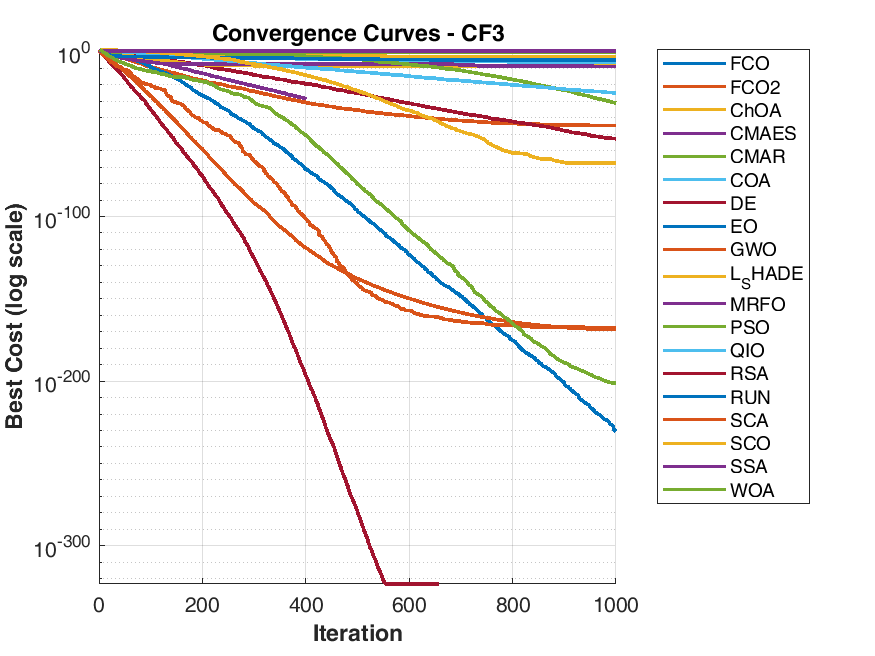

Supplement: S1 Data — (ZIP) [file pone.0341325.s004.zip › FCO Codes/CEC2005/Results/ConvergencePlots/New folder/CF3_all_algorithms.png]

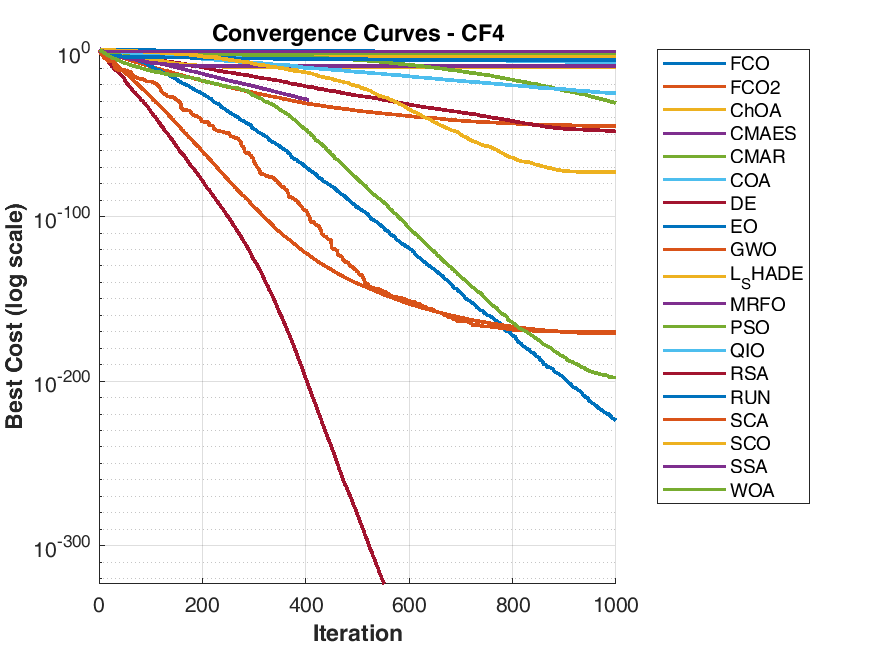

Supplement: S1 Data — (ZIP) [file pone.0341325.s004.zip › FCO Codes/CEC2005/Results/ConvergencePlots/New folder/CF4_all_algorithms.png]

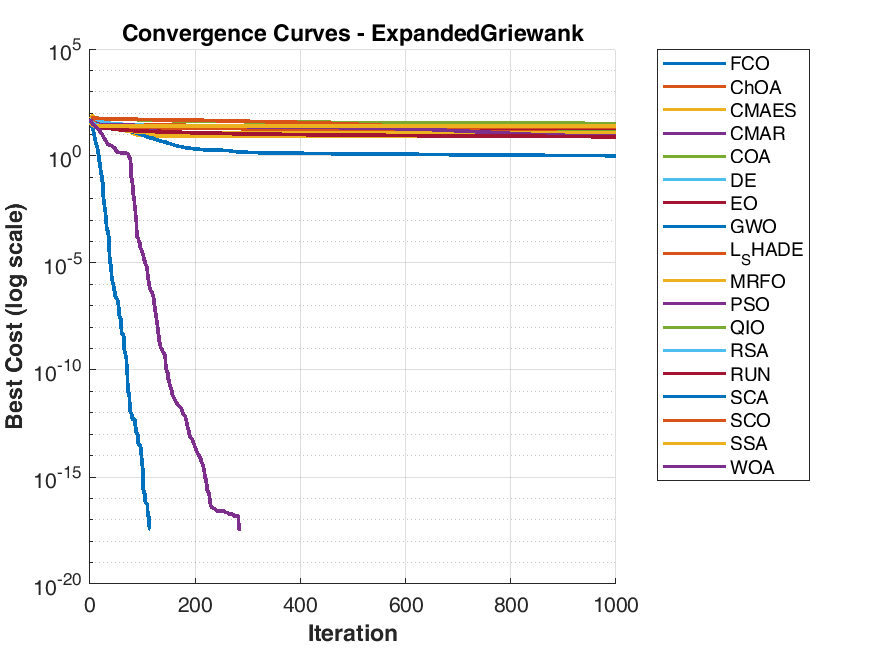

Supplement: S1 Data — (ZIP) [file pone.0341325.s004.zip › FCO Codes/CEC2005/Results/ConvergencePlots/New folder/ExpandedGriewank_all_algorithms.png]

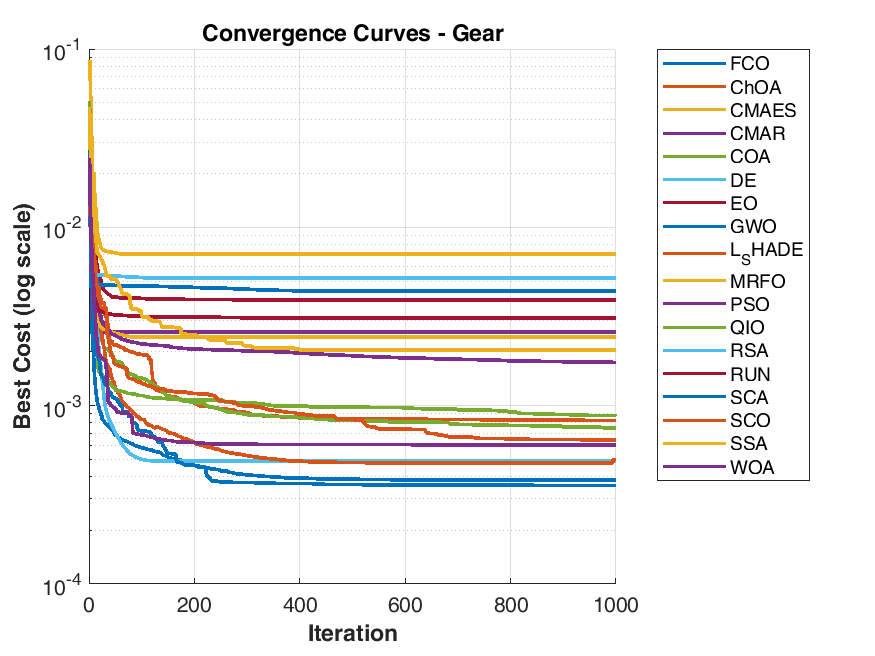

Supplement: S1 Data — (ZIP) [file pone.0341325.s004.zip › FCO Codes/CEC2005/Results/ConvergencePlots/New folder/Gear_all_algorithms.png]

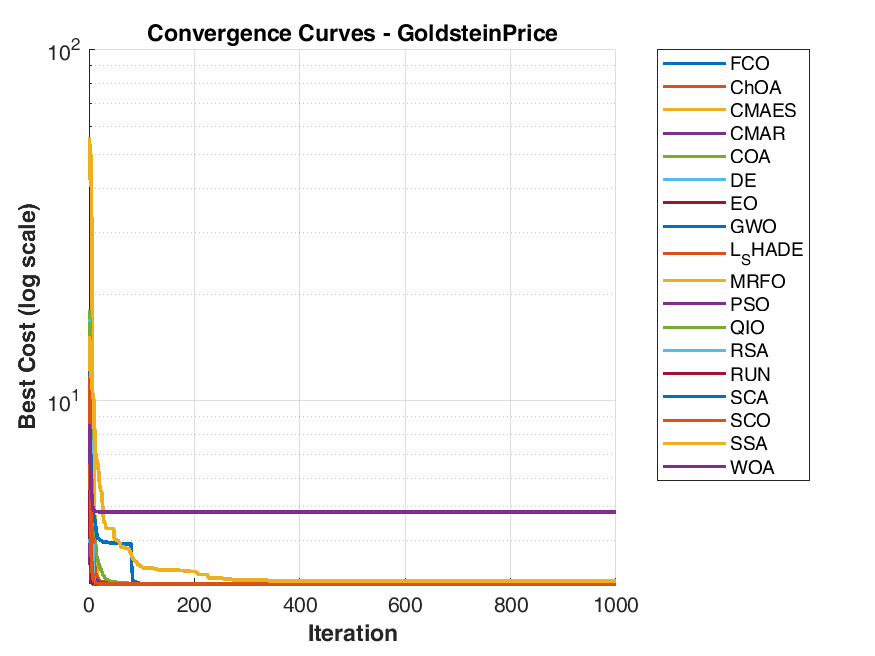

Supplement: S1 Data — (ZIP) [file pone.0341325.s004.zip › FCO Codes/CEC2005/Results/ConvergencePlots/New folder/GoldsteinPrice_all_algorithms.png]

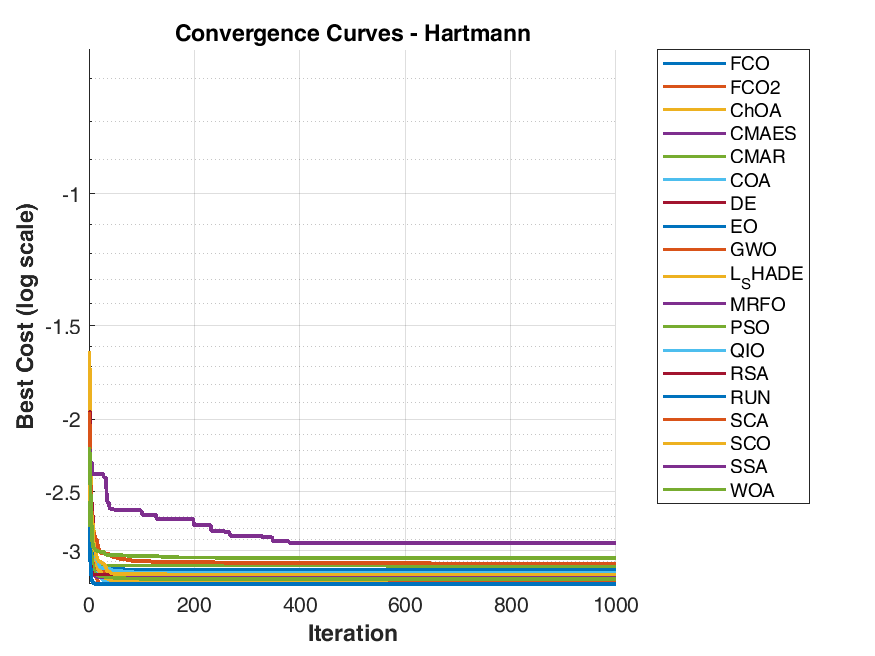

Supplement: S1 Data — (ZIP) [file pone.0341325.s004.zip › FCO Codes/CEC2005/Results/ConvergencePlots/New folder/Hartmann_all_algorithms.png]

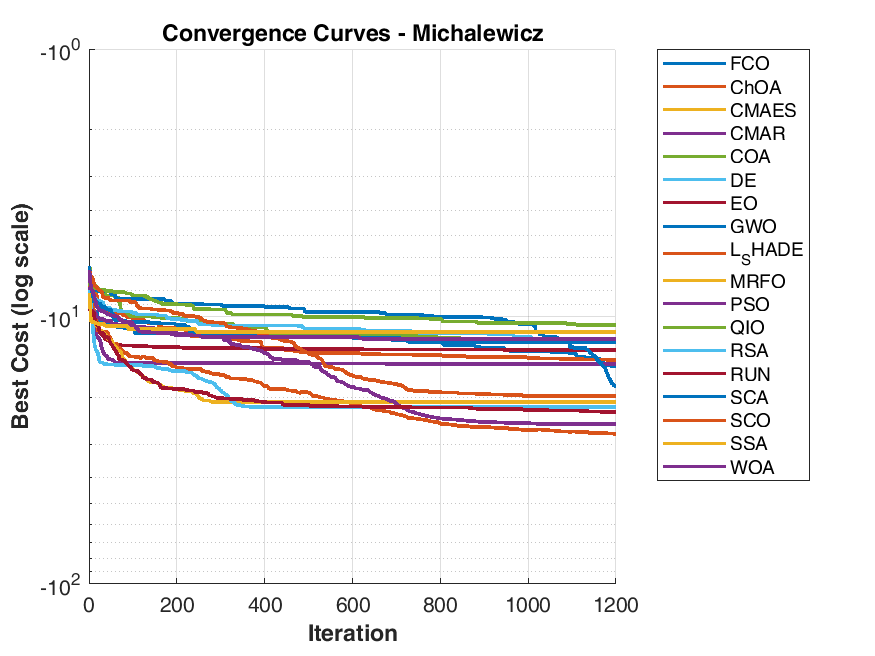

Supplement: S1 Data — (ZIP) [file pone.0341325.s004.zip › FCO Codes/CEC2005/Results/ConvergencePlots/New folder/Michalewicz_all_algorithms.png]

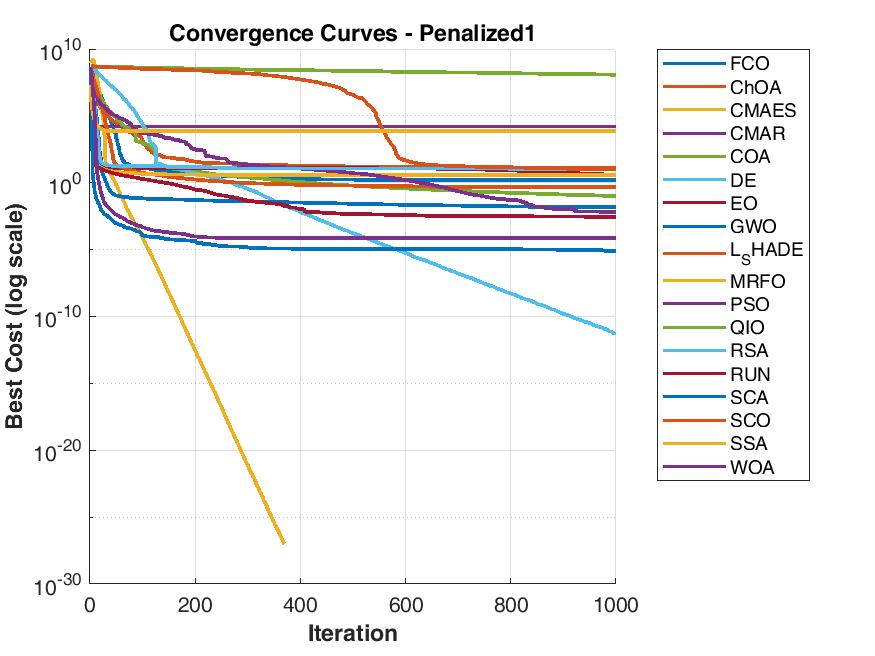

Supplement: S1 Data — (ZIP) [file pone.0341325.s004.zip › FCO Codes/CEC2005/Results/ConvergencePlots/New folder/Penalized1_all_algorithms.png]

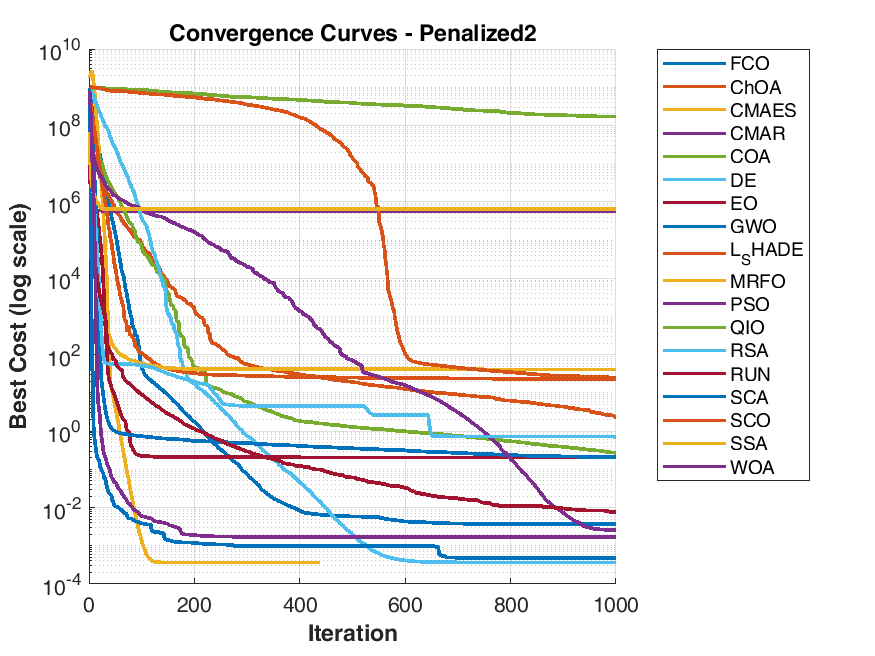

Supplement: S1 Data — (ZIP) [file pone.0341325.s004.zip › FCO Codes/CEC2005/Results/ConvergencePlots/New folder/Penalized2_all_algorithms.png]

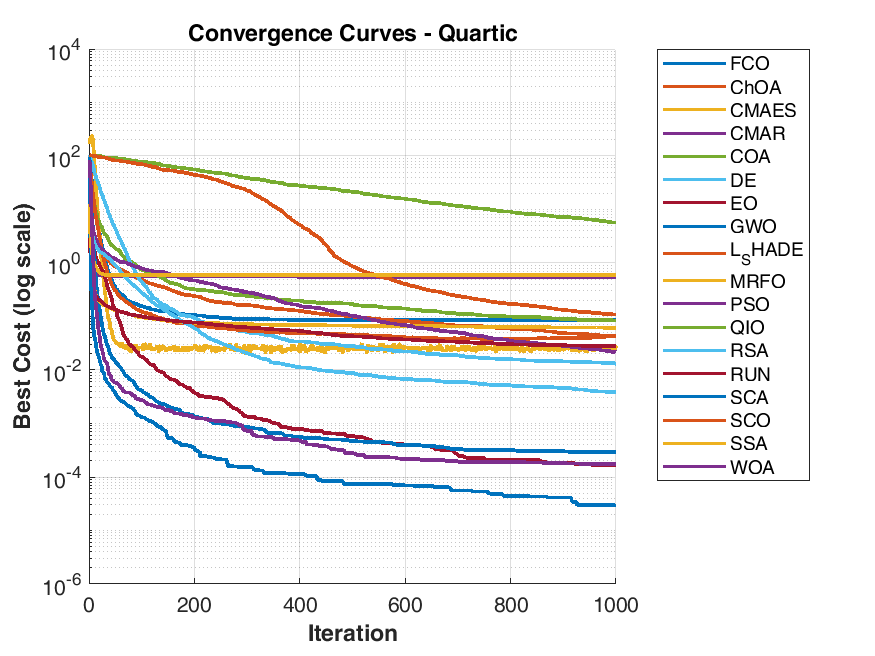

Supplement: S1 Data — (ZIP) [file pone.0341325.s004.zip › FCO Codes/CEC2005/Results/ConvergencePlots/New folder/Quartic_all_algorithms.png]

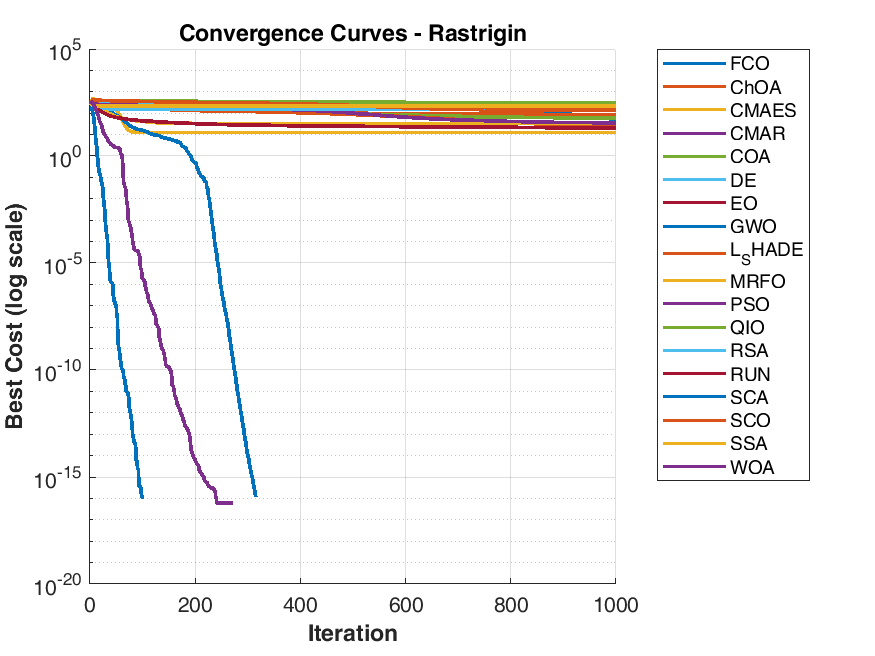

Supplement: S1 Data — (ZIP) [file pone.0341325.s004.zip › FCO Codes/CEC2005/Results/ConvergencePlots/New folder/Rastrigin_all_algorithms.png]

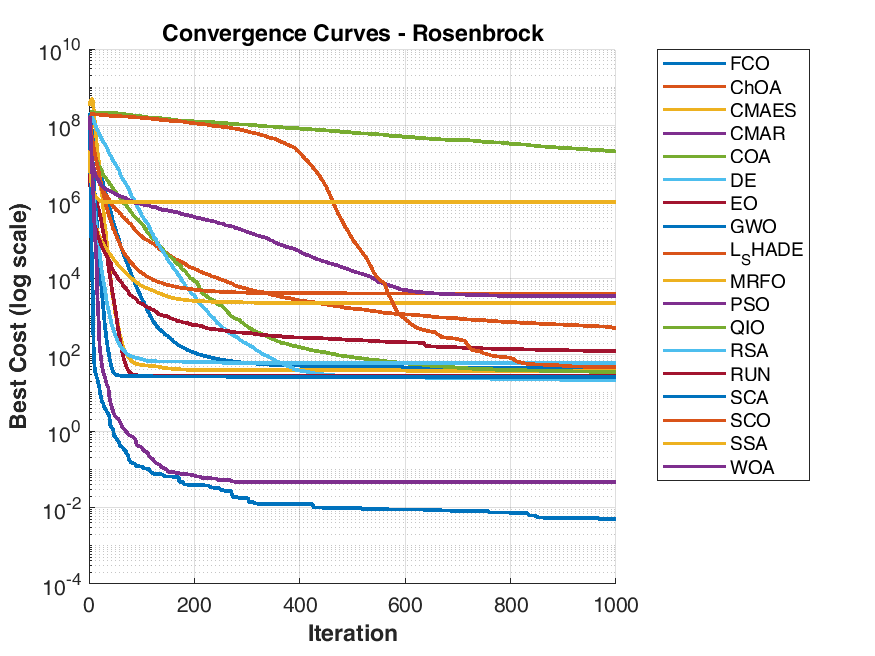

Supplement: S1 Data — (ZIP) [file pone.0341325.s004.zip › FCO Codes/CEC2005/Results/ConvergencePlots/New folder/Rosenbrock_all_algorithms.png]

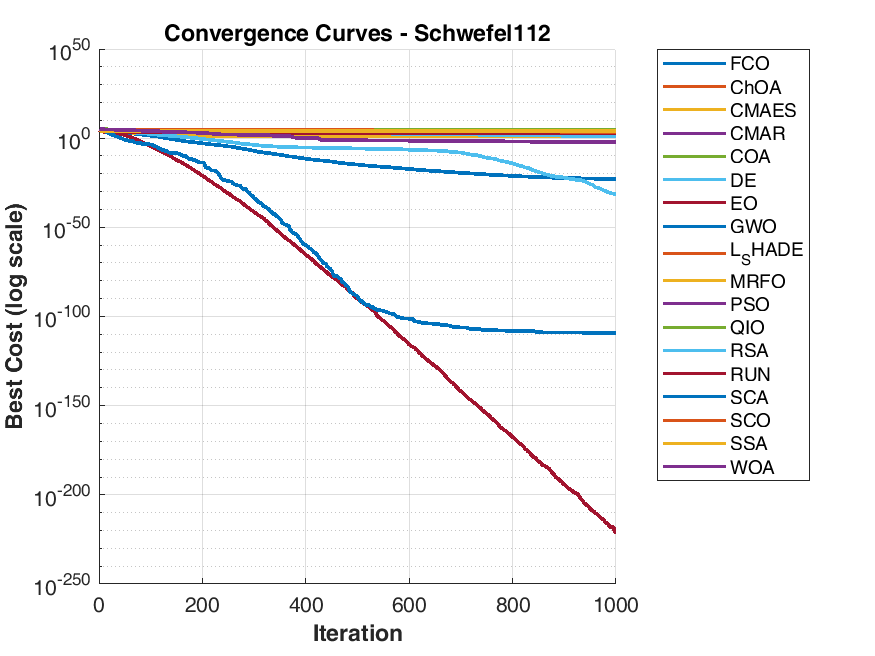

Supplement: S1 Data — (ZIP) [file pone.0341325.s004.zip › FCO Codes/CEC2005/Results/ConvergencePlots/New folder/Schwefel112_all_algorithms.png]

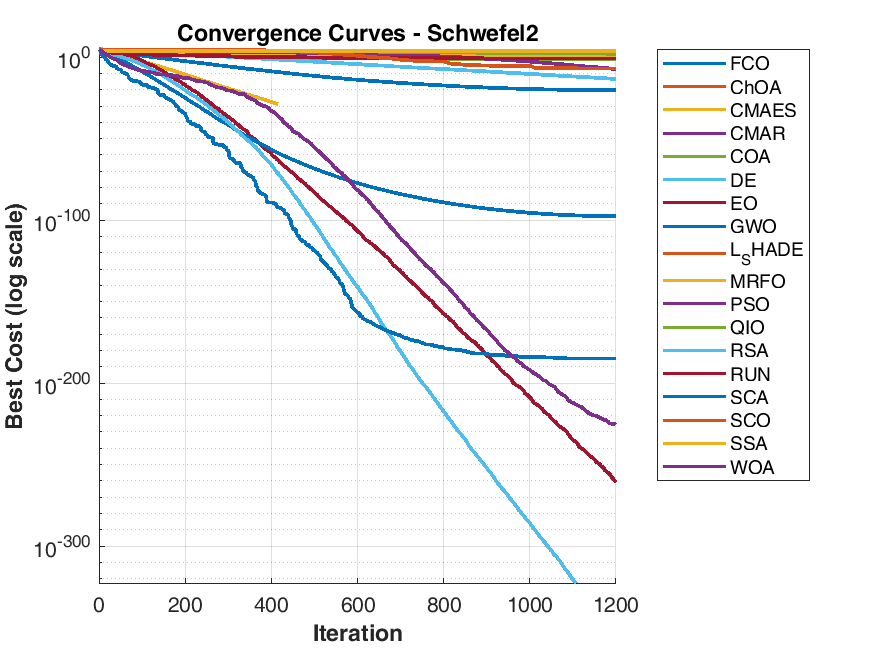

Supplement: S1 Data — (ZIP) [file pone.0341325.s004.zip › FCO Codes/CEC2005/Results/ConvergencePlots/New folder/Schwefel2_all_algorithms.png]

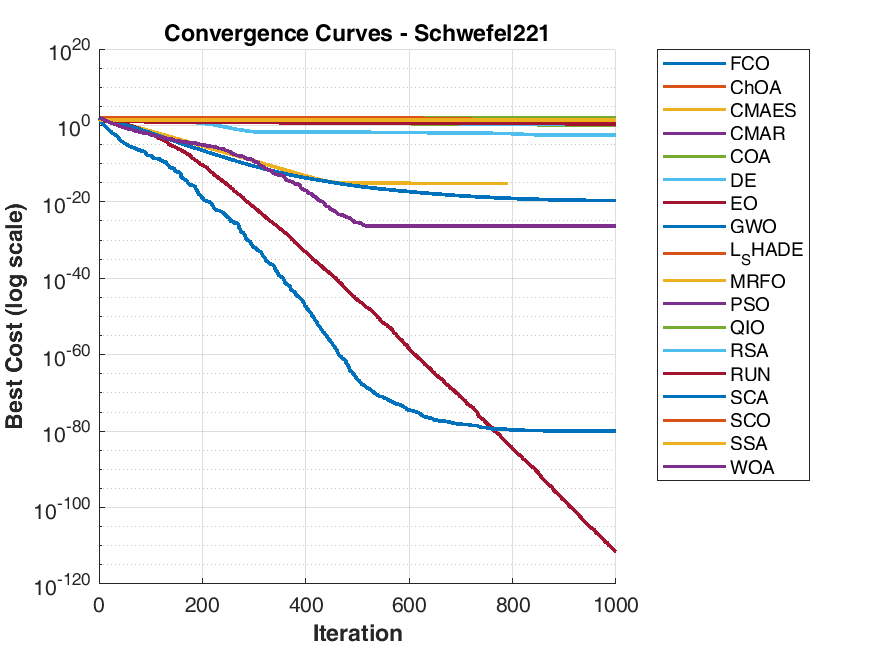

Supplement: S1 Data — (ZIP) [file pone.0341325.s004.zip › FCO Codes/CEC2005/Results/ConvergencePlots/New folder/Schwefel221_all_algorithms.png]

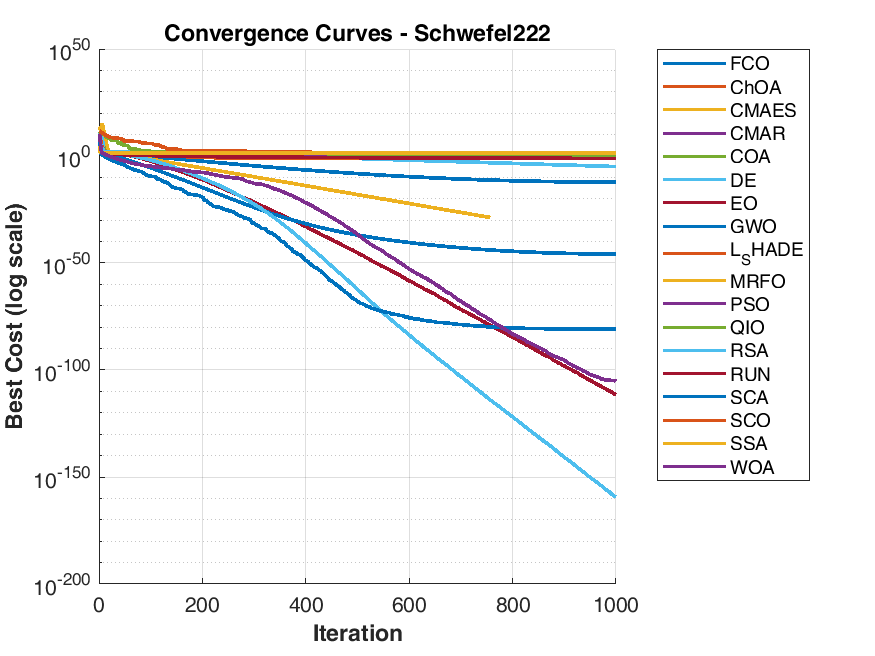

Supplement: S1 Data — (ZIP) [file pone.0341325.s004.zip › FCO Codes/CEC2005/Results/ConvergencePlots/New folder/Schwefel222_all_algorithms.png]

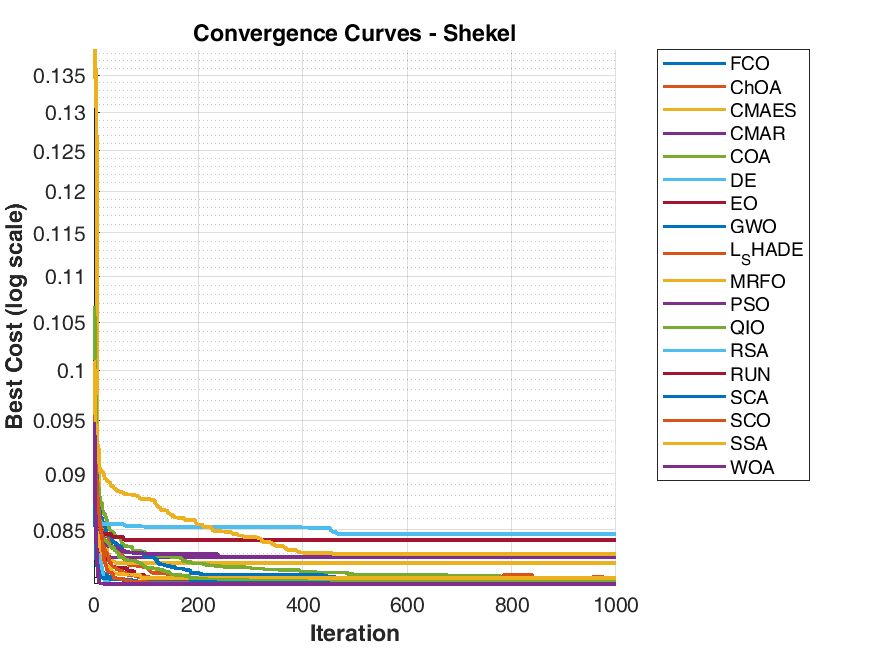

Supplement: S1 Data — (ZIP) [file pone.0341325.s004.zip › FCO Codes/CEC2005/Results/ConvergencePlots/New folder/Shekel_all_algorithms.png]

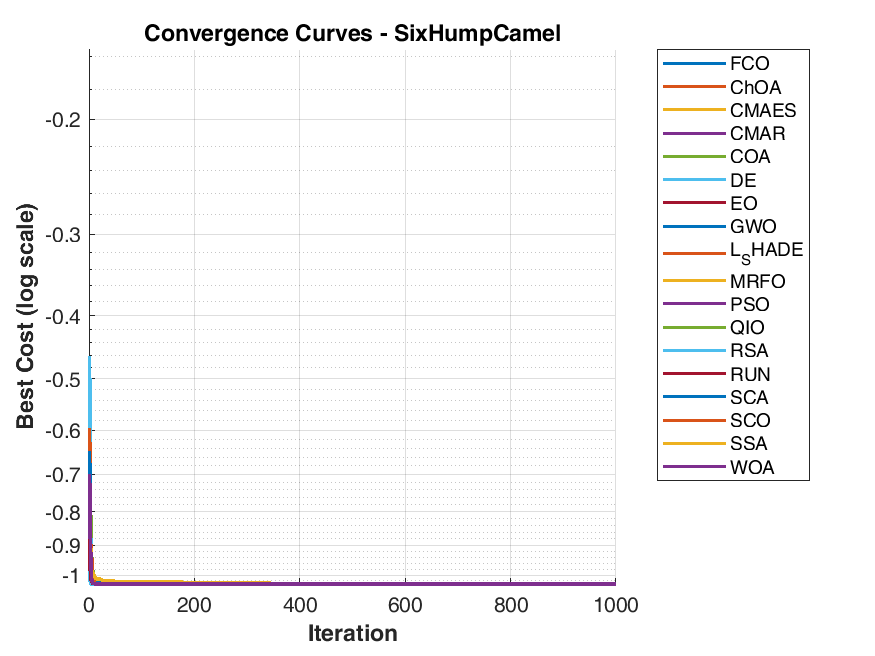

Supplement: S1 Data — (ZIP) [file pone.0341325.s004.zip › FCO Codes/CEC2005/Results/ConvergencePlots/New folder/SixHumpCamel_all_algorithms.png]

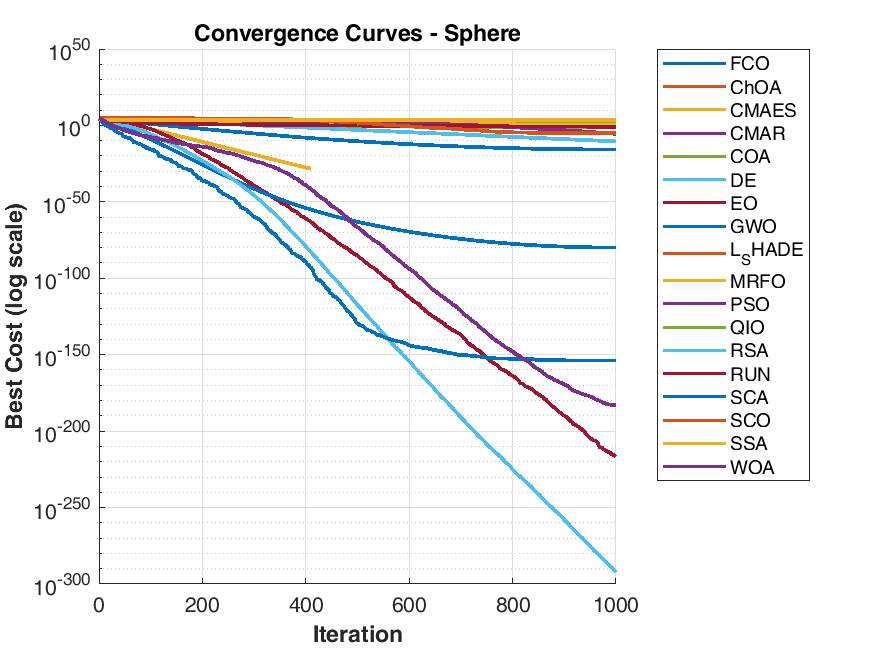

Supplement: S1 Data — (ZIP) [file pone.0341325.s004.zip › FCO Codes/CEC2005/Results/ConvergencePlots/New folder/Sphere_all_algorithms.png]

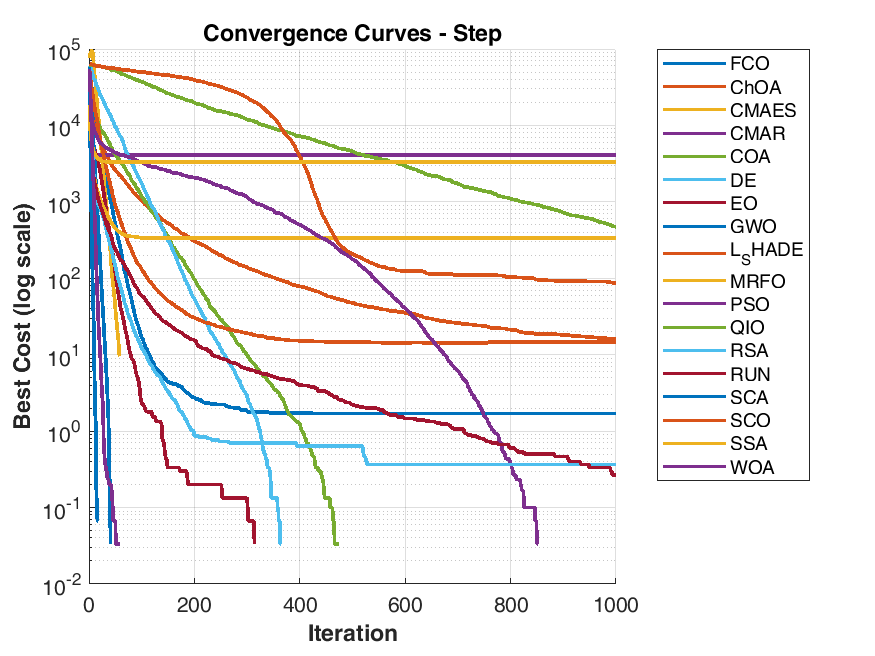

Supplement: S1 Data — (ZIP) [file pone.0341325.s004.zip › FCO Codes/CEC2005/Results/ConvergencePlots/New folder/Step_all_algorithms.png]

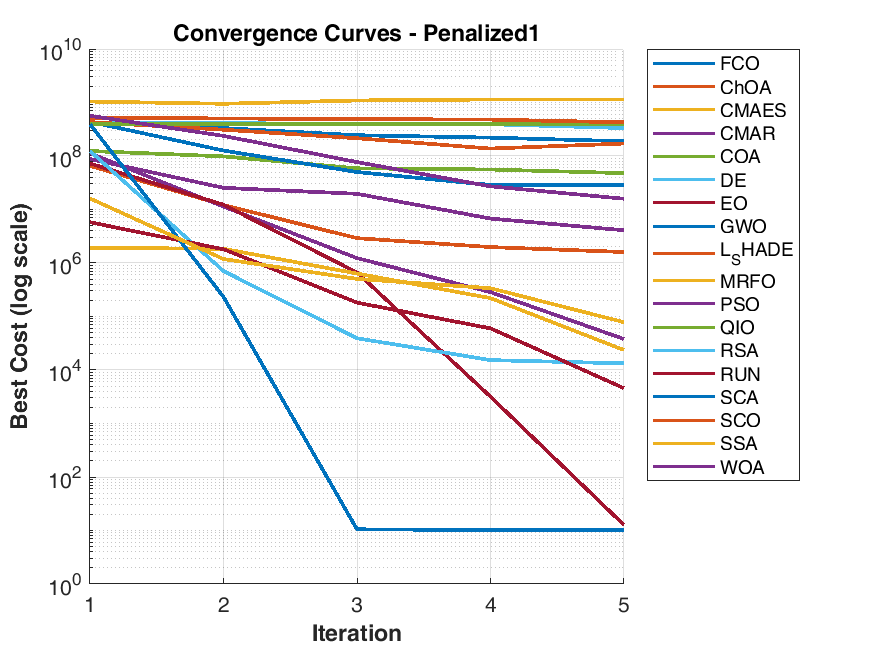

Supplement: S1 Data — (ZIP) [file pone.0341325.s004.zip › FCO Codes/CEC2005/Results/ConvergencePlots/Penalized1_all_algorithms.png]

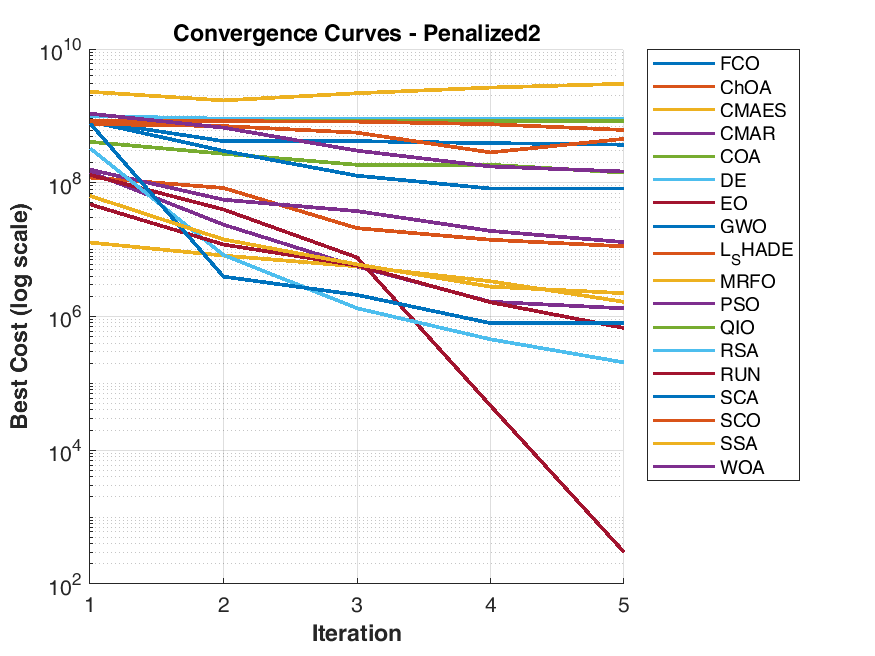

Supplement: S1 Data — (ZIP) [file pone.0341325.s004.zip › FCO Codes/CEC2005/Results/ConvergencePlots/Penalized2_all_algorithms.png]

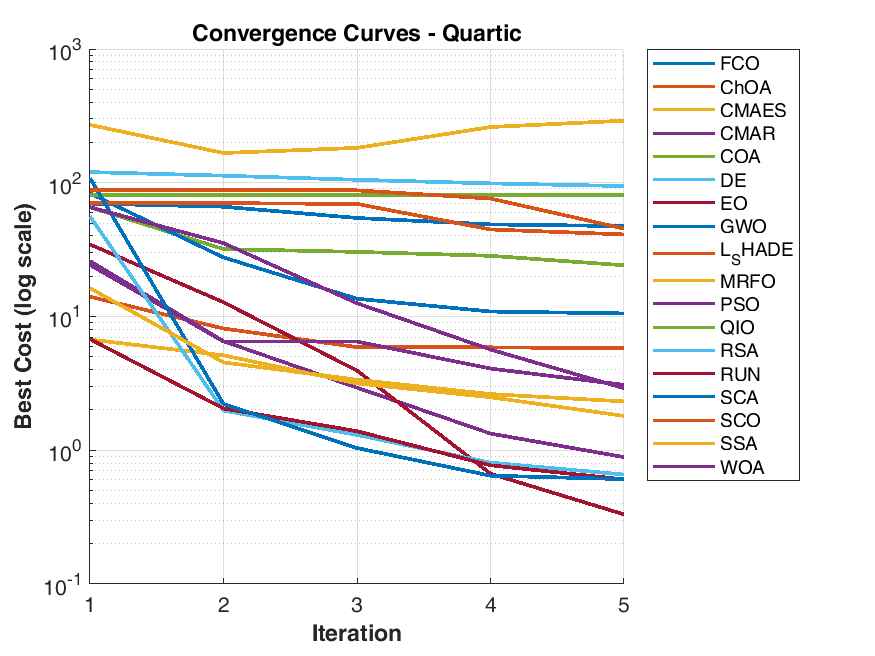

Supplement: S1 Data — (ZIP) [file pone.0341325.s004.zip › FCO Codes/CEC2005/Results/ConvergencePlots/Quartic_all_algorithms.png]

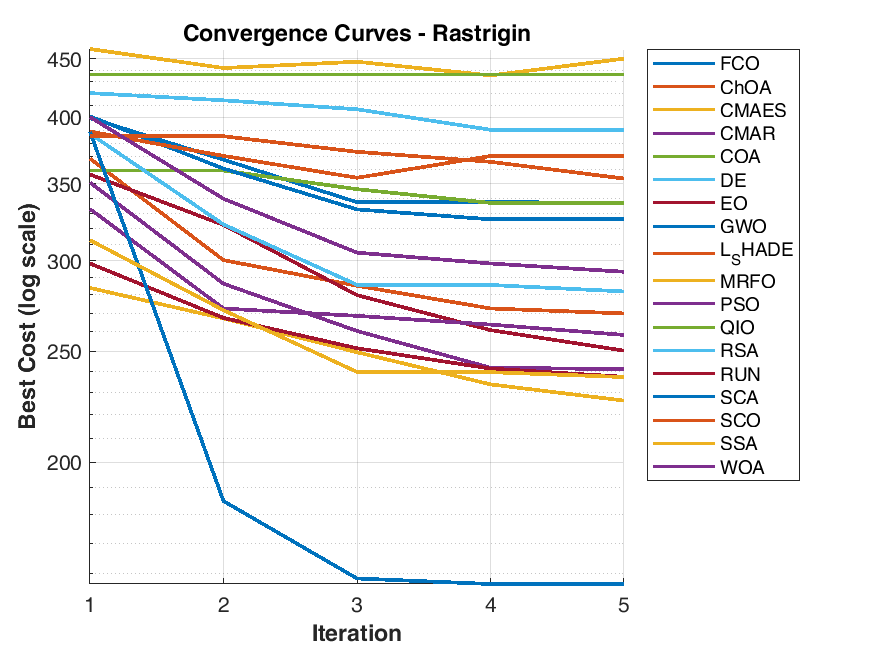

Supplement: S1 Data — (ZIP) [file pone.0341325.s004.zip › FCO Codes/CEC2005/Results/ConvergencePlots/Rastrigin_all_algorithms.png]

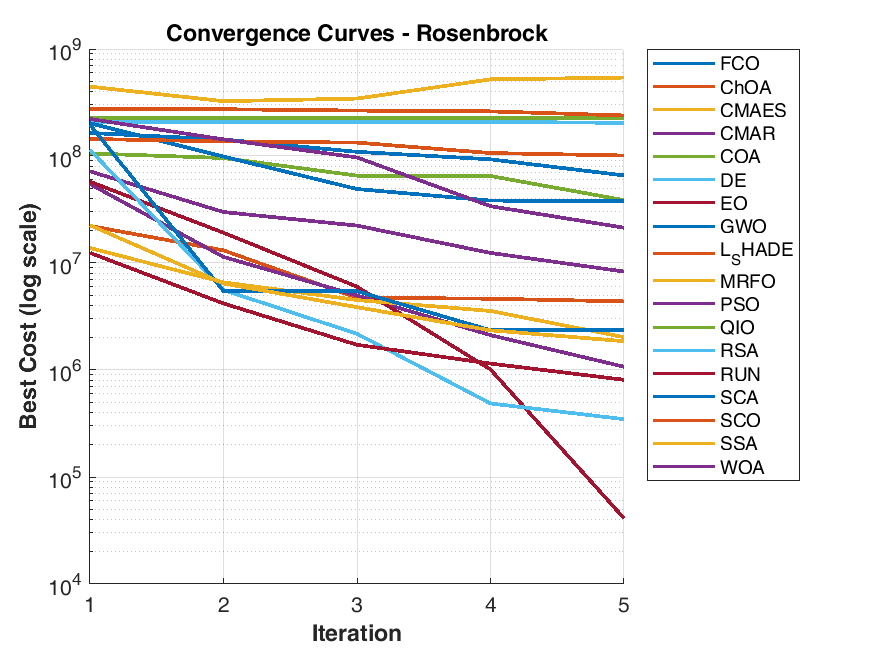

Supplement: S1 Data — (ZIP) [file pone.0341325.s004.zip › FCO Codes/CEC2005/Results/ConvergencePlots/Rosenbrock_all_algorithms.png]

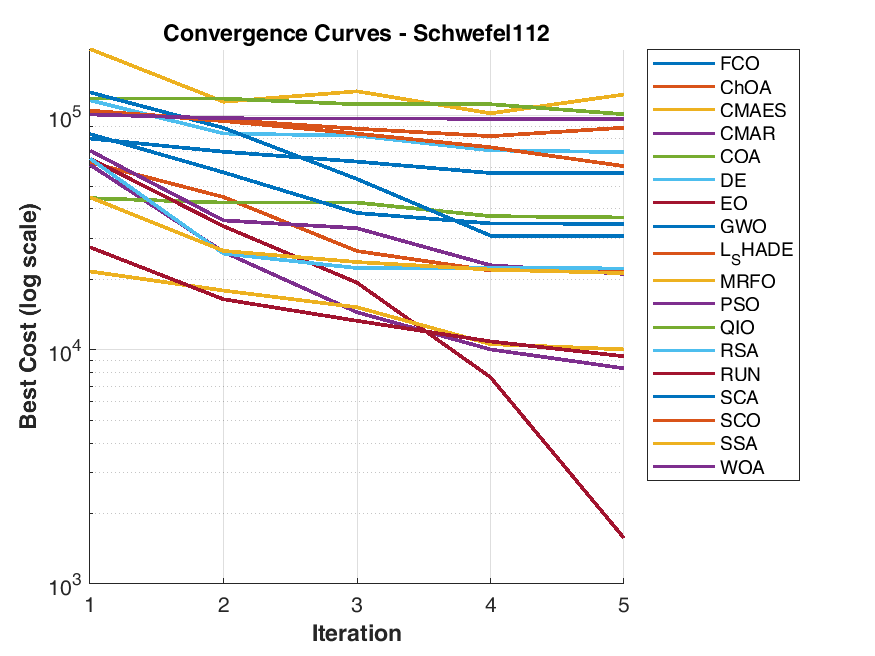

Supplement: S1 Data — (ZIP) [file pone.0341325.s004.zip › FCO Codes/CEC2005/Results/ConvergencePlots/Schwefel112_all_algorithms.png]

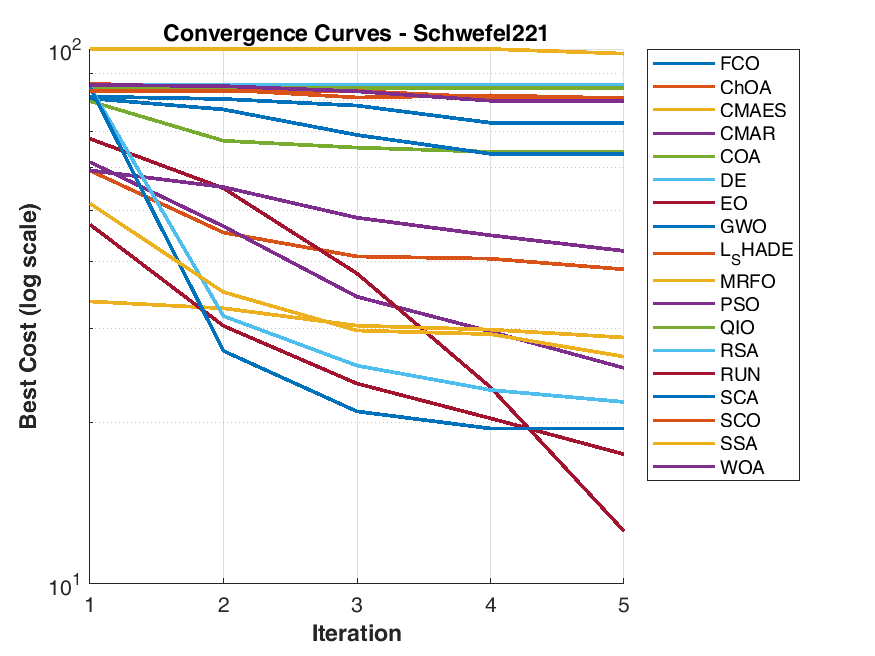

Supplement: S1 Data — (ZIP) [file pone.0341325.s004.zip › FCO Codes/CEC2005/Results/ConvergencePlots/Schwefel221_all_algorithms.png]

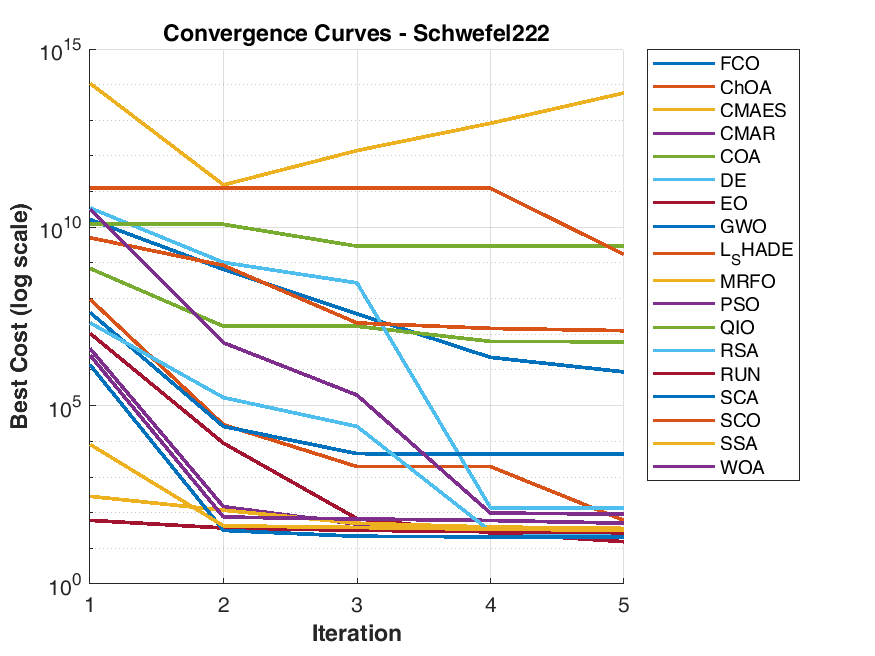

Supplement: S1 Data — (ZIP) [file pone.0341325.s004.zip › FCO Codes/CEC2005/Results/ConvergencePlots/Schwefel222_all_algorithms.png]

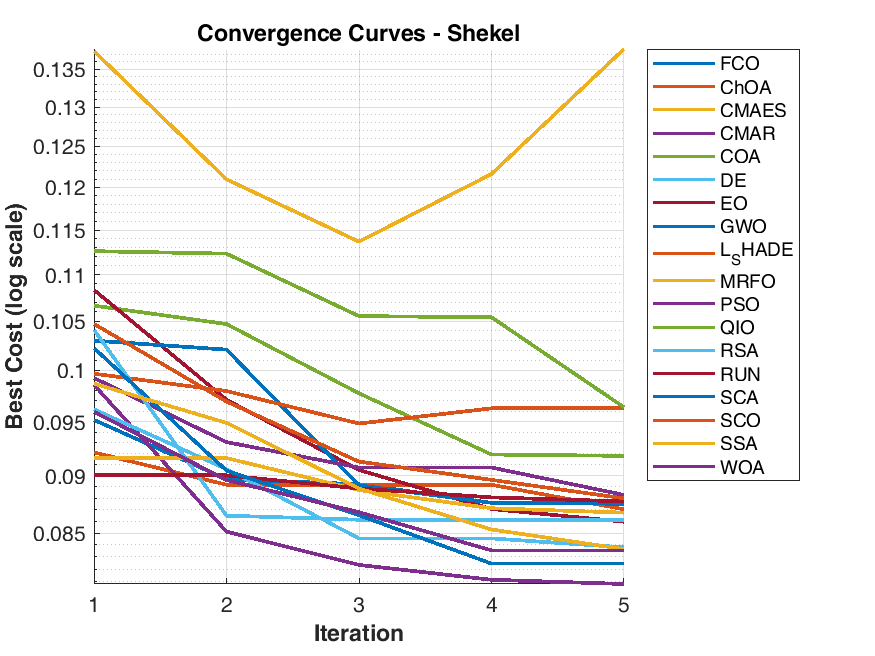

Supplement: S1 Data — (ZIP) [file pone.0341325.s004.zip › FCO Codes/CEC2005/Results/ConvergencePlots/Shekel_all_algorithms.png]

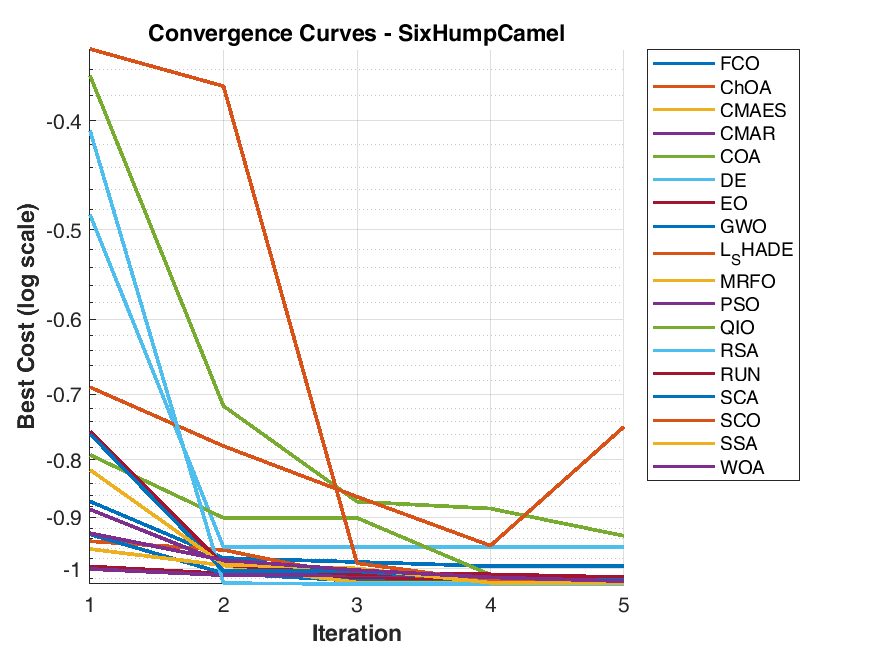

Supplement: S1 Data — (ZIP) [file pone.0341325.s004.zip › FCO Codes/CEC2005/Results/ConvergencePlots/SixHumpCamel_all_algorithms.png]

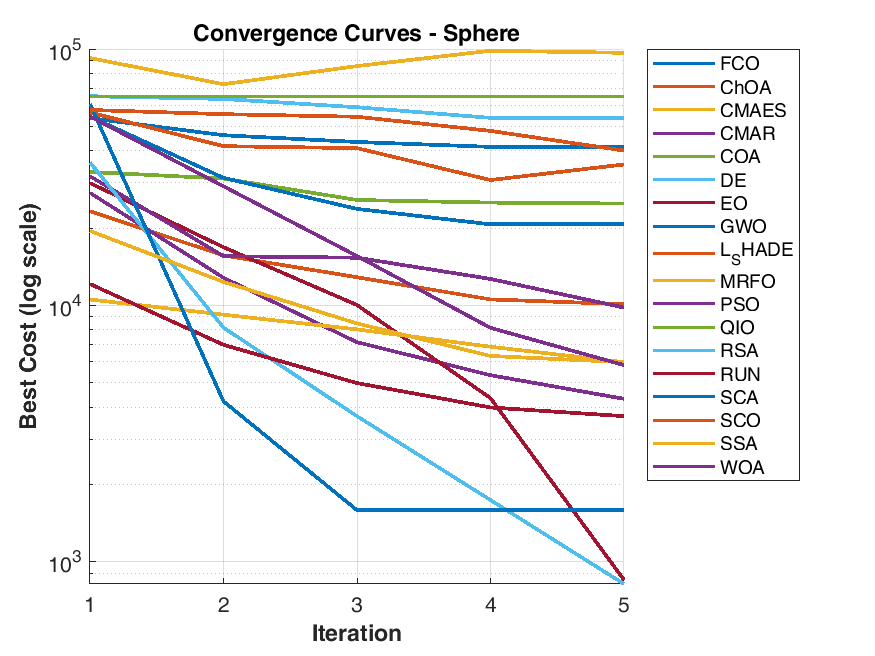

Supplement: S1 Data — (ZIP) [file pone.0341325.s004.zip › FCO Codes/CEC2005/Results/ConvergencePlots/Sphere_all_algorithms.png]

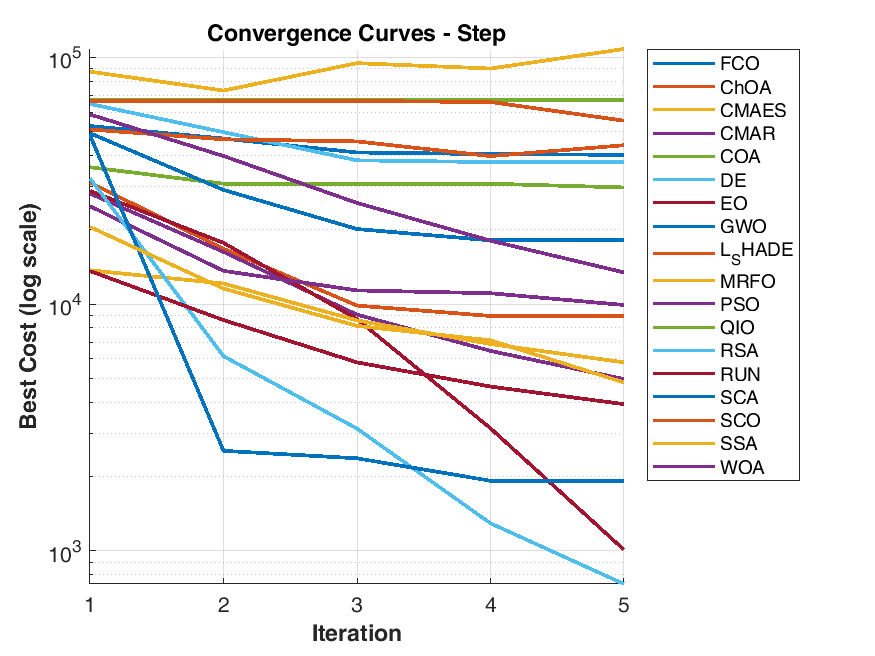

Supplement: S1 Data — (ZIP) [file pone.0341325.s004.zip › FCO Codes/CEC2005/Results/ConvergencePlots/Step_all_algorithms.png]
