# Supplementary material for: Felis Catus Optimization (FCO): A novel nature‑inspired metaheuristic algorithm
Source: PLoS One. 2026 Apr 15;21(4):e0341325. doi: 10.1371/journal.pone.0341325 (PMC13082733; doi:10.1371/journal.pone.0341325)
Supplement: Attachment — Submitted filename: Report PLOS_One_LCO_09_2025.pdf [file pone.0341325.s005.pdf]

Review of
Felis Catus Optimization (FCO): A Novel Nature Inspired Metaheuristic Algorithm

PONE-D-25-48643

Summary

The manuscript proposes a new nature-inspired metaheuristic, Felis Catus Optimization (FCO). The intended design uses a population partitioned into "male" explorers and "female" exploiters that interact on a 2-D grid of "territories" with a single dominant male per cell; additional mechanisms include aging/elimination and a tournament-based migration. The authors evaluate FCO on CEC 2005 and CEC 2017 single-objective benchmarks and on three engineering design problems (spring, welded beam, pressure vessel), provide statistical tests, and present an ablation study. A Zenodo link to source code is included.

Strengths

- **Original and engaging idea.** The territorial/dominance metaphor and explicit separation of exploration vs. exploitation are novel and interesting for the metaheuristics community.
- **Broad experimental scope.** Results are reported on two well-known CEC suites and three classic engineering testbeds.
- **Attention to robustness.** Multiple runs and nonparametric tests are included; an ablation study aims to quantify the contribution of components.
- **(Claimed) code availability.** A DOI is provided, which is valuable for reproducibility.

Weaknesses (high-level)

- **Ambiguities in the mathematical model and notation** (grid definition, cell membership, migration rules, capacity, etc.).
- **Major model–code inconsistency.** The provided MATLAB implementation does not implement the grid/territory model as described; core update rules differ from the equations.
- **Comparative baseline is too small and not representative** of current state-of-the-art CEC winners/top performers.
- **Reproducibility gaps.** Missing or underspecified defaults, seeding, and exact statistical procedures.
- **Figures and engineering-section quality issues** (incorrect panels, blurred images, and inclusion of CAE "heat maps" that are not outputs of FCO).
- **Language/terminology issues** that reduce clarity (inconsistent terms, typos, ambiguous phrasing).

Detailed list of deficiencies

A) Ambiguities and errors in the mathematical model

1. **Grid notation is self-referential/ambiguous.** The symbol G denotes both grid size and the set of cells; use separate symbols (e.g., side length S and cell set $\mathcal{G}$).
2. **No principled relation between number of cells and number of males.** The model never states how to handle cases with more males than cells (or vice versa).
3. **Agent state lacks cell membership.** Define the agent as $C_i = (x_i, \text{sex}_i, \text{age}_i, f_i, g_{j,k})$ or provide an explicit mapping $\phi : C_i \mapsto g_{j,k}$.
4. **Initialization into cells is unspecified.** Describe exactly how individuals are assigned to cells at start (random, round-robin, 1 male per cell, etc.).
5. **"Current or adjacent cell" vs. "from another cell".** These two phrasings conflict; define adjacency (4-/8-neighborhood, wrap, etc.) and whether intra-cell challenges are allowed.
6. **Mixed notation g_a, g_b vs. $g_{i,j}$.** Either keep two indices or define a flattening $a = (i-1)S+j$, $i, j \in \{1, 2, \ldots, S\}$.
7. **"Assigned based on proximity and dominance".** Specify the metric (e.g., ℓ_2 in decision space), order of assignment, handling of over-capacity k_i, and tie-breaking.
8. **"Less competitive cell".** Define the competitiveness score used in tournament migration.
9. **Aging/elimination triggers.** Besides age $>$ $\text{age}_{\max}$, clearly specify what "loss of protection/territory" means.
10. **Boundary handling.** State the policy (clamp/reflect/wrap) for steps that leave the domain.
11. **Parameter defaults/schedules.** Provide recommended values/ranges for $\alpha, \beta, \gamma, \sigma, k_{\max}$, $\text{age}_{\max}, S$ and any adaptivity.
12. **Terminology consistency.** Standardize "grid/zone/cell", "male/explorer", "female/exploiter".
13. **After a dominant male dies.** Give a concrete algorithm for (i) redistribution of females, (ii) whether a new male is created and where it is placed, (iii) succession if multiple males belong to the same cell (or explicitly forbid that case).

B) Major inconsistency between the described model and the MATLAB code

- **No 2-D grid/territories in code.** `initialize_population_grid_based` merely stratifies ~20% "males" along the *first* coordinate and places "females" near selected males; no cells, dominance per cell, or neighborhood are implemented thereafter.
- **Movement rules differ from Equations (4) and (5).**
  - Eq. (4) (males): $x_{t+1} = x_t + \alpha(x_* - x_t) + \beta\mathcal{N}(0, I)$. In code (`move_in_grid`): $x_{t+1} = x_t + \alpha[(0.7 + 1.5\,u) \odot (x_{\text{target}} - x_t)] + \varepsilon$, with $u \sim U(0,1)^d$, $\varepsilon \sim \mathcal{N}(0, 10^{-12}I)$. This is PSO-like; β is effectively absent.
  - Eq. (5) (females): $x_{t+1} = x_t + \gamma\mathcal{N}(0, \sigma^2 I)$. In code, *all* individuals use the same `move_in_grid` toward either `BestSol` or a `peer` from tournament selection—no distinct Gaussian local step for females.

- **"Tournament migration" is not migration between cells.** Code globally relocates aged individuals near the best of k random candidates; there is no selection of two cells or "less-competitive cell" concept.
- **Aging/elimination mismatch.** Code relocates and resets age when $Age > 3$ rather than eliminating and replacing individuals as the text suggests.
- **Sex/role not used after initialization.** After the first generation, behavior is not conditioned on sex; rand < 0.5 just switches the target (`BestSol` vs. peer).
- **Boundary bug.** `tournament_migration` clamps positions to $[0, 1]$ regardless of $[lb, ub]$.

C) Equation–code mapping (concise checklist)

1. Eq. (4) $\Rightarrow$ PSO-like step in code with inertia 0.7, "cognitive" $1.5 \cdot \text{rand}$, negligible Gaussian noise; missing β.
2. Eq. (5) $\Rightarrow$ not implemented; no female-specific Gaussian exploitation.
3. "Create grid $G \times G$" $\Rightarrow$ not implemented; only 1-D slotting along x_1.
4. "Proximity & dominance" $\Rightarrow$ dominance via roulette on 1/Cost; proximity used only for initial female placement around a chosen male.
5. "Territorial competition" $\Rightarrow$ no per-cell dominant to challenge/replace.
6. "Tournament-based migration" $\Rightarrow$ global relocation near best of k random candidates, not cell-based.
7. "Elimination + replacement" $\Rightarrow$ relocation + age reset.

D) Additional code-level issues

- RNG seeding not controlled (hurts reproducibility across 30 runs).
- Hard stop at 10^{-30} without linking to target baselines/tolerances.
- Inconsistent step scaling: migration uses a fixed 0.2 rather than domain-scaled steps (while initialization uses $0.1 \cdot (ub - lb)$).

E) Language, style, and clarity

- Use consistent terminology and symbols; avoid self-referential definitions.
- Correct technical names (e.g., rewrite "David on Fletcher Powell algorithm" to "Davidon–Fletcher–Powell (DFP) algorithm").
- Provide a clean symbol glossary and unify figure/table captions and variable units.

F) Engineering section (quality and relevance)

- For each reported optimum, **print all constraint values (with units)** to verify feasibility (stress, displacement, deflection, etc.).
- **Fix figures:** e.g., **Fig. 12**—panels (a)–(c) contain the wrong content and (d) is blurry.
- Remove or relocate **stress/displacement heat maps** that are **not outputs of FCO** (they belong, if at all, to a separate CAE validation appendix).

G) Inadequate comparative baseline (major)

Problem. The manuscript compares FCO to ~ 10 algorithms (mainly **GWO, PSO, GSA, LFD, TSA, MVO, SSA, WSO, AVOA, RSA, WOA**), which is neither sufficiently large nor representative of current state-of-the-art.

Why it matters (short rationale). Strong baselines should include **CEC winners/top performers** (robust, finely tuned) and **high-impact recent methods**; otherwise the claims risk cherry-picking and are hard to generalize across algorithm families (DE/ES/metaphor-free/swarm).

Required expansion. Increase baselines to ≥ 15 and **include at least**:

- **CEC winners/top performers: L-SHADE** (and variants), **CMA-ES**, **EBOwithC-MAR**.
- **Recent highly cited methods (∼last 3 years): RUN (Beyond the Metaphor)**, **Coati Optimization Algorithm (COA)**, **Quadratic Interpolation Optimisation (QIO)**.

Add further strong representatives so that classic (DE/ES) and modern CEC top methods are both covered.

Recommendation

Major revision. The idea is promising and the topic is interesting, but the manuscript requires substantial clarification and alignment of model $\leftrightarrow$ implementation, enhancements to experimental baselines, and editorial fixes before it can be reliably evaluated.
