# Supplementary material for: Felis Catus Optimization (FCO): A novel nature‑inspired metaheuristic algorithm
Source: PLoS One. 2026 Apr 15;21(4):e0341325. doi: 10.1371/journal.pone.0341325 (PMC13082733; doi:10.1371/journal.pone.0341325)
Supplement: Attachment — Submitted filename: Report PLOS_One_LCO_Round_2_11_2025.pdf [file pone.0341325.s007.pdf]

Review of
Felis Catus Optimization (FCO): A Novel Nature Inspired Metaheuristic Algorithm

PONE-D-25-48643

Strengths

- Original and appealing concept with clear exploration/exploitation roles.
- Expanded experimental scope (CEC suites + engineering problems).
- Improved methodological narrative (male triplet moves, female Gaussian exploitation, rejuvenation/noise).
- Public code DOI announced, moving toward reproducibility.
- Use of statistical summaries and (some) post-hoc analyses.

Weaknesses

- Model–code mismatch: the current MATLAB code does not implement the declared grid/territorial framework.
- Grid definition, cell membership, and adjacency remain only loosely specified.
- Baseline portfolio still problematic (acronym swaps; missing key state-of-the-art winners).
- Baseline implementations show correctness issues (e.g., EBOwithCMAR, CMA-ES whitening).
- Reproducibility pipeline incomplete (seeds, defaults, exact stats procedure).
- Figures and tables require quality and numerical corrections.
- Engineering section mixes in non-FCO outputs and lacks feasibility checks.
- Terminology/notation inconsistency persists across sections.

Required revisions for the next round

- **Resolve the model–code mismatch (critical).** Either (i) implement the full grid/territory model (cells, dominant male per cell, explicit adjacency, redistribution), or (ii) revise the manuscript to faithfully describe the implemented *non-grid* ecological dynamics (male triplet, female Gaussian, ENI, aging, mating). Provide concise pseudocode that matches the code line-for-line.
- **Formalize the grid (if kept).** Define the mapping from $\mathbb{R}^d$ to cells; specify grid size, indexing, and adjacency (4/8, edges/torus). Include agent *cell membership* in the agent state and state the relation between #cells and #males.
- **Specify life-cycle events.** Precisely describe: succession when a dominant dies; redistribution of females; rules for adding/placing a new male; definition of "less-competitive cell"; capacity and assignment of females (metric, order, tie-breaking).

- **Equations $\leftrightarrow$ implementation.** Demonstrate 1:1 alignment for all update rules and parameters (female-local step, rejuvenation vs. migration). Include the exact parameter schedules used in experiments.
- **Fix code issues in FCO_v8.m**
  - Dynamic Territory Scaling bug: There is some nonsense in your code, you have
    `Age(i)=Age(i)+1;`
    and only then you use:
    `if Age(i)==0, R = R*(1+beta_RS); else R = R*(1-beta_RS/2); end`
    so only the "else" part is always used. Use an *improved-today* flag and maintain a *per-male* radius R_i (not global).
  - Make sex/role persistent or explicitly state rank-based roles; keep behavior consistent with the manuscript.
  - Normalize the diversity trigger (`noise_thr`) to domain scale; unify relative vs. absolute step sizes.
  - Document computational cost (multiple `pdist2`) and the FE/time budget parity used in comparisons.
- **Strengthen and correct baselines.**
  - Expand to ≥ 15 methods including **L-SHADE (and variants)**, **CMA-ES**, **EBOwith-CMAR**, **RUN**, **Coati Optimization Algorithm**, **Quadratic Interpolation Optimisation (QIO)**.
  - Fix acronym swaps: *Coati* $\neq$ *Coyote* (both COA); *Quadratic Interpolation Optimisation* $\neq$ *Quantum-Inspired/Quantum-Invasive* (both QIO). Use full names alongside acronyms in tables/figures.
  - For each baseline, cite the exact source repo/commit; apply identical FE budgets and report the same metrics (best/mean/std, ranks, post-hoc).
- **Baseline correctness**
  - Replace non-faithful **EBOwithCMAR** with the official implementation or a byte-for-byte reproduction.
  - Correct **CMA-ES** whitening: the computation of $C^{-1/2}$ must include the transpose $B^\top$ in the eigendecomposition step; cross-validate against the reference implementation.
- **Statistics & reproducibility.** State the full statistical pipeline (which tests on which metrics; Friedman + Nemenyi/Holm; how CD is computed). Release seeds, software versions, and a minimal script to regenerate all tables/figures. Provide a single table of FCO defaults/ranges and schedules.
- **Data/code availability (consistency).** Ensure *all* code *and* data (benchmark scripts, raw results, seeds) are publicly available at the DOI referenced in the paper; remove any conflicting "available upon request" phrasing.
- **Figures and tables.** Provide high-quality graphics ($\geq$ 300 DPI; consistent fonts/legends; vector where possible) and fix erroneous/blurred panels (e.g., multi-panel figure with mismatched content). In **Table 5**, correct missing minus signs in search-space lower bounds and negative optima; unify numeric formatting.
- **Language and notation.** Unify terminology (grid/zone/cell; male/explorer; female/exploiter) and indices; add a compact symbol glossary; remove residual template artifacts and typos.
