# Supplementary material for: Felis Catus Optimization (FCO): A novel nature‑inspired metaheuristic algorithm
Source: PLoS One. 2026 Apr 15;21(4):e0341325. doi: 10.1371/journal.pone.0341325 (PMC13082733; doi:10.1371/journal.pone.0341325)
Supplement: S1 Data — (ZIP) [file pone.0341325.s004.zip › FCO Codes/Read ME.docx]

**Felis Catus Optimization (FCO): A Novel Nature‑inspired Metaheuristic Algorithm**

**MATLAB Codes and Datasets**

====================================================================

1. Description

--------------

This package contains the MATLAB implementation of the FCO algorithm for Felis Catus Optimization (FCO): A Novel Nature‑inspired Metaheuristic Algorithm

2. Contents

-----------

- Codes/: MATLAB .m files implementing FCO.m, ChOA.m, CMAES.m, CMAR.m, COA.m, DE.m, EO.m, GWO.m, L_SHADE.m, MRFO.m, PSO.m, QIO.m, RSA.m, RUN.m, SCA.m, SCO.m, SSA.m, WOA.m.

And all functions for test.

- BenchMarks 2005, 2017

- Tension/Compression Spring Design

- Welded Beam Design

- Pressure Vessel Design

3. Requirements

---------------

- MATLAB R2019a or newer.

- Optimization Toolbox (optional, for certain validation scripts).

4. How to Run

-------------

1. Open Folder(CEC 2005/2017/Spring/Wolded/Pressure
2. Run Main.m.
3. Get Results as text
4. Figures in Results folder

5. License

----------

Distributed under the Creative Commons Attribution 4.0 International License (CC BY 4.0).
