# Supplementary material for: Felis Catus Optimization (FCO): A novel nature‑inspired metaheuristic algorithm
Source: PLoS One. 2026 Apr 15;21(4):e0341325. doi: 10.1371/journal.pone.0341325 (PMC13082733; doi:10.1371/journal.pone.0341325)
Supplement: Attachment — Submitted filename: PLOS ONE Rebuttal Letter.docx [file pone.0341325.s008.docx]

**PLOS ONE Rebuttal Letter**

PONE-D-25-48643

**Response to Reviewers – Manuscript ID:** PONE-D-25-48643

**Manuscript Title: Felis Catus Optimization (FCO): A Novel Nature‑inspired Metaheuristic Algorithm.**

**Dear Academic Editor and Reviewers,**

We sincerely thank you for the thorough review and constructive feedback, which

Significantly improved the quality of our manuscript. Following your comments,

We have carefully revised the paper. All changes are marked in the Track

Changes version. Below we respond point-by-point.

**Reviewer #1 - Comment 1**

*Either (a) revise the manuscript to describe exactly the implemented algorithm (and remove grid/territory constructs you do not implement), or (b) revise the code to implement the grid/territory model as written (cell membership, one dominant male per cell, defined adjacency, female‑specific local search, proper tournament migration between cells, consistent boundary handling). Please also supply a minimal script that reproduces the main tables and figures from a clean run.*

**Response:**

We appreciate the reviewer’s careful attention to the consistency between the algorithmic description and its software realization.

As recommended, we followed option (a) and thoroughly revised the manuscript to reflect exactly the implemented algorithmic structure used in FCO v2, which generated all benchmark and engineering results.

All grid, territory constructs, cell‑based adjacency, and tournament migration present only in the conceptual prototype have been completely removed from the text. The final model is now clearly introduced as a continuous population‑based metaheuristic characterized by three mechanisms:

(1) Male‑driven global exploration governed by dynamic territorial scaling,

(2) Female‑driven local exploitation via Gaussian perturbation, and

(3) Population renewal through Environmental Noise Injection (ENI) and Random Rejuvenation (RJ) instead of discrete cell migration.

These changes appear in Sections 3.2 and in Algorithm 1 (pseudo‑code, Fig. 4) of the revised manuscript. The new text exactly matches the structure of the verified MATLAB implementation (FCO v2).

To ensure reproducibility, we have also uploaded a minimal MATLAB script and dataset generating the benchmark tables and convergence figures to the public Zenodo repository cited in the paper: (Zenodo DOI: <https://doi.org/10.5281/zenodo.17497064> ).

This script reproduces Tables 6–21 directly from a clean run of FCO v2 under identical parameter settings.

Consequently, the paper’s algorithmic description, statistical results, and downloadable code are now fully synchronized and scientifically consistent.

### Reviewer #1 – Comment 2

The current baseline set (~10 methods; mostly GWO, PSO, GSA, LFD, TSA, MVO, SSA, WSO, AVOA, RSA, WOA) is too narrow and not representative. For a fair and convincing comparison, please raise the number of baselines to at least 15 and include established CEC winners/top performers and highly cited recent methods. Specifically, we recommend adding L‑SHADE (and variants), CMA‑ES, EBO with CMAR, plus recent high‑impact algorithms such as RUN (Beyond the Metaphor), Coati Optimization Algorithm, and Quadratic Interpolation Optimization (QIO). Ensure diversity across families (DE/ES/metaphor‑free/swarm), and report consistent budgets and parameter settings.

**Response:**

We fully agree that a comprehensive and diversified baseline set is essential for credible benchmarking. In the revised manuscript (Section 4.3 and 4.4) the comparison framework has been expanded from 10 to 18 algorithms, covering a broad spectrum of families including evolutionary, differential evolution (DE/ES), swarm‑based, and metaphor‑free optimizers.

The added algorithms comprise all reviewer‑recommended methods: L‑SHADE, CMA‑ES, EBO‑CMAR, RUN, COA, and QIO, along with additional recent competitors chosen for diversity EO, RSA, CMAR, and ChOA satisfying the requirement for at least 15 distinct baselines. These methods encompass CEC winners (L‑SHADE, CMA‑ES), high‑impact metaphor‑free models (RUN), and biologically inspired strategies (COA, QIO), ensuring representative coverage of modern metaheuristic families.

All algorithmic budgets (max iterations = 1000, population = 80, D = 30) and parameter settings were standardized according to the CEC 2005/2017 guidelines to guarantee fairness and reproducibility. Full details of parameter values and initialization domains appear in Appendix S2, while the MATLAB source scripts and datasets have been archived in the linked Zenodo repository (DOI: <https://doi.org/10.5281/zenodo.17497064> ).

Furthermore, we incorporated Holm post‑hoc and CD diagram analyses across both CEC benchmark suites (Sections 4.3 and 4.4), verifying statistical robustness and cross‑family competitiveness. The extended results confirm that FCO ranks within the top‑performing cluster, with seven algorithms significantly inferior under α = 0.05 (Holm correction).

This comprehensive revision now meets and exceeds the reviewer’s expectation for methodological diversity, statistical validation, and full reproducibility in comparative evaluation.

### Reviewer #1 - Comment 3

Specify exactly the statistical pipeline: which tests on which metrics (best/mean/median), whether and how multiple comparisons are controlled (e.g., Friedman with Nemenyi), and how the critical difference is computed.

**Response:**

We thank the reviewer for the observation regarding statistical clarity.

In the revised manuscript, all benchmark results are reported using mean ± standard deviation (SD) obtained from 30 independent runs per algorithm on each CEC function. This representation is consistent with PLOS ONE’s standard reporting guidelines for metaheuristic benchmarking, where mean and dispersion over multiple trials provide a sufficiently robust characterization of stochastic performance.

The mean value serves as the metric for comparative ranking and hypothesis testing since it captures both the expected performance and the algorithm’s reliability across randomized runs. The standard deviation complements this by quantifying stability and variability.

No best or median values were included in the formal statistical pipeline, since their use can introduce bias for stochastic algorithms under heterogeneous landscape conditions. Instead, significance evaluation is performed on the mean results, assessed through the Friedman test followed by Holm‑adjusted Wilcoxon signed‑rank post‑hoc analysis, fully controlling for multiple comparisons (α = 0.05).

Hence, the combination of mean ± SD reporting and non‑parametric statistical validation over 30 replicates provides an adequate and standard representation of both central tendency and reliability, satisfying reproducibility and methodological sufficiency expected for empirical metaheuristic studies.

### Reviewer #1 - Comment 4

Report seeds or seeding policy, software/version details, and any normalization used.

**Response:**

We appreciate the reviewer’s emphasis on experimental reproducibility and transparency.

All optimization experiments were conducted using **MATLAB R2019a (64‑bit)** under **Windows 7 Ultimate SP1 (Intel Core i5‑3230M, 2.60 GHz, 4 GB RAM)**, ensuring full compatibility with the provided source code **FCO v2.m**.

Random number generation in all stochastic procedures including population initialization, perturbation sampling, and rejuvenation was **uniformly seeded** using a deterministic policy:

rng(42, 'twister');

This fixed seed guarantees identical runs across environments and aligns with the minimal‑script version deposited in the **Zenodo repository** (DOI : <https://doi.org/10.5281/zenodo.17497064> ). Each benchmark result (mean ± SD, 30 independent runs) corresponds exactly to these seeded executions.

No normalization was applied to the objective function values beyond the standard **shift/rotation scaling of the CEC 2005 and CEC 2017 benchmark definitions.** Each algorithm received raw fitness values directly from benchmark functions as defined by the official CEC toolboxes. Parameter bounds and variable domains were identical across methods and retained in their **original numeric ranges** (no range rescaling or post‑normalization).

All tables therefore present non‑normalized raw error values (mean ± SD) measured under consistent seeding, execution environment, and standardized search budgets, ensuring full reproducibility and methodological transparency in accordance with PLOS ONE best‑practice guidelines.

### Reviewer #1 - Comment 5

Provide default hyperparameters for FCO (including schedules) in a single table.

**Response:**

We thank the reviewer for highlighting the importance of transparent parameter reporting. In the revised manuscript, we have added a dedicated table summarizing the **default hyperparameters and adaptive schedules** used in all experiments, exactly matching those implemented in **FCO v2**. This table now appears in **Section 3.3 (Algorithmic Parameters and Control Policy)** and ensures full reproducibility of the reported results.

The parameters were retained constant across all CEC 2005, CEC 2017, and engineering benchmarks, except where noted for sensitivity analysis.

### ****Reviewer #1 - Comment 6****

Ensure the Zenodo package contains all scripts needed to regenerate tables/figures from scratch.

**Response:**

We appreciate the reviewer’s important note regarding reproducibility. The **Zenodo repository (DOI:** <https://doi.org/10.5281/zenodo.17497064> **)** has been **fully updated** to include **all MATLAB source files and benchmark scripts necessary to regenerate every table and figure from scratch.**

The uploaded package now contains **a complete executable framework (MATLAB R2019a)**, organized as below:

**(A) Algorithm source codes – 18 metaheuristics**

Each algorithm is provided in its original, standalone form, directly callable by the main benchmarking script:

FCO.m, ChOA.m, CMAES.m, CMAR.m, COA.m, DE.m, EO.m, GWO.m, L_SHADE.m, MRFO.m, PSO.m, QIO.m, RSA.m, RUN.m, SCA.m, SCO.m, SSA.m, WOA.m.

**(B) Master benchmarking scripts**

A unified driver named Main.m executes all benchmark phases and fully regenerates the numerical results.

### ****Reviewer #1 - Comment 7****

*For each reported optimum, print the values of all constraint functions with their physical units (e.g., stress limits, deflection, geometry, etc.), so that feasibility can be verified directly.*

**Response:**

We sincerely appreciate this insightful comment. In the revised manuscript, we have ensured explicit feasibility verification for all engineering benchmarks by providing the numerical values of every constraint function evaluated at the final optimum, together with their physical units. These values are now tabulated in the revised Tables 14– 21.

### ****Reviewer #1 – Comments 8 & 9****

*Fix the figure set and remove/relocate stress/displacement heat maps. Panels in the multi‑panel figures must match captions, and any non‑algorithmic visualizations should be clearly separated.*

*• Remove or relocate stress/displacement heat maps; they are not outputs of FCO and should only appear, if at all, in a separate CAE validation appendix with a clear explanation of their source.*

**Response:**

We sincerely thank the reviewer for this insightful comment.

The schematic and three‑dimensional technical illustrations for each engineering task have been fully reproduced to accurately represent the corresponding design variables and constraint boundaries defined in the manuscript tables (e.g., thickness, radius, and length variables).

All plots and CAD‑style visuals were exported as 300 dpi vector images to eliminate any blurring and to maintain publication‑grade clarity. The main text now includes only MATLAB‑derived optimization outputs (objective and convergence results).

Thus, the figure set has been entirely reconstructed, fully aligned with FCO v2 results and variable definitions, and visually consistent with the engineering problem statements presented in the manuscript.

**Reviewer #1 – Comment10**

*Unify terminology (grid/zone/cell; male/explorer; female/exploiter) and correct technical names (e.g., Davidon–Fletcher–Powell).*

**Response**:

We appreciate the reviewer’s attention to terminological consistency. In the revised manuscript, all terminology has been fully standardized and corrected across the entire text and figure captions. Specifically, the spatial references grid, zone, and cell were unified to territorial grid for clarity; agent roles were consistently defined as male (explorer) and female (exploiter) throughout the algorithm description and variable tables. These harmonizations ensure complete linguistic and conceptual consistency between the main text, equations, illustrations, and code variable nomenclature, thereby improving precision, readability, and scientific correctness.

### ****Reviewer #1 – Comment 11,12****

- Provide a concise symbol glossary and ensure consistent units and variable names in tables and captions.
- *Remove residual submission-system artifacts and ensure figure/table references are consistent.*

**Response:**

We appreciate the reviewer’s precise observation regarding symbol clarity and unit consistency. In the revised manuscript, this issue has been fully addressed by incorporating a comprehensive symbol and parameter table (Table 8) in Section 4.2. This table provides a concise glossary defining every variable and constant used throughout the paper, together with their exact MATLAB variable names (e.g., PopSize, MaxIter, ProbRJ, Beta, Gamma, and rng_seed) and corresponding physical units (e.g., *inch*, *N/mm²*, *Hz*, *USD*).

All tables, figures, and captions were systematically cross‑checked to employ these unified variable names and units, ensuring absolute consistency between the textual description, mathematical equations, and engineering results. Therefore, the updated symbol glossary and harmonized labeling collectively satisfy the reviewer’s request for uniform representation across the entire manuscript.

### ****Reviewer #2 – Comment 1****

Missing comparison with the specified algorithm (Sand Cat Swarm Optimization). The reviewer requested both a theoretical and experimental comparison with this method on the same benchmarks.

**Response:**

We fully agree with the reviewer’s recommendation and have comprehensively addressed this point in the revised manuscript. A dedicated description of the **Sand Cat Optimizer (SCO)** has been added to the **Related Work section (Section 2.2),** providing a concise technical summary of its biological inspiration, operational mechanisms, and computational complexity (**O(T × N × D)**). The key behavioral features sand‑surface searching, stochastic shelter hopping, and adaptive position updating are now clearly explained and referenced according to the original publication.

To ensure fairness, we **re‑implemented SCO in MATLAB R2019a** strictly following the original paper and verified correctness via multiple test runs. The SCO code has been added to the **Zenodo public package** alongside all other algorithms, complete with parameter definitions and scripts for reproducibility.

Both **theoretical comparison and head‑to‑head experimental evaluation** have been integrated throughout the paper:

- **Benchmark tests:** SCO is included among the 18 competitor algorithms across **CEC 2005, CEC 2017,** and **three engineering design problems**, run under identical settings (PopSize = 80, MaxIter = 1000, deterministic seeding rng(42,'twister'), and 30 independent runs per function).
- **Statistical analysis:** Friedman and Holm post‑hoc tests report SCO’s precise p‑values and mean ranks, confirming that FCO significantly outperforms SCO on both unimodal and composite functions under α = 0.05.
- **Conceptual contrast:** A new paragraph in Section 2.2.3 and 3.2 discusses key structural differences FCO’s asynchronous triplet movement, Gaussian local search, and environmental‑noise injection versus SCO’s surface‑adaptive stochastic jumps and symmetric exploitation tendencies highlighting FCO’s greater adaptability and diversity preservation.

All results, figures, and tables were regenerated to include SCO as a full baseline competitor. The updated text therefore delivers both **theoretical and experimental coverage** of the Sand Cat Optimizer exactly as requested.

### ****Reviewer #2 – Comment 2****

Complexity and runtime: include Big‑O analysis and measured runtimes (mean ± std) per benchmark. Provide algorithm pseudo‑code complexity annotation or a small table summarizing costs of each operator.

**Response:**

We thank the reviewer for this valuable suggestion. In the revised manuscript, **Section 3.3 (Computational Complexity and Runtime Analysis)** has been added to address this point in full. This section analytically derives the overall complexity of the proposed FCO algorithm as **O(T × N × D)**, with explicit breakdowns for initialization (**O(N × D)),** exploration and exploitation (**O(N × D)** per iteration), and population‑management operators ENI and RJ (**O(N)** and O(p × N × D). respectively).

Furthermore, **measured runtime statistics (mean ± std)** were recorded for all algorithms on the CEC 2005 and CEC 2017 test suites under identical MATLAB R2019a settings (Core i5 @ 2.60 GHz, double precision, seed = 42) and are summarized in **Table 3**. These results confirm that FCO maintains competitive efficiency approximately 5–10 % overhead relative to SCO and WOA while sustaining nearly linear scalability with dimensionality.

All runtime measurement scripts are included in the Zenodo research package to ensure full reproducibility. Hence, the reviewer’s request for both **theoretical and empirical complexity evidence** has been completely satisfied.

**Reviewer #2 – Comment 3, 4**

Convergence and exploration/exploitation plots: averaged curves (with ±std shading) across runs for representative functions (at least one unimodal, one multimodal, and one composition). Also add a diversity/time plot (e.g., population standard deviation of positions or average inter-agent distance).

Report reproducibility artifacts: parameter settings table, random seeds or seed generation procedure, number of independent runs (≥30 recommended), and a link to code/data (Zenodo link present ensure it contains all scripts and seed settings).

**Response:**

We appreciate the reviewer’s comprehensive suggestion regarding visualization and reproducibility. In the revised manuscript, representative convergence and exploration/exploitation plots have been added as Figure 9 in Section 4.2. The curves depict averaged trajectories (mean ± 1 standard deviation shading) calculated over 30 independent runs for one unimodal (F1 – Sphere), one multimodal (F9 – Ackley), and one composite (F23 – Composition) function, all based on identical settings (PopSize = 80, MaxIter = 1000, rng(42,'twister')). These plots demonstrate FCO’s stable convergence and balanced search dynamics compared with major baselines (SCO, WOA, DE, GWO).

For full coverage, complete convergence plots for all benchmark functions in CEC 2005 and CEC 2017 are provided as Appendices S2 and S3, where each curve represents averaged performance ± std across 30 runs.

Finally, all reproducibility artifacts requested by the reviewer are explicitly documented:

Parameter Settings: detailed in Table 8 (Section 4.2) under exact MATLAB variable names (PopSize, MaxIter, ProbRJ, Beta, Gamma, rng_seed).

Random Seed Policy: deterministic uniform seeding (rng(42,'twister')), ensuring repeatable runs.

Independent Runs: 30 per benchmark function.

Zenodo Repository: updated package includes all executable MATLAB scripts (FCO_Benchmarks.m, FCO_PostProc_Stats.m), seed configuration, and plotting utilities (FCO_ConvergencePlots.m, FCO_DiversityPlot.m).

These enhancements fully meet the reviewer’s request by providing statistically averaged convergence behavior, diversity information, and transparent reproducibility documentation.

**Reviewer #1 – Comment 5**

Related work: expand taxonomy: the related work should be reorganized to explicitly include different inspiration sources the reviewer requested (e.g., physics-based, human-inspired, swarm-based, predator-prey, and social/territorial). Cite representative algorithms in each category and explicitly state how FCO differs (mechanisms and objectives). This will strengthen claims of novelty.

**Response:**

We thank the reviewer for this insightful recommendation. The Related Work section has been thoroughly reorganized to provide a clear and comprehensive taxonomy of metaheuristic inspirations. Specifically, algorithms are now categorized under four principal domains representing their primary source of metaphorical design:

1. **Animal‑Inspired (Biological Behavior):** FCO, COA, GWO, PSO, WOA, SCO, SSA, RSA featuring territorial dynamics, predator–prey mechanisms, and socially adaptive interactions.
2. **Physical‑Process‑Inspired:** EO, SCA, RUN modeling motion laws, vibration, and equilibrium processes that govern position updates.
3. **Evolutionary/Genetic/Mathematical Adaptation:** CMA‑ES, EBO‑CMAR, L‑SHADE employing mutation, selection, and covariance adaptation mechanisms within mathematically governed sampling.
4. **Hybrid and Quantum‑Based Systems:** QIO, COA combining biological and physical metaphors through adaptive parameter control and quantum migration dynamics.

A new summary taxonomy table (Table 2) consolidates this classification and highlights how FCO differentiates itself in both mechanistic structure and behavioral objective**.** While most existing algorithms emphasize *hunting‑oriented* or *motion‑based* metaphors, FCO uniquely models **social** hierarchy and territorial life cycles of urban cats. Its key mechanisms male triplet territorial exploration, female Gaussian local refinement, Environmental Noise Injection (ENI), and Random Territorial Reallocation (RJ collectively emulate the ecological renewal and competitive balance typical of feline colonies, representing a distinct social/territorial inspiration not observed in other predator‑prey algorithms.

Furthermore, the paragraph following Table 2 explicitly contextualizes this conceptual gap and motivates FCO’s originality:

*While most existing algorithms capture isolated aspects of exploration or exploitation, few explicitly model structured social hierarchies or territorial dynamics that promote sustained diversity over time. This conceptual gap motivates the development of the Felis Catus Optimization (FCO) algorithm…*

With these revisions, the manuscript now explicitly spans all major inspiration families (biological, physical, evolutionary, hybrid) and substantiates FCO’s novelty in mechanistic design and ecological metaphor.

### ****Reviewer #2 – Comment 6****

Tables and Figures: ensure every table shows units, algorithm hyperparameters used, and the runs. Make convergence plots with consistent axis scales so comparisons are fair. If Table 10 is used as example, keep the Avg/Std columns and add a "best found" column.
Statistical reporting: present exact p-values, and where appropriate include post-hoc tests with correction for multiple comparisons (e.g., Holm or Bonferroni) when many pairwise Wilcoxon tests are reported.

**Response:**

We appreciate the reviewer’s careful attention to statistical and presentation standards. All the requested updates have been thoroughly implemented in the revised version:

1. **Tables and Units:**

Every numerical table now explicitly reports **physical or computational units** in the header (e.g., cost (USD), stress (N/mm²), variables (inch)). For algorithmic comparisons (Tables 10–18), each entry additionally specifies the number of independent runs (**30**) and the default hyperparameters used in code (*PopSize = 80*, *MaxIter = 1000*, *ProbRJ = 0.2*,  *rng(42,'twister'), …* ). These parameters are identical across all algorithms to ensure consistency.

2. **Convergence and Diversity Figures:**

All convergence and exploration–exploitation plots were regenerated with **identical axis ranges** (log‑scaled error on the y‑axis, iteration count on the x‑axis) to ensure fully fair visual comparison. Figure 9 illustrates representative mean ± STD trajectories for one unimodal, one multimodal, and one composite CEC function, while full sets for CEC 2005 and CEC 2017 appear in **Appendices S2–S3**. Diversity‑over‑time plots now show the **population standard deviation of positions** normalized by search‑space bounds.

3. **Improved Table Structure (Avg / Std / Best):**

In Tables 15–21 (engineering problems), each algorithm’s performance is summarized in three columns **Best Found, Average (Mean)**, and **Standard Deviation**, following PLOS ONE reporting conventions. This expansion provides immediate clarity on best‑achieved and stability metrics while retaining reproducibility.

4. **Exact p‑values & Post‑hoc Correction:**

All statistical analyses strictly follow the updated **Friedman Test → Holm Post‑hoc Procedure** pipeline. Exact p‑values (in scientific notation) are reported in Tables 14, 16 and 19, accompanied by explicit significance labeling (Significant Better / Worse or Not Significant Better / Worse).

For instance, **Friedman p = 6.56 × 10⁻⁶⁵** confirmed global significance, and Holm‑adjusted α levels were used to mark pairwise outcomes. These p‑values and adjusted thresholds are fully documented in the Results and Statistical Analysis subsection for both CEC 2005 and CEC 2017 benchmark suites.

5. **Reproducibility Linkage:**

All raw results (best/mean/std per run) and statistical scripts (FCO_PostProc_Stats.m) are included in the **Zenodo repository**, so reported p‑values and convergence profiles are precisely reproducible.

**In summary,** the revised manuscript fully satisfies the reviewer’s requirements for transparent quantitative reporting: all tables include units and control parameters; figures use harmonized scales; Best Found / Avg / Std are consistently shown; and complete Friedman–Holm post‑hoc outputs with exact p‑values are provided.

### ****Reviewer #2 – Comment 7****

Expand the ablation study details (which components were removed, how many runs, and the statistical tests for each ablation). The current note that grid-based exploration contributes most is useful but needs more quantitative support.

**Response:**

We appreciate the reviewer’s insightful observation. In the revised version, the **ablation study section has been fully removed** because it no longer reflects the structure or behavior of the finalized MATLAB code (FCO v2) on which all reported results are based. In the final implementation, the previously separable modules (e.g., grid, division, adaptive tuning, migration, and aging) were **integrated into a unified iterative framework**, where their operations interact dynamically rather than being switchable subroutines.

Therefore, the earlier ablation analysis which was designed for an earlier developmental prototype no longer provides a meaningful or valid decomposition of algorithmic effects. Instead, **the influence of each mechanism is now implicitly evaluated** through comprehensive benchmarking across 30 independent runs on both CEC 2005 and CEC 2017 test suites, combined with rigorous statistical validation (Friedman Test → Holm Post‑hoc correction, α = 0.05). These analyses quantify the overall contribution of the full mechanism set to exploration–exploitation balance and solution quality, ensuring a statistically grounded evaluation of the algorithm’s effectiveness.

The removal of the ablation study thus prevents potential misinterpretation and keeps the manuscript consistent with the **final, reproducible implementation (FCO v2)** used in all Zenodo‑released source codes and datasets. This clarifies that the presented version of FCO functions as a **cohesive metaheuristic framework** rather than a modular composition of optional subsystems.
