# Supplementary material for: Felis Catus Optimization (FCO): A novel nature‑inspired metaheuristic algorithm
Source: PLoS One. 2026 Apr 15;21(4):e0341325. doi: 10.1371/journal.pone.0341325 (PMC13082733; doi:10.1371/journal.pone.0341325)
Supplement: Attachment — Submitted filename: PLOS ONE Rebuttal Letter2.docx [file pone.0341325.s009.docx]

**PLOS ONE Rebuttal Letter 2**

PONE-D-25-48643

**Response to Reviewers – Manuscript ID:** PONE-D-25-48643

**Manuscript Title: Felis Catus Optimization (FCO): A Novel Nature‑inspired Metaheuristic Algorithm.**

**Dear Academic Editor and Reviewers,**

We sincerely thank you for the thorough review and constructive feedback, which significantly improved the quality of our manuscript. Following your comments, we have carefully revised the paper. All changes are marked in the Track Changes version. Below we respond point-by-point.

**Reviewer #1 - Comment 1**

*Overall, the idea is promising, but it needs significant revision. The MATLAB code does not implement the described grid or territory model. Either create a formal grid (including cells, dominance, adjacency, and redistribution), or revise the text to reflect the actual ecological dynamics. Improve or correct the baselines, fix code issues, ensure full reproducibility, and update figures and tables (including Table 5). Please submit the updated code and regeneration scripts along with the revision*

**Response:**

We sincerely thank the reviewer for highlighting the importance of maintaining a clear and fully coherent correspondence between the descriptive formulation of the algorithm and its MATLAB implementation. Following this valuable recommendation, we carefully revisited the relevant sections of the manuscript with the primary goal of **improving clarity, eliminating potential ambiguities, and ensuring that the narrative description is expressed in a manner fully consistent with the continuous‑space implementation used in FCO v2**.

It is important to emphasize that **the underlying algorithmic structure has not been altered**. The MATLAB implementation already reflected the finalized continuous formulation, and therefore **no changes were necessary in the uploaded code base, in the minimal script archived on Zenodo, or in any of the numerical results and benchmark tables derived from it**. All experimental outcomes remain exactly reproducible using the publicly available FCO v2 package.

The revisions in the manuscript focus primarily on **terminology refinement and the alignment of explanations with the code’s continuous search‑space behavior**, rather than modifying any mechanisms. Specifically, references to earlier prototype concepts (e.g., grid, territory cells, or discrete adjacency) have been **rephrased or removed where necessary**, allowing the text to clearly convey the implemented model while preserving the original scientific narrative. These updates make the algorithm easier to understand and prevent possible misinterpretation, without introducing any methodological change.

To further support transparency and conceptual coherence, **all tables and figures including Table 5 as well as all schematic illustrations were systematically reviewed and updated** to ensure full consistency between the descriptive text, mathematical equations, and the implemented behavior of FCO v2. Captions and terminology were also harmonized accordingly.

We sincerely appreciate the reviewer’s insightful comments, which have directly contributed to improving the precision, clarity, and reproducibility of the manuscript. The revised version now offers a more accessible and fully synchronized presentation of the algorithm, its implementation, and its experimental evaluation.

### ****Reviewer #2 – Comment 1****

*The revised report has been thoroughly reviewed, and I can confirm that all previous comments have been successfully addressed.*

**Response:**

We sincerely thank you for your time and careful evaluation of the revised manuscript. We are pleased that all previous comments have been fully addressed to your satisfaction. We truly appreciate your constructive feedback, which significantly contributed to improving the clarity and quality of the paper.
